# A comparison of impact of comorbidities and demographics on 60-day mortality in ICU patients with COVID-19, sepsis and acute respiratory distress syndrome

**DOI:** 10.1038/s41598-022-19539-0

**Published:** 2022-09-20

**Authors:** Björn Ahlström, Robert Frithiof, Ing-Marie Larsson, Gunnar Strandberg, Miklos Lipcsey, Michael Hultström

**Affiliations:** 1 Anesthesiology and Intensive Care, Department of Surgical Sciences, Uppsala University, Uppsala, Sweden; 2 Region Dalarna, Centre for Clinical Research Dalarna, Falun Hospital, Nissers väg 3, 79182 Falun, Sweden; 3 Hedenstierna Laboratory, CIRRUS, Anesthesiology and Intensive Care, Department of Surgical Sciences, Uppsala University, Uppsala, Sweden; 4 Integrative Physiology, Department of Medical Cell Biology, Uppsala University, Uppsala, Sweden

**Keywords:** Viral infection, Bacterial infection, Respiratory distress syndrome

## Abstract

Severe Coronavirus disease 2019 (COVID-19) is associated with several pre-existing comorbidities and demographic factors. Similar factors are linked to critical sepsis and acute respiratory distress syndrome (ARDS). We hypothesized that age and comorbidities are more generically linked to critical illness mortality than a specific disease state. We used national databases to identify ICU patients and to retrieve comorbidities. The relative importance of risk factors for 60-day mortality was evaluated using the interaction with disease group (Sepsis, ARDS or COVID-19) in logistic regression models. We included 32,501 adult ICU patients. In the model on 60-day mortality in sepsis and COVID-19 there were significant interactions with disease group for age, sex and asthma. In the model on 60-day mortality in ARDS and COVID-19 significant interactions with cohort were found for acute disease severity, age and chronic renal failure. In conclusion, age and sex play particular roles in COVID-19 mortality during intensive care but the burden of comorbidity was similar between sepsis and COVID-19 and ARDS and COVID-19.

## Introduction

Coronavirus disease 2019 (COVID-19), caused by severe acute respiratory syndrome coronavirus 2 (SARS-CoV-2), has overwhelmed intensive care units (ICUs) worldwide beginning in late 2019. The beta coronavirus SARS-CoV-2 enters cells human by binding spike proteins to the angiotensin-converting enzyme 2, a receptor abundantly found on airway epithelial cells, pneumocytes and enterocytes of the small intestine^1^. The most prominent feature of severe COVID-19 is respiratory failure associated with alveolar inflammation and subsequent fibrosis^2^. Early reports from China suggested several comorbidities and demographic variables as risk factors for severe disease or death in or outside the ICU^3^.

The sepsis syndrome comprises a large proportion of ICU bed usage and ICU mortality^4^ and is commonly defined as a “life-threatening organ dysfunction caused by a dysregulated host response to infection”^5^. Since 2016 the syndrome is divided into sepsis (formerly severe sepsis) and septic shock with increasing mortality.

Acute respiratory distress syndrome^6^ (ARDS) is a syndrome of acute lung injury caused by inflammation that leads to pulmonary edema progressing to pulmonary consolidation and, if the inflammation is not resolved, eventually fibrosis. ARDS can be caused by pulmonary processes e.g., pneumonia and inhalation injury or by external inflammation related to, for example, major trauma or non-pulmonary sepsis^7^.

The outcomes of COVID-19, sepsis and ARDS are intimately correlated with age^8–10^ and, in the cases of COVID-19 and sepsis, also acute disease severity at admission^11,12^. However, although risk factors for adverse outcome in COVID-19 have been quantified previously, the importance of specific comorbidities in COVID-19 compared to other forms of critical illness have not previously been analyzed^13–15^. Similar risk factors are evident in sepsis and ARDS, and published data do not support the interpretation that ICU patients with COVID-19 are more burdened by comorbidity^12,16^.

We used the Swedish intensive care registry to compare COVID-19 patients to historical controls with sepsis (i.e. severe sepsis or septic shock) or ARDS to test the relative importance of demographics and comorbidity. We hypothesized that advanced age and comorbidity are signs of reduced physiological compensatory capacity causing patients to be more prone to die within 60 days from admission to critical care for any given illness. Therefore, aging, sex and comorbidity should be equally associated with death in COVID-19, sepsis, or ARDS.

## Methods

In this cohort study we aimed to investigate the relative importance of comorbidities, age and sex for the odds of death within 60 days of ICU admission (60-day mortality) in COVID-19, sepsis and ARDS. 60-day mortality is an established mortality measure in COVID-19^17^. The study was approved by the Regional Ethics Committee of Uppsala (approval no. 2016/421) and the Swedish Ethical Review Authority (approval no. 2020-02144). Informed consent was waived by the same authority because of the nature of the study. We registered the study *à priori* at ClinicalTrials.gov (NCT04542538) and the research was conducted in accordance with the Declaration of Helsinki with subsequent revisions. Reporting follows the STROBE (strengthening the reporting of observational studies in epidemiology) guidelines^18^.

### Data sources

All general and most specialist ICUs report all admissions to the Swedish intensive care registry (SIR)^19,20^. The national patient registry (NPR), a research support tool, was established by the Swedish Board of Health and Welfare and reporting is governed by statutory and common law^21^. We collected data on ICU diagnoses, demographics, ICU care and mortality from the SIR and we received data on comorbidities reported in the five years preceding ICU admission from the in-patient sub-registry of the NPR for all patients.

### Disease groups

We compared three groups of adult (age ≥ 18 years) ICU patients diagnosed with COVID-19, sepsis or ARDS. Sepsis was defined as severe sepsis or septic shock according to the Sepsis 2 criteria^22^, coded with International Statistical Classification of Diseases and Related Health Problems—tenth edition (ICD-10) A49.9, R65.1 or R57.2 in the SIR. ARDS was defined according to the American-European consensus conference on the ARDS definition^23^, 2011–2015, or the Berlin definition^6^, 2016 and coded with ICD-10 J80.9 × in the SIR. All ICU-admitted adult COVID-19 patients in Sweden from 6 February 2020 to 16 June 2021 were identified by ICD-10 code U07.1 in the SIR while virtually all Swedish ICU-admitted adult patients with severe sepsis, septic shock or ARDS were identified in the SIR from 2011 to 2016.

Any single Sepsis patient could be included in the ARDS cohort and vice versa*.* Accordingly, ARDS patients were non-COVID-19 ARDS patients and Sepsis patients were non-COVID-19 Sepsis patients. Patients were only included for their first admission for COVID-19, Sepsis, or ARDS. However, as the COVID-19 group stems from a separate time period a patient could be included in both the COVID-19 and Sepsis or ARDS groups. Exclusion criteria were lack of personal identification number and age < 18 years. ICU care episodes ending and starting in the same 24-h period were merged.

### Statistics

Data are reported as medians with interquartile range (IQR) or number with percent in brackets. The primary outcomes were the relative importance of age, sex and comorbidities (Table S1) for 60-day mortality in COVID-19, Sepsis or ARDS.

The relative importance of age, sex, Simplified Acute Physiology Score 3 (SAPS3)^24^ Box III, and comorbidities were assessed as an interaction with the disease group (COVID-19 or Sepsis and COVID-19 or ARDS) using logistic regression. COVID-19 was compared separately to Sepsis and ARDS. A significant interaction between disease group and a variable indicates a difference in effect between groups for that variable. Because we added age and comorbidities in the models and treatments preceding ICU admission might be related to diagnosis the SAPS3 Box III, representing the acute physiologic derangement at ICU admission, was used. SAPS3^24^ is a risk score initially developed to perform risk adjusted comparisons of hospital mortality in ICU admitted patients between and within ICUSs, but is now widely used and validated also for 30- and 90-day mortality^25,26^.

We used restricted cubic splines in all continuous variables, age and SPAS3 Box III, as we could not rule out a non-linear relationship with the logit of outcome. To estimate individual risk factor p-values a linear representation of the variable was applied to the model adjusted for the splined variables. We found 14 marginally influential observations in the model on 60-day mortality in COVID-19 and ARDS using the rms-package. We found indications of multicollinearity in relation to age and SAPS3 Box III for all models. SAPS3 data was missing in 414 patients (1.3%), who were excluded from 60-day mortality modelling. Due to an imbalance between groups for the different hospital types, hospital type was added to the models.

Statistical significance was defined as p-value < 0.05 (two-sided). In analysis of crude differences between disease groups we used the Mann–Whitney U-test and Chi^2^-test as appropriate with Bonferroni-correction because of multiple comparisons. Odds ratios (ORs) were calculated between the 25th and 75th percentiles in variables for which restricted cubic splines were applied, *i.e.* age and the SAPS3 Box III. Data management and descriptive statistics were performed in SPSS for Windows version 27 (Microsoft Corp., IL, USA). For multiple imputations, regression models and graphics, we used the R Software version 4.0.3 (The R Foundation for Statistical Computing, Vienna, Austria; https://www.r-project.org) with the mice, rms, Hmisc, and forest plot packages.

### Sensitivity analyses

The specification and rationale for the performed sensitivity analyses are found in Supplementary Table S2 online.

## Results

**Figure 1:**
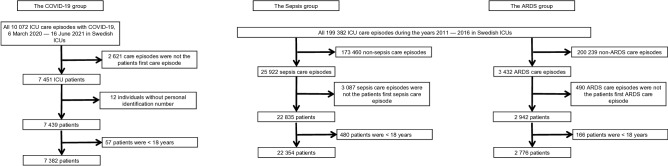
Patient selection flowchart. Patients 18 years or older that were admitted to Swedish ICUs were selected for this study from the Swedish Intensive Care Registry. COVID-19 patients admitted between 6 March and 16 June 2021 were included. Patients with non-COVID-19 sepsis or non-COVID-19 ARDS from 2011 to 2016 were included as controls. *COVID-19* Corona virus disease 2019, *ICU* Intensive care unit, *ARDS* Acute respiratory distress syndrome.

At data acquisition, 7382 consecutive adult COVID-19 ICU patients were enrolled from the SIR. Of the COVID-19 patients, 1389 (19%) were also coded with Sepsis and 5491 (74%) were also coded with ARDS during intensive care. Of the 22,354 adult patients included in the Sepsis group, 1100 were also included in the ARDS group, with a total of 2776 patients, and vice versa (Table 1, Fig. 1). The COVID-19 patients had a numerically lower percentage of women, younger age, a lower SAPS3 and a lower median updated Charlson comorbidity index (CCI)^27^ than the Sepsis and ARDS patients.

**Table 1:** Baseline characteristics of patients included in the COVID-19, sepsis and ARDS cohorts.

	Sepsis patients admitted to ICU	COVID-19 patients admitted to ICU	ARDS patients admitted to ICU
**Number of patients**	22,354	7382	2776
With COVID-19	0 (0)	7382 (100)	0 (0)
With sepsis	–	1389 (18.8)	1100 (39.6)
With ARDS	1100 (4.9)	5491 (74.0)	–
Female sex	9500 (42.5)	2191 (29.7)	1033 (37.2)
Age at ICU-admission (years)	70 (60–78)	63 (53–72)	65 (53–74)
**Hospital type**			
University	5676 (25.4)	2566 (34.8)	1167 (42.0)
County	11,080 (49.6)	3749 (50.8)	1211 (43.6)
District	5598 (25.0)	1067 (14.5)	398 (14.3)
SAPS3	66 (57–76)	54 (48–61)	66 (57–76)
CCI	1 (0–3)	0 (0–1)	1 (0–2)
Surgical admission	2468 (11.3)	130 (1.8)	176 (6.3)

Baseline characteristics of patients ≥ 18 years old admitted to Swedish ICUs, with COVID-19, between 6th of March and 16th of June 2021 or admitted to Swedish ICUs with non-COVID-19 Sepsis or non-COVID-19 ARDS between the years 2011 and 2016. Data are presented as numbers with percentages or medians with interquartile ranges as appropriate.

*ICU* intensive care unit, *COVID-19* Coronavirus disease 2019, *SAPS3* Simplified Acute Physiology Score 3^24^, *CCI* updated Charlson Comorbidity Index^27^.

The 60-day mortality was significantly lower in the COVID-19 patients (27.5%), than in the Sepsis (34.1%) and the ARDS patients (45%). ICU-length of stay was longer for COVID-19 than Sepsis patients and the use of invasive mechanical ventilation was more common in COVID-19 than Sepsis patients, but lower in COVID-19 than ARDS patients. The crude proportion of all studied comorbidities was lower in the COVID-19 than in the Sepsis group. Compared to the ARDS group the COVID-19 group had a lower crude proportion of all studied comorbidities, except type 2 diabetes mellitus (T2DM), chronic renal failure, asthma, and obesity (Table 2). Demographics and comorbidity data of the deceased patients by disease group are summarized in Supplementary Table S4 online.

**Table 2:** Outcome and comorbidities of patients included in the COVID-19, sepsis and ARDS cohorts.

	Sepsis admitted to ICU	p	COVID-19 admitted to ICU	p	ARDS admitted to ICU
Number of patients	22,354		7382		2776
Died within 60-days from ICU admission	7631 (34.1)	< 0.001	2029 (27.5)	< 0.001	1249 (45.0)
ICU length of stay	2.63 (1.1–6.7)	< 0.001	7.71 (3.2–17.6)	0.22	8.9 (4.0–17.7)
Invasive mechanical ventilation	8494 (38.4)	< 0.001	1074 (60.7)	< 0.001	2160 (77.8)
Ischemic heart disease	4352 (19.5)	< 0.001	518 (7.0)	< 0.001	383 (13.8)
Non-ischemic heart disease	7025 (31.4)	< 0.001	812 (11.0)	< 0.001	602 (21.7)
Hypertension	9504 (42.5)	< 0.001	1739 (23.6)	< 0.001	913 (32.9)
Diabetes mellitus type 1	1398 (6.3)	< 0.001	74 (1.0)	< 0.001	140 (5.0)
Diabetes mellitus type 2	4408 (19.7)	< 0.001	944 (12.8)	0.81	397 (14.3)
Stroke	2845 (12.7)	< 0.001	211 (2.9)	< 0.001	260 (9.4)
Renal failure	1664 (7.5)	< 0.001	282 (3.8)	> 0..99	124 (4.5)
COPD	2344 (10.5)	< 0.001	279 (3.8)	< 0.001	171 (6.2)
Asthma	1266 (5.7)	< 0.001	306 (4.1)	> 0.99	125 (4.5)
Obesity	1243 (5.6)	0.009	333 (4.5)	> 0.99	106 (3.8)
Immunosuppressed	661 (3.0)	< 0.001	38 (0.5)	< 0.001	131 (4.7)
Cancer	2257 (10.1)	< 0.001	122 (1.7)	< 0.001	242 (8.7)
Hematological malignancy	986 (4.4)	< 0.001	78 (1.1)	< 0.001	192 (6.9)
Inflammatory disease	2412 (10.8)	< 0.001	283 (3.8)	< 0.001	246 (8.9)
Solid organ transplant recipient	395 (1.8)	0.015	88 (1.2)	0.036	56 (2.0)

Outcome and comorbidities of patients ≥ 18 years old admitted to Swedish ICUs, with COVID-19, between 6th of March and 16th of June 2021 or admitted to Swedish ICUs with non-COVID-19 Sepsis or non-COVID-19 ARDS between the years 2011 and 2016. Data are presented as numbers with percentages or medians with interquartile range.

*COVID-19* Corona virus disease 2019, *ARDS* Acute respiratory distress syndrome, *ICU* intensive care unit, *COPD* chronic obstructive pulmonary disease. *P* P-value, after Bonferroni adjustment, for difference between adjacent columns.

### Logistic modelling

**Figure 2:**
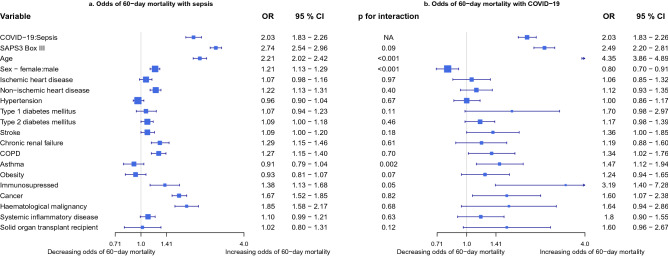
Risk factors for 60-day mortality in Sepsis compared to COVID-19. Odds of 60-day mortality with sepsis (**a**) or COVID-19 (**b**) based on comorbidity in a logistic regression model. Sepsis is severe sepsis or septic shock without COVID-19. A p-value for interaction < 0.05 denotes a significant interaction of the risk factor with the disease cohort and indicates risk factors with differential effect between sepsis and COVID-19. *ICU* Intensive care unit, *COVID-19* Corona virus disease 2019, *p* p-value, *OR* Odds ratio, *CI* Confidence interval, *SAPS3 Box III* adjusted Simplified acute physiology score 3 Box III^24^, *COPD* Chronic obstructive pulmonary disease. Also in model: Hospital type District—County: 0.84 (0.78–0.90), OR (95% CI) and University—County: 0.92 (0.86–0.98).

**Figure 3:**
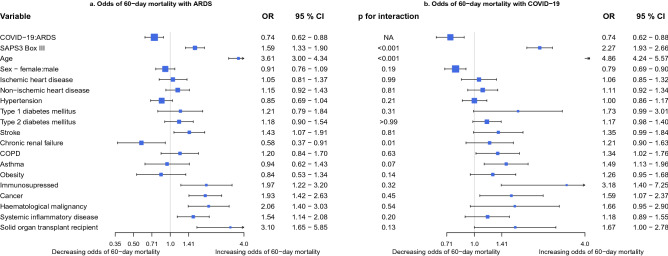
Risk factors for 60-day mortality in ARDS compared with COVID-19. Odds of 60-day mortality with ARDS (**a**) or COVID-19 (**b**) based on comorbidity in a logistic model. ARDS is ARDS without COVID-19. A p-value for interaction < 0.05 denotes a significant interaction of the risk factor with the disease cohort and indicates risk factors with differential effect between ARDS and COVID-19. *ICU* Intensive care unit, *COVID-19* Corona virus disease 2019, *ARDS* Acute respiratory distress syndrome, *p* p-value, *OR* Odds ratio, *CI* Confidence interval, *SAPS3 Box III* adjusted Simplified acute physiology score 3 Box III^24^, *COPD* Chronic obstructive pulmonary disease. Also in model: Hospital type District—County: 0.85 (0.73–0.97), OR (95% CI) and University—County: 0.83 (0.75–0.92).

The interaction between the disease group and the individual risk factors in a logistic regression was used to assess the differential effect between COVID-19 and Sepsis, or ARDS. Between COVID-19 and Sepsis the interaction was significant for age (p < 0.001), sex (p < 0.001), and asthma (p = 0.002), indicating a stronger association between age, male sex and asthma with 60-day mortality in COVID-19 than in Sepsis (Fig. 2). In the model on COVID-19 and ARDS the interaction was significant for SAPS3 Box III (p < 0.001), age (p < 0.001) and chronic renal failure (p = 0.001), indicating a stronger association of SAPS3 Box III, age and chronic renal failure to 60-day mortality in COVID-19 than in ARDS (Fig. 3).

### Sensitivity analyses

Results for the sensitivity analyses are summarized in Supplementary Table S3 and presented in detail in Supplementary Tables S5–S15 online. Some differences between the results of the main analyses and those of the sensitivity analyses were seen, however adding a variable denoting time since first inclusion to the model of 60-day mortality in COVID-19 and Sepsis did not affect the inferences (Supplementary Table S5 online). In the analyses where missing SAPS3 Box III was imputed using the mice() function there were no changes in the significance of the interactions (Supplementary Tables S6 and S7 online). In the two sensitivity analyses where patients included in both the Sepsis and ARDS groups were excluded there were no differences for the model including COVID-19 and Sepsis patients (Supplementary Table S8 online). However, in the model on COVID-19 or ARDS the p-value for the interaction between the variable indicting disease group affiliation and age changed from < 0.001 to 0.06 (Supplementary Table S9 online). Excluding the SAPS3 Box III caused no changes in the model on COVID-19 and ARDS, however, the impact of obesity became differential between the COVID-19 and Sepsis groups as the p-value for the interaction with the disease group affiliation variable changed from 0.07 to 0.03 (Supplementary Tables S10 and S11 online). When performing the model on COVID-19 and ARDS without patients with overly influential observations the interaction with asthma became significant as the p-value decreased from 0.07 to 0.03 (Supplementary Table S12 online). All models were performed without the variable denoting hospital type with no change to the results. (Supplementary Tables S13 and S14 online). Finally, length of stay was inversely correlated to age in patients who died within 60 days, and there was a significant interaction with disease group, indicating that end-of-life decisions might have affected differences between outcome in COVID-19 and Sepsis (Supplementary Table S15 online). However, these differences did not affect the main conclusions.

## Discussion

The key finding of this study is that almost all comorbidities under investigation were not of greater importance for mortality in COVID-19 compared to in Sepsis and ARDS. This finding is in support of our hypothesis that comorbidities are general risk factors for critical illness mortality, not a specific etiological factor for critical COVID-19 mortality.

While almost all comorbidities under investigation were of similar importance with regard to 60-day mortality in COVID-19 and Sepsis as well as in COVID-19 and ARDS, we found a differential effect for asthma. Asthma showed a stronger association to 60-day mortality in COVID-19 than in Sepsis possibly relating to previous evidence that asthma is associated to a better prognosis in Sepsis^28^. This finding is also consistent with a previous study in which an independent association was found between asthma and COVID-19 ICU-mortality^15^. Between COVID-19 and ARDS no differential effect was found for asthma, possibly linked to a common pulmonary pathophysiology in COVID-19 and ARDS^29^. We also found a differential effect for chronic renal failure showing a protective effect regarding 60-day mortality in ARDS but not in COVID-19.

Of note, our data show a greater association for age to 60-day mortality in COVID-19 than in Sepsis or ARDS. The stronger association of advanced age to 60-day mortality in COVID-19 than in Sepsis and ARDS is in line with several studies recognizing the importance of age in the prognosis of COVID-19^12,15,30,31^. Moreover, the association to COVID-19 mortality might also be linked to a greater tendency to use of end-of-life decisions in COVID-19 than in Sepsis and ARDS in the aged patients. This tendency is indicated by an interaction between disease group and age in a linear regression model on length of stay in deceased patients presented in Supplemental Table S15 online. We found that the association between female sex and 60-day mortality was higher in Sepsis than in COVID-19. The difference might depend on a higher risk of death in women with Sepsis compared to men, which is under discussion^12,32^. This contrasts to COVID-19 in which several investigators have found no strong association between sex and ICU mortality^15,33,34^. However, there are studies where the effect of sex is more pronounced^35,36^ and the protective effect of female sex in COVID-19 is an area of current investigation^37^.

The greatest strength of this study is the high-quality datasets on which it is based and the large sample cohorts it examines. A second strength concerns the robustness of our outcome measure, i.e. 60-day mortality where the follow-up can be expected to be complete given the Swedish personal identification number system. A third strength is the low frequency of missing data, which could presumably be missing at random because of the nature of our data. This reasoning implies that model-based imputation can be expected to perform well. We assessed the stability of the results in regard to missing data by performing a sensitivity analysis based on the complete dataset after multiple imputation by chained equations, which did not change the main findings. In addition, possible bias related to Sepsis patients also diagnosed with ARDS, and vice versa*,* was assessed through sensitivity analyses excluding these patients. This analysis only impacted the effect of the COPD variable in the 60-day mortality models on ARDS and COVID-19 patients. However, excluding all Sepsis patients from the ARDS group meant a reduction in sample size by almost one half. We found indications of multicollinearity in association to SAPS 3. When we performed sensitivity analyzes without SAPS 3 box III the impact on model results was small and thus we feel confident in model stability in this regard. Finally, we address the possible reduced risk-adjusted mortality over time in Sepsis patients defined by the sepsis-2 criteria^12,32^ using a sensitivity analysis including time as a covariate without effect on the results.

As a registry study, some inherent limitations may be more prominent during the ongoing pandemic. The registries are monitored continuously and amended, but data for the COVID-19 group was reported during the ongoing surges and may include more errors than the historical controls. The unexpectedly low frequency of ARDS- and sepsis-coding in the COVID-19 group is likely due to several causes: (1) in 39% of the patients invasive mechanical ventilation was not performed; and (2) there was an unusually low quality of diagnosis reporting during the peak of the surge. Moreover, our Sepsis group is defined according to the 2001 sepsis definition, which differs somewhat from the sepsis-3 definition^5^. However, these concerns should not affect data related to the primary outcomes in the study or the registration of exposures relevant to this study. Finally, epidemiological factors may partly explain the observed differences in the distribution of risk factors. Many middle-aged individuals with limited comorbidity have been exposed to and infected with the SARS-COV-2 virus in the community, where some have developed a critical illness. Numerous older individuals with more pronounced comorbidity have practiced strict isolation and may have avoided infection, whereas the very old and frail, usually infected in nursing homes, are seldom admitted to intensive care. Finally, we found indications of multicollinearity in association to age. However, we believe it would be pointless to perform the models without the age variable, as age is such a strong risk factor for organ dysfunction and death in modeling by us and others^8,11^.

We conclude that the burden of comorbidity is similar for 60-day mortality after ICU admission with COVID-19, Sepsis and ARDS. Age is a more decisive risk factor in COVID-19 than in Sepsis and ARDS.

## Supplementary Information

Supplementary Information.

## Data Availability

The data that support the findings of this study are available from the respective national registries with restrictions as defined by the General Data Protection Regulation (GDPR), the Swedish Personal Data Act (1998:204), and the licenses with the respective national registries, and so are not publicly available. Data are however available from the authors upon reasonable request after adequate permissions from the Swedish ethical review authority and under the restrictions outlined above.
