# Supplementary material for: A comparison of impact of comorbidities and demographics on 60-day mortality in ICU patients with COVID-19, sepsis and acute respiratory distress syndrome
Source: Sci Rep. 2022 Sep 20;12:15703. doi: 10.1038/s41598-022-19539-0 (PMC9487845; doi:10.1038/s41598-022-19539-0)
Supplement: Supplementary file 1 — Supplementary Information. [file 41598_2022_19539_MOESM1_ESM.pdf]

Supplementary Information

A Comparison of impact of comorbidities and demographics on 60-day mortality in ICU patients with COVID-19, Sepsis and Acute Respiratory Distress syndrome

Björn Ahlström, M.D.[1, 2]*, Robert Frithiof, M.D[1], Ing-Marie Larsson, R.N[1], Gunnar Strandberg, M.D[1], Miklos Lipcsey, MD [1, 3], Michael Hultström, MD[1, 4]

[1]Anesthesiology and Intensive Care, Department of Surgical Sciences, Uppsala University, Uppsala, Sweden. [2]Region Dalarna, Centre for Clinical Research Dalarna, Falun, Sweden [3],Hedenstierna laboratory, CIRRUS, Anesthesiology and Intensive Care, Department of Surgical Sciences, Uppsala University, Uppsala, Sweden, [4]Integrative Physiology, Department of Medical Cell Biology, Uppsala University, Uppsala, Sweden.

* Corresponding author

Supplementary Table S1 2

Supplementary table S2 3

Supplementary table S3 4

Supplementary Table S4 5

Supplementary Table S5 6

Supplementary Table S6 7

Supplementary Table S7 8

Supplementary Table S8 9

Supplementary Table S9 10

Supplementary Table S10 11

Supplementary Table S11 12

Supplementary Table S12 13

Supplementary Table S13 14

Supplementary Table S14 15

Supplementary Table S15 16

References 17

Model code R 18

Imputation 18

60-day mortality main model 19

Data management SPSS 23

SUPPLEMENTARY TABLE S1. Variable definitions based on ICD-10 codes. For ICD-10 codes, any occurrence in the 5 years preceding inclusion was used, except for cancer where occurrence within 12 months was stipulated.

Ischemic heart disease	I20.x-I25.x	
Non-ischemic heart disease	I30.x-I52.x + I01.x, I05.x-I09.x, I11.x, I13.x, I27.0	
Type 1 diabetes mellitus	E10.x	
Type 2 diabetes mellitus	E11.x	
Stroke	I60.x-I69.x	
Chronic renal failure	N18	or intervention DR016: hemodialysis, DR024: peritoneal dialysis within 5 years preceding inclusion
COPD	J41-x-J44-x	
Asthma	J45.x-J46.x	
Obesity	E66	or intervention JDF or JFD03: bariatric surgery within 5 years preceding inclusion,
Immunosuppressed	D71.x, D80.x-D84.x, D89.x	or treatment DV069, DV070-DV072: radiation therapy, or DT108, DT112, DT116, DT135: cytotoxic treatment within 6 months preceding inclusion
Cancer	C0x.x-C9x.x	within 12 months
Inflammatory disease	M05.x-M14.x, M30.x_M36.x	
Solid organ transplant recipient	Z940, Z941, Z942, Z943, Z944	

ICD-10: International classification of diseases - tenth revision, COPD: chronic obstructive pulmonary disease.

SUPPLEMENTARY TABLE S2. Rational for sensitivity analyses.

Sensitivity analysis, adjusting for months after first inclusion (1 January 2011), was performed for 60-day mortality in COVID-19 and Sepsis to account for reduced risk-adjusted mortality over time in sepsis detected by some authors[1,2].

In addition, sensitivity analyses were performed on risk of 60-day mortality in which missing SAPS 3 Box III data were imputed into five separate datasets using the multivariate imputation by chained equations (mice) package. Regression outputs were pooled using the Harrel Miscellaneous (Hmisc) package in the R software.

We also performed the 60-day mortality models without SAPS3 Box III to address the indicated multicolinearity related to that variable.

Moreover, sensitivity analyses were undertaken by excluding Sepsis patients also included in the ARDS group and *vice versa,* to evaluate possible confounding by overlapping groups. In order to assess the impact of 14 overly influential observations a sensitivity analysis on 60-day mortality in COVID-19 and ARDS was performed after removing patients with these observations.

The models were also performed without the hospital type variable as hospital type was added to the models during the review process in a data driven fashion.

Finally, the possibly differential effect of earlier termination of ICU care in older patients, depending on disease group, was assessed in a linear regression of age on ICU length of stay in COVID-19 and Sepsis patients who died in the ICU.

COVID-19: Corona Virus Disease 2019, SAPS3: Simplified Acute Physiology Score 3[3], ARDS: Acute respiratory distress syndrome, ICU: intensive care unit

SUPPLEMENTARY TABLE S3. Results for sensitivity analyses.

Some differences between the results of the main analyses and those of the sensitivity analyses were seen, however adding a variable denoting time since first inclusion to the model of 60-day mortality in COVID-19 and Sepsis did not affect the inferences (Supplementary Table S5 online). In the analyses where missing SAPS3 Box III was imputed using the mice() function there were no changes in the significance of the interactions (Supplementary Table S6 and S7 online). In the two sensitivity analyses where patients included in both the Sepsis and ARDS groups were excluded there were no differences for the model including COVID-19 and Sepsis patients (Supplementary Table S8 online). However, in the model on COVID-19 or ARDS the p-value for the interaction between the variable indicting disease group affiliation and age changed from <0.001 to 0.06 (Supplementary Table S9 online). Excluding the SAPS3 Box III caused no changes in the model on COVID-19 and ARDS, however, the impact of obesity became differential between the COVID-19 and Sepsis groups as the p-value for the interaction with the disease group affiliation variable changed from 0.07 to 0.03 (Supplementary Table S10 and S11 online). When performing the model on COVID-19 and ARDS without patients with overly influential observations the interaction with asthma became significant as the p-value decreased from 0.07 to 0.03 (Supplementary Table S12 online). All models were performed without the variable denoting hospital type with no change to the results. (Supplementary Table S13 and S14 online). Finally, length of stay was inversely correlated to age in patients who died within 60 days, and there was a significant interaction with disease group, indicating that end-of-life decisions might have affected differences between outcome in COVID-19 and Sepsis (Supplementary Table S15 online).

COVID-19: Corona Virus Disease 2019, SAPS3: Simplified Acute Physiology Score 3[3], mice : multivariate imputation by chained equations, ARDS: Acute respiratory distress syndrome, ICU: intensive care unit

SUPPLEMENTARY TABLE S4. Baseline characteristics and comorbidities of patients who died within 60 days from ICU admission, divided by disease cohort.

	**Sepsis died in ICU**	**P**	**COVID-19 died in ICU**	**p**	**ARDS died in ICU**
Number of patients	7 631		2 029		1 249
Female sex	3 287 (43.1)	< 0.001	552 (27.2)	< 0.001	426 (34.1)
Age at ICU-admission (years)	73 (66 - 80)	< 0.001	71 (64 - 76)	> 0.99	70 (62 - 77)
Hospital type		< 0.001		< 0.001	
University	1 735 (27.7)		643 (31.7)		502 (40.2)
County	3 906 (51.2)		1 104 (54.4)		559 (44.8)
District	1 735 (22.7)		282 (13.9)		188 (15.1)
SAPS3	74 (66 - 84)	< 0.001	61 (55 - 68)	< 0.001	72 (63 - 81)
CCI	2 (0 - 4)	< 0.001	0 (0 - 2)	< 0.001	2 (0 - 3)
ICU length of stay	2.4 (0.9 - 6.8)	< 0.001	11.0 (3.7- 19.5)	< 0.001	8.0 (3.0 - 16.1)
Surgical admission	742 (9.9)	< 0.001	40 (2.0)	0.003	53 (4.3)
Ischemic heart disease	1 790 (23.5)	< 0.001	226 (11.1)	< 0.001	225 (18.0)
Non-ischemic heart disease	2 956 (38.7)	< 0.001	372 (18.3)	< 0.001	343 (27.5)
Hypertension	3 725 (48.8)	< 0.001	699 (34.5)	0.14	490 (39.2)
Diabetes mellitus type 1	521 (6.8)	< 0.001	28 (1.4)	< 0.001	67 (5.4)
Diabetes mellitus type 2	1 718 (22.5)	0.01	384 (18.9)	> 0.99	206 (16.5)
Stroke	1 149 (15.1)	< 0.001	96 (4.7)	< 0.001	159 (12.7)
Chronic renal failure	762 (10.0)	< 0.001	132 (6.5)	0.92	59 (4.7)
COPD	1 003 (13.1)	< 0.001	136 (6.7)	> 0.99	90 (7.2)
Asthma	432 (5.7)	> 0.99	116 (5.7)	> 0.99	52 (4.2)
Obesity	368 (4.8)	> 0.99	105 (5.2)	0.03	35 (2.8)
Immunosuppressed	309 (4.0)	< 0.001	21 (1.0)	< 0.001	87 (7.0)
Cancer	973 (12.8)	< 0.001	62 (3.1)	< 0.001	154 (12.3)
Hematological malignancy	505 (6.6)	< 0.001	45 (2.2)	< 0.001	126 (10.1)
Inflammatory disease	973 (12.8)	< 0.001	123 (6.1)	< 0.001	143 (11.4)
Solid organ transplant recipient	131 (1.7)	> 0.99	35 (1.7)	> 0.99	31 (2.5)

Data are presented as numbers with percentages or medians with interquartile range, as appropriate. ICU: intensive care unit, COVID-19: Corona Virus Disease 2019, ARDS: Acute respiratory distress syndrome, SAPS3: Simplified Acute Physiology Score 3[3], CCI: the updated Charlson comorbidity index[4], COPD: chronic obstructive pulmonary disease. P is P-value, after Bonferroni adjustment, for difference between adjacent columns.

SUPPLEMENTARY TABLE S5. A sensitivity analysis, binary logistic model on risk of mortality within 60 days of ICU admission with Sepsis or COVID-19. Significance for the interaction with a variable denoting intensive care with non-COVID-19 Sepsis or COVID-19. A variable denoting time since first inclusion was added.

	Sepsis patients			COVID-19 patents			
	HR	95 % CI		HR	95 % CI		P for interaction
Time	0.92	0.86	0.99	0.92	0.86	0.99	NA
COVID-19 - Sepsis: COVID-19	2.34	1.99	2.75	2.34	1.99	2.75	NA
SAPS3 Box III	2.74	2.54	2.96	2.49	2.20	2.81	0,09
Age	2.22	2.03	2.42	4.36	3.87	4.90	<0,001
Sex - female: male	1.21	1.13	1.29	0.80	0.70	0.91	<,0001
Ischemic heart disease	1.06	0.98	1.15	1.06	0.85	1.32	0,98
Non-ischemic heart disease	1.22	1.13	1.31	1.12	0.93	1.35	0,40
Hypertension	0.97	0.90	1.04	1.00	0.86	1.17	0,70
Type 1 diabetes mellitus	1.07	0.93	1.22	1.70	0.98	2.97	0,11
Type 2 diabetes mellitus	1.09	1.00	1.19	1.17	0.98	1.39	0,47
Stroke	1.09	1.00	1.20	1.36	1.00	1.85	0,18
Chronic renal failure	1.30	1.15	1.46	1.19	0.88	1.60	0,60
COPD	1.27	1.15	1.40	1.34	1.02	1.76	0,70
Asthma	0.91	0.80	1.04	1.47	1.12	1.94	0,002
Obesity	0.94	0.81	1.08	1.24	0.94	1.65	0,08
Immunosuppressed	1.38	1.13	1.68	3.19	1.40	7.29	0,05
Cancer	1.67	1.51	1.85	1.60	1.07	2.38	0,82
Hematological malignancy	1.85	1.57	2.16	1.64	0.94	2.86	0,69
Systemic inflammatory disease	1.10	0.99	1.21	1.18	0.90	1.55	0,63
Solid organ transplant recipient	1.02	0.79	1.30	1.61	0.97	2.67	0,11
Hospital type - district: county	0.84	0.78	0.90	0.84	0.78	0.90	NA
Hospital type - university: county	0.92	0.86	0.98	0.92	0.86	0.98	NA

ICU: Intensive care unit, COVID-19: Corona Virus Disease 2019, HR: Hazard ratio, CI: Confidence interval, NA: Not applicable, SAPS3 Box III: Simplified Acute Physiology Score 3 Box III[3], COPD: Chronic obstructive pulmonary disease, P: P-value

SUPPLEMENTARY TABLE S6. A sensitivity analysis, Binary logistic model on risk of mortality within 60 days of ICU admission with Sepsis or COVID-19. Significance for the interaction with a variable denoting intensive care with non-COVID-19 Sepsis or COVID-19. 414 imputations for missing SAPS 3 Box III were performed.

	**Sepsis patients**			**COVID-19 patents**			
	HR	95 % CI		HR	95 % CI		P for interaction
COVID-19 - Sepsis: COVID-19	2.00	1.81	2.22	2.00	1.81	2.22	NA
SAPS3 Box III	2.73	2.53	2.94	2.49	2.21	2.81	0.11
Age	2.21	2.02	2.41	4.34	3.85	4.88	<0.001
Sex - female: male	1.21	1.14	1.29	0.80	0.70	0.91	<0.001
Ischemic heart disease	1.07	0.99	1.16	1.07	0.86	1.33	0.99
Non-ischemic heart disease	1.21	1.13	1.30	1.11	0.92	1.34	0.38
Hypertension	0.96	0.90	1.03	1.00	0.86	1.17	0.64
Type 1 diabetes mellitus	1.07	0.94	1.23	1.70	0.97	2.96	0.12
Type 2 diabetes mellitus	1.09	1.00	1.18	1.18	0.99	1.40	0.43
Stroke	1.10	1.00	1.20	1.36	1.00	1.85	0.20
Chronic renal failure	1.29	1.14	1.45	1.19	0.88	1.60	0.63
COPD	1.26	1.14	1.39	1.33	1.02	1.75	0.69
Asthma	0.91	0.79	1.04	1.49	1.13	1.96	0.002
Obesity	0.92	0.80	1.06	1.24	0.93	1.64	0.07
Immunosuppressed	1.38	1.14	1.68	2.87	1.27	6.44	0.09
Cancer	1.67	1.51	1.84	1.60	1.08	2.39	0.84
Hematological malignancy	1.85	1.58	2.16	1.68	0.98	2.89	0.75
Systemic inflammatory disease	1.10	1.00	1.21	1.19	0.91	1.56	0.60
Solid organ transplant recipient	1.06	0.83	1.35	1.59	0.96	2.65	0.16
Hospital type - district: county	0.84	0.78	0.90	0.84	0.78	0.90	NA
Hospital type - university: county	0.93	0.87	0.99	0.93	0.87	0.99	NA

ICU: Intensive care unit, COVID-19: Corona Virus Disease 2019, HR: Hazard ratio, CI: Confidence interval, NA: Not applicable, SAPS3: Simplified Acute Physiology Score 3 Box III[3] COPD: Chronic obstructive pulmonary disease, P: P-value

SUPPLEMENTARY TABLE S7. A sensitivity analysis, Binary logistic model on risk of mortality within 60 days of ICU admission with ARDS or COVID-19. Significance for the interaction with a variable denoting intensive care with non-COVID-19 ARDS or COVID-19. 414 imputations for missing SAPS 3 Box III were performed.

	**ARDS patients**			**COVID-19 patents**			
	HR	95 % CI		HR	95 % CI		P for interaction
COVID-19 - Sepsis: COVID-19	0.74	0.62	0.87	0.74	0.62	0.87	NA
SAPS3 Box III	1.60	1.34	1.91	2.26	1.93	2.65	<0.001
Age	3.59	2.99	4.32	4.86	4.25	5.57	<0.001
Sex - female: male	0.90	0.76	1.08	0.79	0.69	0.90	0.24
Ischemic heart disease	1.08	0.84	1.40	1.06	0.86	1.32	0.92
Non-ischemic heart disease	1.12	0.90	1.40	1.10	0.91	1.33	0.90
Hypertension	0.85	0.70	1.05	1.00	0.86	1.17	0.23
Type 1 diabetes mellitus	1.17	0.77	1.78	1.72	0.99	3.00	0.28
Type 2 diabetes mellitus	1.16	0.89	1.52	1.18	0.99	1.41	0.92
Stroke	1.42	1.06	1.91	1.35	0.99	1.84	0.82
Chronic renal failure	0.59	0.38	0.93	1.21	0.89	1.63	0.01
COPD	1.19	0.84	1.69	1.33	1.01	1.74	0.63
Asthma	0.97	0.65	1.47	1.51	1.15	1.99	0.08
Obesity	0.84	0.53	1.34	1.26	0.95	1.67	0.14
Immunosuppressed	1.92	1.19	3.11	2.87	1.28	6.44	0.40
Cancer	1.94	1.43	2.63	1.60	1.07	2.38	0.44
Hematological malignancy	2.04	1.38	2.99	1.71	0.99	2.95	0.61
Systemic inflammatory disease	1.53	1.14	2.07	1.19	0.90	1.56	0.22
Solid organ transplant recipient	2.89	1.55	5.40	1.66	1.00	2.76	0.18
Hospital type - district: county	0.85	0.73	0.98	0.85	0.73	0.98	NA
Hospital type - university: county	0.84	0.75	0.93	0.84	0.75	0.93	NA

ICU: Intensive care unit, ARDS: Acute respiratory distress syndrome, COVID-19: Corona Virus Disease 2019, HR: Hazard ratio, CI: Confidence interval, NA: Not applicable, SAPS3: Simplified Acute Physiology Score 3 Box III[3], COPD: Chronic obstructive pulmonary disease, P: P-value

SUPPLEMENTARY TABLE S8. A sensitivity analysis, logistic regression model on risk of mortality within 60 days of ICU admission with non-COVID-19 Sepsis or COVID-19. Significance for the interaction with a variable denoting intensive care with non-COVID-19 Sepsis or COVID-19. Sepsis patients also included in the ARDS cohort excluded.

	**Sepsis patients**			**COVID-19 patents**			
	HR	95 % CI		HR	95 % CI		P for interaction
COVID-19 - Sepsis: COVID-19	2.17	1.95	2.41	2.17	1.95	2.41	NA
SAPS3 Box III	2.82	2.61	3.04	2.51	2.22	2.84	0.05
Age	2.22	2.03	2.44	4.35	3.86	4.90	<0.001
Sex - female: male	1.22	1.14	1.30	0.80	0.70	0.90	<0.001
Ischemic heart disease	1.06	0.98	1.16	1.06	0.85	1.32	0.98
Non-ischemic heart disease	1.22	1.13	1.32	1.12	0.93	1.35	0.38
Hypertension	0.98	0.91	1.06	1.00	0.86	1.17	0.83
Type 1 diabetes mellitus	1.09	0.95	1.25	1.70	0.98	2.97	0.13
Type 2 diabetes mellitus	1.09	0.99	1.19	1.17	0.98	1.40	0.46
Stroke	1.10	1.00	1.20	1.36	1.00	1.85	0.19
Chronic renal failure	1.32	1.17	1.50	1.19	0.88	1.60	0.51
COPD	1.30	1.17	1.44	1.34	1.02	1.76	0.82
Asthma	0.92	0.80	1.05	1.47	1.12	1.94	0.003
Obesity	0.94	0.81	1.09	1.25	0.94	1.65	0.08
Immunosuppressed	1.40	1.14	1.72	3.19	1.40	7.28	0.06
Cancer	1.70	1.54	1.88	1.60	1.07	2.38	0.76
Hematological malignancy	1.81	1.53	2.14	1.64	0.94	2.85	0.74
Systemic inflammatory disease	1.09	0.98	1.20	1.18	0.89	1.55	0.60
Solid organ transplant recipient	0.98	0.75	1.26	1.61	0.97	2.68	0.08
Hospital type - district: county	0.84	0.78	0.90	0.84	0.78	0.90	NA
Hospital type - university: county	0.91	0.85	0.97	0.91	0.85	0.97	NA

ICU: Intensive care unit, COVID-19: Corona Virus Disease 2019, ARDS: Acute respiratory distress syndrome, HR: Hazard ratio, CI: Confidence interval, NA: Not applicable, SAPS3: Simplified Acute Physiology Score 3 Box III[3], COPD: Chronic obstructive pulmonary disease, P: P-value

SUPPLEMENTARY TABLE S9. A sensitivity analysis, logistic regression model on risk of mortality within 60 days of ICU admission with non-COVID-19 ARDS or COVID-19. Significance for the interaction with a variable denoting intensive care with non-COVID-19 ARDS or COVID-19. ARDS patients also included in the Sepsis cohort excluded.

	**ARDS patients**			**COVID-19 patents**			
	HR	95 % CI		HR	95 % CI		P for interaction
COVID-19 - ARDS: COVID-19	0.74	0.59	0.91	0.74	0.59	0.91	NA
SAPS3 Box III	1.77	1.42	2.20	2.29	1.93	2.72	0,01
Age	4.10	3.29	5.12	4.98	4.32	5.74	0,06
Sex - female: male	0.77	0.60	0.97	0.79	0.69	0.90	0,84
Ischemic heart disease	0.98	0.70	1.37	1.06	0.85	1.32	0,70
Non-ischemic heart disease	1.01	0.76	1.35	1.11	0.92	1.33	0,62
Hypertension	0.94	0.72	1.23	1.00	0.85	1.17	0,70
Type 1 diabetes mellitus	1.22	0.70	2.12	1.73	0.99	3.01	0,38
Type 2 diabetes mellitus	1.10	0.77	1.58	1.17	0.98	1.40	0,76
Stroke	1.75	1.19	2.58	1.35	0.99	1.84	0,30
Chronic renal failure	0.43	0.24	0.79	1.20	0.89	1.62	0,003
COPD	1.38	0.89	2.14	1.34	1.02	1.75	0,91
Asthma	1.11	0.64	1.94	1.50	1.13	1.97	0,35
Obesity	0.84	0.43	1.63	1.27	0.96	1.68	0,26
Immunosuppressed	2.70	1.39	5.24	3.17	1.39	7.23	0,76
Cancer	2.07	1.40	3.06	1.59	1.07	2.37	0,36
Hematological malignancy	1.77	1.05	2.99	1.66	0.95	2.90	0,86
Systemic inflammatory disease	1.88	1.27	2.78	1.17	0.89	1.55	0,05
Solid organ transplant recipient	4.30	1.85	9.97	1.68	1.01	2.79	0,06
Hospital type - district: county	0.84	0.72	0.98	0.84	0.72	0.98	NA
Hospital type - university: county	0.82	0.73	0.92	0.82	0.73	0.92	NA

ICU: Intensive care unit, ARDS: Acute respiratory distress syndrome, COVID-19: Corona Virus Disease 2019, HR: Hazard ratio, CI: Confidence interval, NA: Not applicable, SAPS3: Simplified Acute Physiology Score 3 Box III[3], COPD: Chronic obstructive pulmonary disease, P: P-value

SUPPLEMENTARY TABLE S10. A sensitivity analysis, Binary logistic regression model on risk of mortality within 60 days of ICU admission with Sepsis or COVID-19. Significance for the interaction with a variable denoting intensive care with non-COVID-19 Sepsis or COVID-19. SAPS3 Box III was excluded from the model.

	Sepsis patients			COVID-19 patents			
	OR	95 % CI		OR	95 % CI		P for interaction
COVID-19 - Sepsis: COVID-19	1.12	1.02	1.23	1.12	1.02	1.23	NA
Age	2.02	1.86	2.20	4.25	3.79	4.78	<0.001
Sex - female: male	1.13	1.06	1.20	0.82	0.72	0.93	<0.001
Ischemic heart disease	1.00	0.93	1.09	1.07	0.87	1.32	0.57
Non-ischemic heart disease	1.18	1.10	1.27	1.15	0.96	1.38	0.80
Hypertension	0.97	0.90	1.03	1.01	0.87	1.18	0.60
Type 1 diabetes mellitus	1.04	0.92	1.19	1.71	0.99	2.96	0.08
Type 2 diabetes mellitus	1.09	1.01	1.18	1.18	1.00	1.40	0.41
Stroke	1.07	0.98	1.17	1.40	1.04	1.88	0.09
Chronic renal failure	1.49	1.32	1.67	1.44	1.08	1.92	0.84
COPD	1.27	1.16	1.40	1.38	1.06	1.80	0.55
Asthma	0.87	0.77	0.99	1.39	1.06	1.82	0.002
Obesity	0.89	0.77	1.01	1.26	0.95	1.65	0.03
Immunosuppressed	1.45	1.20	1.75	2.77	1.23	6.24	0.13
Cancer	1.44	1.31	1.58	1.43	0.97	2.11	0.98
Hematological malignancy	2.10	1.81	2.44	1.97	1.16	3.34	0.82
Systemic inflammatory disease	1.07	0.98	1.18	1.23	0.94	1.61	0.34
Solid organ transplant recipient	1.01	0.80	1.27	1.57	0.96	2.58	0.12
Hospital type - district: county	0.76	0.71	0.81	0.76	0.71	0.81	NA
Hospital type - university: county	0.98	0.92	1.04	0.98	0.92	1.04	NA

ICU: Intensive care unit, COVID-19: Corona Virus Disease 2019, SAPS3: Simplified Acute Physiology Score 3 Box III[3], HR: Hazard ratio, CI: Confidence interval, NA: Not applicable, COPD: Chronic obstructive pulmonary disease, P: P-value

SUPPLEMENTARY TABLE S11. A sensitivity analysis, Binary logistic regression model on risk of mortality within 60 days of ICU admission with ARDS or COVID-19. Significance for the interaction with a variable denoting intensive care with non-COVID-19 ARDS or COVID-19. SAPS3 Box III was excluded from the model.

	**Sepsis patients**			**COVID-19 patents**			
	OR	95 % CI		OR	95 % CI		P for interaction
COVID-19 - Sepsis: COVID-19	0.49	0.42	0.58	0.49	0.42	0.58	NA
Age	3.41	2.85	4.09	4.85	4.25	5.54	<0,001
Sex - female: male	0.88	0.74	1.05	0.80	0.71	0.91	0,41
Ischemic heart disease	1.08	0.84	1.40	1.06	0.86	1.32	0,92
Non-ischemic heart disease	1.10	0.88	1.36	1.14	0.95	1.37	0,79
Hypertension	0.84	0.68	1.02	1.01	0.87	1.18	0,15
Type 1 diabetes mellitus	1.20	0.80	1.80	1.70	0.99	2.94	0,31
Type 2 diabetes mellitus	1.15	0.88	1.50	1.19	1.00	1.42	0,82
Stroke	1.38	1.03	1.83	1.39	1.03	1.88	0,95
Chronic renal failure	0.66	0.43	1.02	1.44	1.07	1.92	0,004
COPD	1.16	0.82	1.63	1.38	1.06	1.79	0,43
Asthma	0.98	0.65	1.46	1.42	1.08	1.86	0,13
Obesity	0.80	0.51	1.27	1.30	0.99	1.71	0,08
Immunosuppressed	1.95	1.21	3.12	2.77	1.24	6.21	0,46
Cancer	1.87	1.38	2.52	1.42	0.96	2.10	0,28
Hematological malignancy	2.18	1.49	3.19	2.03	1.19	3.46	0,83
Systemic inflammatory disease	1.44	1.07	1.93	1.23	0.94	1.60	0,43
Solid organ transplant recipient	2.74	1.48	5.06	1.66	1.01	2.73	0,21
Hospital type - district: county	0.83	0.73	0.96	0.83	0.73	0.96	NA
Hospital type - university: county	0.83	0.75	0.92	0.83	0.75	0.92	NA

ICU: Intensive care unit, ARDS: Acute respiratory distress syndrome, COVID-19: Corona Virus Disease 2019, HR: Hazard ratio, CI: Confidence interval, NA: Not applicable, COPD: Chronic obstructive pulmonary disease, P: P-value

SUPPLEMENTARY TABLE S12. A sensitivity analysis, Binary logistic model on risk of mortality within 60 days of ICU admission with ARDS or COVID-19. Significance for the interaction with a variable denoting intensive care with non-COVID-19 ARDS or COVID-19. 14 overly influential observations were removed.

	Sepsis patients			COVID-19 patents			
	HR	95 % CI		HR	95 % CI		P for interaction
COVID-19 - ARDS: COVID-19	0.74	0.63	0.88	0.74	0.63	0.88	NA
SAPS3 Box III	1.59	1.33	1.90	2.27	1.94	2.67	<0.001
Age	3.41	2.85	4.07	4.57	4.00	5.21	<0.001
Sex - female: male	0.91	0.76	1.09	0.79	0.69	0.90	0.21
Ischemic heart disease	1.08	0.83	1.40	1.05	0.85	1.31	0.91
Non-ischemic heart disease	1.15	0.92	1.44	1.10	0.91	1.33	0.75
Hypertension	0.85	0.69	1.04	1.00	0.86	1.17	0.20
Type 1 diabetes mellitus	1.17	0.77	1.78	1.60	0.91	2.81	0.38
Type 2 diabetes mellitus	1.18	0.90	1.55	1.17	0.98	1.40	0.96
Stroke	1.42	1.06	1.90	1.35	0.99	1.84	0.82
Chronic renal failure	0.57	0.36	0.89	1.24	0.92	1.67	0.005
COPD	1.21	0.85	1.72	1.37	1.04	1.80	0.57
Asthma	0.88	0.58	1.34	1.51	1.15	2.00	0.03
Obesity	0.80	0.50	1.28	1.24	0.93	1.65	0.12
Immunosuppressed	1.98	1.22	3.21	4.56	1.79	11.61	0.12
Cancer	1.93	1.42	2.63	1.62	1.08	2.42	0.49
Hematological malignancy	2.06	1.40	3.03	1.62	0.92	2.87	0.50
Systemic inflammatory disease	1.58	1.17	2.15	1.16	0.88	1.53	0.13
Solid organ transplant recipient	3.35	1.73	6.47	1.66	1.00	2.77	0.10
Hospital type - district: county	0.84	0.73	0.97	0.84	0.73	0.97	NA
Hospital type - university: county	0.83	0.75	0.92	0.83	0.75	0.92	NA

ICU: Intensive care unit, COVID-19: Corona Virus Disease 2019, ARDS: Acute respiratory distress syndrome, HR: Hazard ratio, CI: Confidence interval, NA: Not applicable, SAPS3: Simplified Acute Physiology Score 3 Box III[3] COPD: Chronic obstructive pulmonary disease, P: P-value

SUPPLEMENTARY TABLE S13. A sensitivity analysis, Binary logistic regression model on risk of mortality within 60 days of ICU admission with Sepsis or COVID-19. Significance for the interaction with a variable denoting intensive care with non-COVID-19 Sepsis or COVID-19. Hospital type was not included in the model.

	**Sepsis patients**			**COVID-19 patents**			
	OR	95 % CI		OR	95 % CI		P for interaction
COVID-19 - Sepsis: COVID-19	2.06	1.85	2.28	2.06	1.85	2.28	NA
SAPS3 Box III	2.76	2.56	2.97	2.50	2.21	2.83	0.09
Age	2.21	2.02	2.41	4.34	3.86	4.89	<0.001
Sex - female: male	1.21	1.14	1.29	0.79	0.70	0.90	<0.001
Ischemic heart disease	1.07	0.98	1.16	1.06	0.85	1.32	0.97
Non-ischemic heart disease	1.22	1.13	1.31	1.12	0.93	1.35	0.41
Hypertension	0.96	0.90	1.03	1.00	0.86	1.17	0.66
Type 1 diabetes mellitus	1.07	0.94	1.23	1.70	0.97	2.97	0.12
Type 2 diabetes mellitus	1.09	0.99	1.18	1.17	0.98	1.39	0.46
Stroke	1.09	1.00	1.20	1.35	0.99	1.84	0.19
Chronic renal failure	1.29	1.14	1.46	1.19	0.89	1.61	0.63
COPD	1.26	1.14	1.39	1.34	1.02	1.76	0.68
Asthma	0.91	0.79	1.04	1.47	1.11	1.93	0.002
Obesity	0.93	0.81	1.07	1.24	0.93	1.64	0.08
Immunosuppressed	1.37	1.13	1.67	3.16	1.38	7.21	0.05
Cancer	1.67	1.52	1.85	1.59	1.06	2.36	0.80
Hematological malignancy	1.85	1.58	2.17	1.65	0.95	2.88	0.69
Systemic inflammatory disease	1.09	0.99	1.20	1.18	0.89	1.55	0.61
Solid organ transplant recipient	1.02	0.80	1.31	1.61	0.97	2.67	0.12

ICU: Intensive care unit, COVID-19: Corona Virus Disease 2019, HR: Hazard ratio, CI: Confidence interval, NA: Not applicable, SAPS3: Simplified Acute Physiology Score 3 Box III[3], COPD: Chronic obstructive pulmonary disease, P: P-value

SUPPLEMENTARY TABLE S14. A sensitivity analysis, Binary logistic regression model on risk of mortality within 60 days of ICU admission with ARDS or COVID-19. Significance for the interaction with a variable denoting intensive care with non-COVID-19 ARDS or COVID-19. Hospital type was not included in the model.

	**ARDS patients**			**COVID-19 patents**			
	OR	95 % CI		OR	95 % CI		P for interaction
COVID-19 - Sepsis: COVID-19	0.75	0.63	0.89	0.75	0.63	0.89	NA
SAPS3 Box III	1.57	1.31	1.87	2.27	1.94	2.66	<0,001
Age	3.63	3.02	4.36	4.86	4.24	5.56	<0,001
Sex - female: male	0.91	0.76	1.09	0.79	0.69	0.90	0,20
Ischemic heart disease	1.05	0.81	1.37	1.06	0.85	1.32	0,98
Non-ischemic heart disease	1.14	0.91	1.42	1.11	0.92	1.34	0,85
Hypertension	0.84	0.69	1.04	1.00	0.86	1.17	0,20
Type 1 diabetes mellitus	1.21	0.79	1.84	1.72	0.99	3.00	0,32
Type 2 diabetes mellitus	1.18	0.90	1.55	1.17	0.98	1.40	0,96
Stroke	1.42	1.06	1.90	1.35	0.99	1.84	0,81
Chronic renal failure	0.58	0.37	0.91	1.21	0.90	1.63	0,01
COPD	1.18	0.83	1.67	1.34	1.02	1.76	0,58
Asthma	0.95	0.63	1.43	1.48	1.12	1.95	0,08
Obesity	0.84	0.53	1.34	1.24	0.94	1.65	0,16
Immunosuppressed	1.91	1.18	3.09	3.09	1.36	7.04	0,32
Cancer	1.90	1.40	2.58	1.58	1.06	2.35	0,47
Hematological malignancy	2.07	1.41	3.04	1.67	0.95	2.91	0,53
Systemic inflammatory disease	1.52	1.13	2.06	1.18	0.89	1.55	0,21
Solid organ transplant recipient	3.12	1.65	5.87	1.65	0.99	2.74	0,12

ICU: Intensive care unit, ARDS: Acute Respiratory distress Syndrome, COVID-19: Corona Virus Disease 2019, OR: Odds ratio, CI: Confidence interval, NA: Not applicable, SAPS3: Simplified Acute Physiology Score 3 Box III[3] , COPD: Chronic obstructive pulmonary disease, P: P-value

SUPPLEMENTARY TABLE S15. A sensitivity analysis. Linear regression on age as a predictor of ICU length of stay in COVID-19 and Sepsis patients who died in ICU. An interaction with cohort, COVID-19 or non-COVID-19 Sepsis, was added to asses cohort influence on the predictive performance of age.

	**COVID-19 patients**			**Sepsis patients**			
	Effect	95% CI		Effect	95% CI		P for interaction
Age	-7.42	-9.06	-5.78	-1.11	-2.06	-0.15	<0.001
Cohort	9.35	8.03	10.7	9.35	8.03	10.7	NA

ICU: intensive care unit, COVID-19: Coronavirus disease 2019, CI: Confidence interval, P: P-value for interaction with cohort, NA: Not applicable

References

1 Kaukonen, K. M., Bailey, M., Suzuki, S., Pilcher, D. & Bellomo, R. Mortality related to severe sepsis and septic shock among critically ill patients in Australia and New Zealand, 2000-2012. *JAMA* **311**, 1308-1316, doi:10.1001/jama.2014.2637 (2014).

2 Strandberg, G., Walther, S., Agvald Öhman, C. & Lipcsey, M. Mortality after Severe Sepsis and Septic Shock in Swedish Intensive Care Units 2008-2016-A nationwide observational study. *Acta Anaesthesiol Scand* **64**, 967-975, doi:10.1111/aas.13587 (2020).

3 Moreno, R. P. *et al.* SAPS 3--From evaluation of the patient to evaluation of the intensive care unit. Part 2: Development of a prognostic model for hospital mortality at ICU admission. *Intensive Care Med* **31**, 1345-1355, doi:10.1007/s00134-005-2763-5 (2005).

4 Quan, H. *et al.* Updating and validating the Charlson comorbidity index and score for risk adjustment in hospital discharge abstracts using data from 6 countries. *Am J Epidemiol* **173**, 676-682, doi:10.1093/aje/kwq433 (2011).

Model code R

Imputation

```
RAW <- readr::read_csv2('Z:/C_S_A.csv')

attr(RAW, "spec") <- NULL

foo <- function(x){

  n <- length(unique(x))

  if(n <= 500 & is.character(x)) factor(x) else x

}

X <- RAW

X[] <- lapply(RAW, foo)

X$CCI_fix <- as.factor(X$CCI_fix)

saveRDS(X, file = "Z:/C_S_A_imp.Rds")

## ## imputering ----------------------------------------------------------

pm <- matrix(1, nrow = ncol(X), ncol = ncol(X))

rownames(pm) <- colnames(pm) <- names(X)

diag(pm) <- 0

pm["ID", ] <- 0

pm[, "ID"] <- 0

pm["CovSeps", ] <- 0

pm[, "CovSeps"] <- 0

pm["CovArds", ] <- 0

pm[, "CovArds"] <- 0

pm["CovSeps2", ] <- 0

pm[, "CovSeps2"] <- 0

pm["CovArds2", ] <- 0

pm[, "CovArds2"] <- 0

pm["Overlap", ] <- 0

pm[, "Overlap"] <- 0

pm["TimeAtRisk_ICU", ] <- 0

pm[, "TimeAtRisk_ICU"] <- 0
```

```
pm["DeadICU_n", ] <- 0

pm[, "DeadICU_n"] <- 0

pm["COVID", ] <- 0

pm[, "COVID"] <- 0

pm["Sepsis", ] <- 0

pm[, "Sepsis"] <- 0

pm["ARDS", ] <- 0

pm[, "ARDS"] <- 0

pm["CCI_fix", ] <- 0

pm[, "CCI_fix"] <- 0

pm["Time", ] <- 0

pm[, "Time"] <- 0

pm["CSA", ] <- 0

pm[, "CSA"] <- 0


IMP <- mice(data = X, m = 5, predictorMatrix = pm,

      method = c("", "", "", "", "", "", "", "", "", "",

            "", "", "", "pmm", "pmm", "pmm", "pmm", "pmm", "pmm", "pmm",

            "pmm", "pmm", "pmm", "pmm", "pmm", "pmm", "pmm", "pmm", "pmm", "pmm",

            "pmm", "pmm", "pmm", "pmm", "pmm", "","pmm"), seed = 20201117)

saveRDS(IMP, "Z:/Imputerad_C_S_A_imp.Rds")
```

60-day mortality main model

```
RAW <- readr::read_csv2('Z:/C_S_A.csv')

str(RAW)

attr(RAW, "spec") <- NULL

foo <- function(x){

  n <- length(unique(x))

  if(n <= 500 & is.character(x)) factor(x) else x

}

X <- RAW
```

```
X[] <- lapply(RAW, foo)

library('rms')

attach(X)

dd <-
datadist(Dead60_n,SAPS3_Box3,Age,Sex,CovSeps,CovArds,IHD,OtherHD,Hypertens,Diabetes1,Diabetes2,Stroke
,Njursvikt,KOL,Astma,Fetma,Immunsupprimerad,Cancer_dia,BlodCancer,Inflam, Transplanterad,SjukhusTyp)

options(datadist='dd')


f <- lrm(Dead60_n ~ SjukhusTyp + rcs(SAPS3_Box3,4) + CovSeps + SAPS3_Box3 %ia% CovSeps + rcs(Age,4) + Age
%ia% CovSeps + Sex*CovSeps + IHD*CovSeps + OtherHD*CovSeps + Hypertens*CovSeps + Diabetes1*CovSeps
+ Diabetes2*CovSeps + Stroke*CovSeps + Njursvikt*CovSeps + KOL*CovSeps + Astma*CovSeps +
Fetma*CovSeps + Immunsupprimerad*CovSeps + Cancer_dia*CovSeps + BlodCancer*CovSeps +
Inflam*CovSeps + Transplanterad*CovSeps, x=TRUE, y=TRUE, data=X)

#CovSeps = 1 -> Covid

summary(f,CovSeps=0)

summary(f,CovSeps=1)

anova(f)

f



#Covid - ARDS

g <- lrm(Dead60_n ~ SjukhusTyp + rcs(SAPS3_Box3,4) + CovArds + SAPS3_Box3 %ia% CovArds + rcs(Age,4) + Age
%ia% CovArds + Sex*CovArds + IHD*CovArds + OtherHD*CovArds + Hypertens*CovArds + Diabetes1*CovArds +
Diabetes2*CovArds + Stroke*CovArds + Njursvikt*CovArds + KOL*CovArds + Astma*CovArds + Fetma*CovArds
+ Immunsupprimerad*CovArds + Cancer_dia*CovArds + BlodCancer*CovArds + Inflam*CovArds +
Transplanterad*CovArds, x=TRUE, y=TRUE, data=X)

#CovArds = 1 -> Covid

tabout(summary(g,CovArds=0))

tabout(summary(g,CovArds=1))

tabout(anova(g))

g

#Validation of model assumptions

#Influential observations - no influential observations found in CovSeps.
```

```
ii <- which.influence(f, cutoff=.2)

ddd <- data.frame(Age, SAPS3_Box3, Sex, SjukhusTyp, IHD, OtherHD, Hypertens, Diabetes1, Diabetes2, Stroke, Njursvikt, KOL, Astma, Fetma, Immunsupprimerad, Cancer_dia, BlodCancer, Inflam, Transplanterad, CovSeps, Dead60_n)

show.influence(ii, ddd)

#14 influential observations in COv ARDS.

jj <- which.influence(g, cutoff=.2)

ppp <- data.frame(Age, SAPS3_Box3, Sex, SjukhusTyp, IHD, OtherHD, Hypertens, Diabetes1, Diabetes2, Stroke, Njursvikt, KOL, Astma, Fetma, Immunsupprimerad, Cancer_dia, BlodCancer, Inflam, Transplanterad, CovArds, Dead60_n)

show.influence(jj, ppp)


#Sensitivity analysis without cases with influential observations.

X <- X[-c(781, 1246, 1411, 2409, 2815, 3073, 3768, 4707, 6364, 7809, 29900, 29903, 30647, 31512), ]

detach(X)

attach(X)

str(X)

dd <- datadist(Dead60_n,SAPS3_Box3,Age,Sex,CovSeps,CovArds,IHD,OtherHD,Hypertens,Diabetes1,Diabetes2,Stroke, Njursvikt, KOL, Astma, Fetma, Immunsupprimerad, Cancer_dia, BlodCancer, Inflam, Transplanterad,SjukhusTyp)

options(datadist='dd')


h <- lrm(Dead60_n ~ SjukhusTyp + rcs(SAPS3_Box3,4) + CovArds + SAPS3_Box3 %ia% CovArds + rcs(Age,4) + Age %ia% CovArds + Sex*CovArds + IHD*CovArds + OtherHD*CovArds + Hypertens*CovArds + Diabetes1*CovArds + Diabetes2*CovArds + Stroke*CovArds + Njursvikt*CovArds + KOL*CovArds + Astma*CovArds + Fetma*CovArds + Immunsupprimerad*CovArds + Cancer_dia*CovArds + BlodCancer*CovArds + Inflam*CovArds + Transplanterad*CovArds, x=TRUE, y=TRUE, data=X)

summary(h,CovArds=0)

summary(h,CovArds=1)

anova(h)

h

#Multicolinearity to high vif in age and SAPS3.
```

vif(f)

vif(g)

Data management SPSS

```
* Encoding: UTF-8.

*COVID skapas i Skapa_COVIDrev.sps.

DATASET CLOSE all.

*Montera SIR_data_05_17_fr_Gunnar_ickeBin på X.


cd 'Z:/'.

*Obs filen ligger på X!.

*GET DATA

  /TYPE=XLSX

  /FILE='X:\SIR-data_05_17_fr_Gunanr_ickeBin.xlsx'

  /SHEET=name 'SIR-data'

  /CELLRANGE=FULL

  /READNAMES=ON

  /TRAILINGSPACES IGNORE=YES

  /DATATYPEMIN PERCENTAGE=95.0

  /HIDDEN IGNORE=YES.

*EXECUTE.

*DATASET NAME SIR WINDOW=FRONT.


SAVE OUTFILE 'SIR_start.sav'.


DATASET CLOSE all.

GET FILE 'SIR_start.sav'.

DATASET NAME SIR WINDOW=FRONT.

DATASET ACTIVATE SIR.


RENAME VARIABLES (LÃ¶pNr_Pnr LÃ¶pNr_VTF AvlidenPÃ¥IVA = ID LpnrVTF Avliden_IVA).


FORMATS InskrivDatum(date11).
```

```
   FORMATS UtskrivDatum(date11).

   FORMATS AvregDatum(date11).

   FORMATS ID(f10).

   FORMATS LpnrVTF(f10).

   FORMATS Inskr_Ar(f4).

   FORMATS Alder(f4).

   FORMATS CancerTerapi(f2).

     EXECUTE.



*Slå ihop alla ICD-variabler.

STRING IVA_diagnos(A230).

Compute IVA_Diagnos = concat(rtrim(@1ICD10),' ',rtrim(@2ICD10),' ',rtrim(@3ICD10),' ',rtrim(@4ICD10),' ',rtrim(@5ICD10),' ',rtrim(@6ICD10),' ',rtrim(@7ICD10),

' ',rtrim(@8ICD10),' ',rtrim(@9ICD10),' ',rtrim(@10ICD10),' ',rtrim(@11ICD10),' ',rtrim(@12ICD10),' ',rtrim(@13ICD10),' ',rtrim(@14ICD10),' ',rtrim(@15ICD10),' ',rtrim(@16ICD10),

' ',rtrim(@17ICD10),' ',rtrim(@18ICD10),' ',rtrim(@19ICD10),' ',rtrim(@20ICD10),' ',rtrim(@21ICD10),' ',rtrim(@22ICD10),' ',rtrim(@23ICD10),' ',rtrim(@24ICD10),' ',rtrim(@25ICD10),

' ',rtrim(@26ICD10),' ',rtrim(@27ICD10),' ',rtrim(@28ICD10)).

EXECUTE.


*Undersök bredden (221).

compute count_IVA_Diagnos=char.length(IVA_Diagnos).

EXECUTE.

FREQUENCIES VARIABLES=count_IVA_Diagnos

  /STATISTICS=MINIMUM MAXIMUM MEAN

  /ORDER=ANALYSIS.

DELETE VARIABLES count_IVA_Diagnos @1ICD10 to @28ICD10.

   EXECUTE.
```

```
*Skapa SAPS3 utan ålder och comorb..

NUMERIC SAPS3_TEMP(f4).

   RECODE Alder  (1 thru 39 = 0) (40 thru 59 = 3) (60 thru 69 = 9) (70 thru 74 = 13) (75 thru 79 = 15) (80 thru hi = 18) INTO SAPS3_TEMP.

   EXECUTE.

   IF CancerTerapi = 2  SAPS3_TEMP =  SAPS3_TEMP + 3.

   IF KronHjartsvikt = 2 SAPS3_TEMP =  SAPS3_TEMP + 6.

   IF Blodmalignitet = 2 SAPS3_TEMP =  SAPS3_TEMP + 6.

   IF Cirrhos = 2 SAPS3_TEMP =  SAPS3_TEMP + 9.

   IF AIDS  = 2 SAPS3_TEMP =  SAPS3_TEMP + 9.

   IF Cancer = 2 SAPS3_TEMP =  SAPS3_TEMP + 9.

NUMERIC SAPS3_u_comorb(f4).

   COMPUTE  SAPS3_u_comorb = SAPS3Score - SAPS3_TEMP.

   EXECUTE.



SORT CASES LpnrVTF(a).

*Lägg till Inskrivnings TID.

*Hämta Tider_ML_krypt från servern. Lägg i Z. Ta bort skrivskydd.

GET file 'Y:\Original_SPSS\KonsekvenserAvKritiskSjukdom_ML_Tider.sav'.

DATASET NAME Tider WINDOW=FRONT.

DATASET ACTIVATE Tider.

DELETE VARIABLES L�pNr_Pnr.

   EXECUTE.

RENAME VARIABLES (L�pNr_VTF = LpnrVTF).

   EXECUTE.

   FORMATS LpnrVTF(f10).


SORT CASES LpnrVTF(a).
```

```
DATASET ACTIVATE SIR.

MATCH FILES /FILE=*

  /FILE='Tider'

  /BY LpnrVTF.

        EXECUTE.

    DATASET CLOSE Tider.

    DATASET ACTIVATE SIR.


*Lägg till ventilatorbehandling.

Get file 'Y:\Original_SPSS\Ventilator.sav'.

DATASET NAME Vent.

DATASET ACTIVATE Vent.

    RENAME VARIABLES (L�pNr_VTF CRRT AtgardTidMinuter= LpnrVTF IMV IMV_tid).

        EXECUTE.

    FORMATS LpnrVTF(f10).

  SORT CASES LpnrVTF(a) StartTidpunkt(a).

    NUMERIC Counter(f3).

    IF $casenum =1 or LpnrVTF ne lag(LpnrVTF) Counter = 1.

    IF LpnrVTF = lag(LpnrVTF) Counter = lag(Counter) + 1.

        EXECUTE.


*Lägg ihop mot rad 1 per vtf.

SORT CASES LpnrVTF(a) Counter(d).

IF LpnrVTF = lag(LpnrVTF) IMV_tid = IMV_tid + lag(IMV_tid).

       EXECUTE.

SELECT IF Counter = 1.

    EXECUTE.
```

```
ADD FILES FILE*
   /KEEP LpnrVTF IMV_tid.
   EXECUTE.

SORT CASES LpnrVTF(a).

DATASET ACTIVATE SIR.
MATCH FILES /FILE=*
 /FILE='Vent'
 /BY LpnrVTF.
      EXECUTE.
   DATASET CLOSE Vent.
   DATASET ACTIVATE SIR.

*Lägg till dialysbehandling.
Get file 'Y:\Original_SPSS\CRRT.sav'.
DATASET NAME C.
DATASET ACTIVATE C.
   RENAME VARIABLES (L�pNr_VTF AtgardTidMinuter = LpnrVTF CRRT_tid).
      EXECUTE.
VARIABLE LABELS CRRT_tid 'CRRT_tid i minuter'.
   EXECUTE.
   FORMATS LpnrVTF(f10).

 SORT CASES LpnrVTF(a) StartTidpunkt(a).
   NUMERIC Counter(f3).
   IF $casenum =1 or LpnrVTF ne lag(LpnrVTF) Counter = 1.
   IF LpnrVTF = lag(LpnrVTF) Counter = lag(Counter) + 1.
      EXECUTE.
```

```
*Lägg ihop mot rad 1 per vtf.
SORT CASES LpnrVTF(a) Counter(d).
IF LpnrVTF = lag(LpnrVTF) CRRT_tid = CRRT_tid + lag(CRRT_tid).
      EXECUTE.
SELECT IF Counter = 1.
   EXECUTE.
ADD FILES FILE*
   /KEEP LpnrVTF CRRT_tid.
   EXECUTE.
SORT CASES LpnrVTF(a).

DATASET ACTIVATE SIR.
MATCH FILES /FILE=*
  /FILE='C'
  /BY LpnrVTF.
       EXECUTE.
   DATASET CLOSE C.
   DATASET ACTIVATE SIR.

*Ta bort OptOutade 3 st.
DESCRIPTIVES ID.
SELECT IF InskrTid > 0.
   EXECUTE.
DESCRIPTIVES ID.
SELECT IF InskrivDatum > 0.
   EXECUTE.
DESCRIPTIVES ID.
```

```
DELETE VARIABLES InskrivDatum UtskrivDatum.
   EXECUTE.
compute day = xdate.mday(InskrTid).
compute month = xdate.month(InskrTid).
compute year = xdate.year(InskrTid).
compute InskrivDatum = date.dmy(day,month,year).
formats InskrivDatum(date11).
   EXECUTE.
compute day = xdate.mday(UtskrTid).
compute month = xdate.month(UtskrTid).
compute year = xdate.year(UtskrTid).
compute UtskrivDatum = date.dmy(day,month,year).
formats UtskrivDatum(date11).
   EXECUTE.



SORT CASES ID(a) InskrTid(a) Utskrtid(a).
NUMERIC CounterIVA(f3).
   IF $casenum = 1 or ID ne lag(ID) CounterIVA = 1.
   IF ID = lag(ID) CounterIVA = lag(CounterIVA) + 1.
   EXECUTE.
FREQUENCIES CounterIVA Inskr_Ar.

*Ta bort vårdtillfällen före 2011.
SELECT IF (InskrivDatum > DATE.DMY(31,12,2010)).
   EXECUTE.

DELETE VARIABLES CounterIVA.
```

```
   EXECUTE.
SORT CASES ID(a) InskrTid(a) Utskrtid(a).
NUMERIC CounterIVA(f3).
   IF $casenum = 1 or ID ne lag(ID) CounterIVA = 1.
   IF ID = lag(ID) CounterIVA = lag(CounterIVA) + 1.
   EXECUTE.
FREQUENCIES CounterIVA.


*Ta bort vårdtillfällen efter 2016-12-31.
SELECT IF (InskrivDatum < DATE.DMY(01,01,2017)).
   EXECUTE.
DELETE VARIABLES CounterIVA.
   EXECUTE.
SORT CASES ID(a) InskrTid(a) Utskrtid(a).
NUMERIC CounterIVA(f3).
   IF $casenum = 1 or ID ne lag(ID) CounterIVA = 1.
   IF ID = lag(ID) CounterIVA = lag(CounterIVA) + 1.
   EXECUTE.
FREQUENCIES CounterIVA.


ADD FILES FILE*
   /KEEP LpnrVTF ID InskrTid UtskrTid InskrivDatum UtskrivDatum Avliden AvregDatum Avliden_IVA SjukhusTyp
Alder Kon Opererad
   SAPS3Score SAPS3ScoreBoxIII Operationstyp APACHE_Score APACHE_AkutFysPoang Status
   IVA_diagnos SAPS3_TEMP SAPS3_u_comorb IMV_tid CRRT_tid CounterIVA.
      EXECUTE.
*Sepsis.
   COMPUTE BB = (CHAR.INDEX(UPCASE(IVA_diagnos),'R651') > 0).
   COMPUTE CC = (CHAR.INDEX(UPCASE(IVA_diagnos),'R572') > 0).
   COMPUTE AA = (CHAR.INDEX(UPCASE(IVA_diagnos),'A419') > 0).
```

```
    EXECUTE.
NUMERIC Sepsis_grad(f2).
  VALUE LABELS Sepsis_grad 0 'Ingen sepsis' 1 'Severe sepsis' 2 'Septic shock' 5 'Unspecified Sepsis'.
  IF AA > 0 Sepsis_grad = 5.
  IF BB > 0 Sepsis_grad = 1.
  IF CC > 0 Sepsis_grad = 2.
  IF sysmis(Sepsis_grad) Sepsis_grad = 0.
    EXECUTE.

NUMERIC Sepsis(f2).
  IF AA > 0 or BB > 0 or CC> 0 Sepsis = 1.
  IF sysmis(Sepsis) Sepsis = 0.
    EXECUTE.
FREQUENCIES Sepsis.

  DELETE VARIABLES AA BB CC.
    EXECUTE.

*ARDS.
NUMERIC ARDS(f2).
  VARIABLE LABELS ARDS 'ARDS'.
  COMPUTE ARDS = (CHAR.INDEX(UPCASE(IVA_diagnos),'J809') > 0).
  IF sysmis(ARDS) ARDS = 0.
    EXECUTE.
FREQUENCIES ARDS.
*Markera sepsisvårdtillfälle + alla efterföljande.
NUMERIC S(f2).
  VARIABLE LABELS S 'vårdtillfälle med sepsis eller efter sepsisvårdtillfälle'.
If Sepsis = 1 S = 1.
```

```
IF ID = lag(ID) and lag(S) = 1 S = 1.


*Markera ARDS-vårdtillfälle + alla efterföljande.
NUMERIC A(f2).
   VARIABLE LABELS A 'vårdtillfälle med ARDS eller efter ARDS-vårdtillfälle'.
If ARDS = 1 A = 1.
IF ID = lag(ID) and lag(A) = 1 A = 1.
   EXECUTE.



SAVE OUTFILE 'SIR_temp2.sav'.
   DATASET CLOSE all.
GET FILE 'SIR_temp2.sav'.
DATASET NAME Sepsis WINDOW=FRONT.
DATASET ACTIVATE Sepsis.


DELETE VARIABLES CounterIVA.
   EXECUTE.
SORT CASES ID(a) InskrTid(a) Utskrtid(a).
NUMERIC CounterIVA(f3).
   IF $casenum = 1 or ID ne lag(ID) CounterIVA = 1.
   IF ID = lag(ID) CounterIVA = lag(CounterIVA) + 1.
   EXECUTE.
FREQUENCIES CounterIVA.


*SEPSIS!. Sortera bort IVA före sepsis och ej sepsis.
SELECT IF S = 1.
EXECUTE.
DELETE VARIABLES CounterIVA.
```

```
   EXECUTE.
SORT CASES ID(a) InskrTid(a) Utskrtid(a).
NUMERIC CounterIVA(f3).
   IF $casenum = 1 or ID ne lag(ID) CounterIVA = 1.
   IF ID = lag(ID) CounterIVA = lag(CounterIVA) + 1.
   EXECUTE.
FREQUENCIES CounterIVA.
*diff är dagar mellan intensivvårdstillfällena. 0 eller 1 dags skillnad innebär samma vtf.
Compute diff = datediff(InskrTid,lag(UtskrTid),'hours').
Compute diff = Diff / 24.
   VARIABLE LABELS diff ' days from out previous to in actual ICU episode'.
   IF ID ne lag(ID) diff = $sysmis.
   IF CounterIVA = 1 X = 1.
   IF lag(X) = 1 and diff < 1 X = 1.
      EXECUTE.
SELECT IF X = 1.
   EXECUTE.
FREQUENCIES CounterIVA.


*Skapa och spara ett vtf.
DELETE VARIABLES CounterIVA.
   EXECUTE.
SORT CASES ID(a) InskrTid(a) UtskrTid(a).
NUMERIC CounterIVA(f3).
   IF $casenum = 1 or ID ne lag(ID) CounterIVA = 1.
   IF ID = lag(ID) CounterIVA = lag(CounterIVA) + 1.
   EXECUTE.
*Åtgärder.
```

*Lägg ihop mot första vårdtillfället.

```
SORT CASES ID(a) CounterIVA(d).
   EXECUTE.
IF ID = lag(ID) CRRT_tid = CRRT_tid + lag(CRRT_tid).
IF ID = lag(ID) IMV_tid = IMV_tid + lag(IMV_tid).
IF ID = lag(ID) and lag(Avliden_IVA) = 'Ja' Avliden_IVA = 'Ja'.
IF ID = lag(ID) UtskrivDatum = lag(UtskrivDatum).
IF ID = lag(ID) UtskrTid = lag(UtskrTid).
IF ID = lag(ID) and lag(Sepsis_grad) = 1 Sepsis_grad = 1.
IF ID = lag(ID) and lag(Sepsis_grad) = 2 Sepsis_grad = 2.
IF ID = lag(ID) and lag(Sepsis_grad) = 5 Sepsis_grad = 5.
      EXECUTE.
SELECT IF CounterIVA = 1.
   EXECUTE.
DESCRIPTIVES ID.

NUMERIC IMV(f2).
   VALUE LABELS IMV 1 'IMV' 0 'No IMV'.
NUMERIC CRRT(f2).
   VALUE LABELS CRRT 1 'CRRT' 0 'No CRRT'.
   IF IMV_tid > 0 IMV = 1.
   IF sysmis(IMV) IMV = 0.
   IF CRRT_tid > 0 CRRT = 1.
   IF sysmis(CRRT) CRRT = 0.
      EXECUTE.

NUMERIC ICU_LoS(f6).
```

```
    VARIABLE LABELS ICU_LoS 'Total sammanhängande vårdtid på IVA, days.'.
  COMPUTE ICU_LoS = datediff(UtskrTid,InskrTid,'hours').
  COMPUTE ICU_LoS = ICU_LoS/24.
  EXECUTE.
NUMERIC Dead_ICU_n(f1).
  IF Avliden_IVA = 'Ja' DeadICU_n = 1.
  IF sysmis(DeadICU_n) DeadICU_n = 0.
IF DeadICU_n = 1 DeathDate_ICU = UtskrivDatum.
  FORMATS DeathDate_ICU(date11).
NUMERIC Dead_n(f2).
  IF Avliden = 'Ja' Dead_n = 1.
  IF sysmis(Dead_n) Dead_n = 0.
IF Dead_n = 1 DeathDate = AvregDatum.
  FORMATS DeathDate(date11).

  ADD FILES FILE*
  /KEEP ID InskrTid UtskrTid InskrivDatum UtskrivDatum DeadICU_n DeathDate_ICU Dead_n DeathDate
SjukhusTyp Alder Kon Opererad SAPS3Score SAPS3_u_comorb
  SAPS3ScoreBoxIII Operationstyp APACHE_Score APACHE_AkutFysPoang Status IMV_tid CRRT_tid Sepsis_grad
Sepsis IMV CRRT ICU_LoS.
    EXECUTE.

SAVE OUTFILE 'Sepsis_temp.sav'.
*lägg till CCI och diagnoser.

*Hämta ut_par_sv_8996_2017.sav.

Get file 'Y:\Original_SPSS\ut_par_sv_8996_2017.sav'.
DATASET NAME Soc WINDOW=FRONT.
DATASET ACTIVATE Soc.
```

```
RENAME VARIABLES (lopnr LKF Kon = ID L Kon).
    FORMATS ID(f10).
        EXECUTE.
DELETE VARIABLES KON FODDAT ALDER to UTDATUMA UTDATUMA PVARD HDIA DIA_ANT SENUTV drg
SJUKHUSNAMN.
    EXECUTE.
STRING LKF(a10).
COMPUTE LKF = concat(rtrim(LK),' ',rtrim(L)).
    EXECUTE.
    DELETE VARIABLES LK L.
        EXECUTE.
SORT CASES ID(a) INDATUM(a) UTDATUM(a).
    NUMERIC CounterVTF(f3).
    IF $casenum = 1 or ID ne lag(ID) CounterVTF = 1.
    IF ID = lag(ID) CounterVTF = lag(CounterVTF) + 1.
        EXECUTE.

DATASET ACTIVATE Soc.
MATCH FILES /FILE=*
  /TABLE='Sepsis'
  /BY ID.
EXECUTE.

DATASET CLOSE Sepsis.
    DATASET ACTIVATE Soc.
    DATASET NAME Sepsis.
    DATASET ACTIVATE Sepsis.

SELECT IF InskrTid >0.
```

```
   EXECUTE.

DELETE VARIABLES CounterVTF.
   EXECUTE.
SORT CASES ID(a) INDATUM(a) UTDATUM(a).
   NUMERIC CounterVTF(f3).
   IF $casenum = 1 or ID ne lag(ID) CounterVTF = 1.
   IF ID = lag(ID) CounterVTF = lag(CounterVTF) + 1.
      EXECUTE.

SAVE outfile 'Sepsis_soc.sav'.
  * GET FILE 'Sepsis_soc.sav'.
  * DATASET NAME Sepsis WINDOW=FRONT.
  * DATASET ACTIVATE Sepsis.
*Skapa comorbiditetsvariabeln.
DATASET NAME Sepsis_CCI.

SELECT IF INDATUM < InskrivDatum.
   EXECUTE.

ADD FILES FILE*/KEEP ID CounterVTF Diagnos.
   EXECUTE.

SORT CASES BY ID CounterVTF.
CASESTOVARS
  /ID=ID
  /INDEX=CounterVTF
  /GROUPBY=VARIABLE.
```

```
*Slå ihop diagnosvariablerna.
STRING v(A3245).
VECTOR v=DIAGNOS.1 TO DIAGNOS.257.
LOOP cnt=1 TO 257.
- COMPUTE v=CONCAT(RTRIM(v)," ",v(cnt)," ") .
END LOOP.
- COMPUTE v=CONCAT(RTRIM(v),v(257)) .
   EXECUTE.

VARIABLE WIDTH  v (15).

RENAME VARIABLES v = Comorbidity_IVA.
VARIABLE LABELS Comorbidity_IVA 'Comorbiditet till och med vtf före in IVA'.

*Räkna tecken i Comorbidity_IVA så att Kolla att diagnoserna får plats (3237).
compute count_Comorbidity_IVA=char.length(Comorbidity_IVA).
   EXECUTE.
DESCRIPTIVES VARIABLES=count_Comorbidity_IVA
  /STATISTICS=MEAN MIN MAX.

*Ta bort överblivna variabler.
   DELETE VARIABLES Diagnos.1 to Diagnos.257 count_Comorbidity_IVA cnt.
    EXECUTE.

RENAME VARIABLES (Comorbidity_IVA = ICD).

*Create CCI according to Quan.
NUMERIC CCI_1(f2).
NUMERIC CCI_2(f2).
```

```
NUMERIC CCI_3(f2).
NUMERIC CCI_4(f2).
NUMERIC CCI_5(f2).
NUMERIC CCI_6(f2).
NUMERIC CCI_7(f2).
NUMERIC CCI_8(f2).
NUMERIC CCI_9(f2).
NUMERIC CCI_10(f2).
NUMERIC CCI_11(f2).
NUMERIC CCI_12(f2).
NUMERIC CCI_13(f2).
NUMERIC CCI_14(f2).
NUMERIC CCI_15(f2).
NUMERIC CCI_16(f2).
NUMERIC CCI_17(f2).
Numeric CCI(f8).
  VARIABLE LABELS CCI 'CCI according to Quan'.

*The code will give CCI.
*CCI 1.
COMPUTE CCI_1 =
  (CHAR.INDEX(UPCASE(ICD),'I21') > 0) OR
  (CHAR.INDEX(UPCASE(ICD),'I22') > 0) OR
  (CHAR.INDEX(UPCASE(ICD),'I252') > 0).

    EXECUTE.
  VARIABLE LABELS CCI_1 'Myocardial Infarction'.
  VALUE LABELS CCI_1 0 'no myocardial infarction' 1 'myocardial infarction'.
```

```
*CCI 2.

COMPUTE CCI_2 =
    (CHAR.INDEX(UPCASE(ICD),'I43') > 0) OR
    (CHAR.INDEX(UPCASE(ICD),'I50') > 0) OR
    (CHAR.INDEX(UPCASE(ICD),'I099') > 0) OR
    (CHAR.INDEX(UPCASE(ICD),'I110') > 0) OR
    (CHAR.INDEX(UPCASE(ICD),'I130') > 0) OR
    (CHAR.INDEX(UPCASE(ICD),'I132') > 0) OR
    (CHAR.INDEX(UPCASE(ICD),'I255') > 0) OR
    (CHAR.INDEX(UPCASE(ICD),'I420') > 0) OR
    (CHAR.INDEX(UPCASE(ICD),'I425') > 0) OR
    (CHAR.INDEX(UPCASE(ICD),'I426') > 0) OR
    (CHAR.INDEX(UPCASE(ICD),'I427') > 0) OR
    (CHAR.INDEX(UPCASE(ICD),'I428') > 0) OR
    (CHAR.INDEX(UPCASE(ICD),'I429') > 0) OR
    (CHAR.INDEX(UPCASE(ICD),'P290') > 0).

      EXECUTE.
   VARIABLE LABELS CCI_2 'Congestive Heart Failure'.
   VALUE LABELS CCI_2 0 'no Congestive Heart Failure' 1 'Congestive Heart Failure'.

*CCI 3.

COMPUTE CCI_3 =
   (CHAR.INDEX(UPCASE(ICD),'I70') > 0) OR
   (CHAR.INDEX(UPCASE(ICD),'I71') > 0) OR
   (CHAR.INDEX(UPCASE(ICD),'I731') > 0) OR
   (CHAR.INDEX(UPCASE(ICD),'I738') > 0) OR
   (CHAR.INDEX(UPCASE(ICD),'I739') > 0) OR
   (CHAR.INDEX(UPCASE(ICD),'I771') > 0) OR
```

```
   (CHAR.INDEX(UPCASE(ICD),'I790') > 0) OR
   (CHAR.INDEX(UPCASE(ICD),'I792') > 0) OR
   (CHAR.INDEX(UPCASE(ICD),'K551') > 0) OR
   (CHAR.INDEX(UPCASE(ICD),'K558') > 0) OR
   (CHAR.INDEX(UPCASE(ICD),'K559') > 0) OR
   (CHAR.INDEX(UPCASE(ICD),'Z958') > 0) OR
   (CHAR.INDEX(UPCASE(ICD),'Z959') > 0).

     EXECUTE.
   VARIABLE LABELS CCI_3 'Peripheral Vascular Disease'.
   VALUE LABELS CCI_3 0 'no Peripheral Vascular Disease' 1 'Peripheral Vascular Disease'.

*CCI 4.
COMPUTE CCI_4 =
   (CHAR.INDEX(UPCASE(ICD),'G45') > 0) OR
   (CHAR.INDEX(UPCASE(ICD),'G46') > 0) OR
   (CHAR.INDEX(UPCASE(ICD),'H340') > 0) OR
   (CHAR.INDEX(UPCASE(ICD),'I6') > 0).

     EXECUTE.
   VARIABLE LABELS CCI_4 'Cerebrovascular Disease'.
   VALUE LABELS CCI_4 0 'no Cerebrovascular Disease' 1 'Cerebrovascular Disease'.



*CCI 5.
COMPUTE CCI_5 =
   (CHAR.INDEX(UPCASE(ICD),'F00') > 0) OR
   (CHAR.INDEX(UPCASE(ICD),'F01') > 0) OR
   (CHAR.INDEX(UPCASE(ICD),'F02') > 0) OR
```

```
    (CHAR.INDEX(UPCASE(ICD),'F03') > 0) OR
    (CHAR.INDEX(UPCASE(ICD),'F051') > 0) OR
    (CHAR.INDEX(UPCASE(ICD),'G30') > 0) OR
    (CHAR.INDEX(UPCASE(ICD),'G311') > 0).

        EXECUTE.
VARIABLE LABELS CCI_5 'Dementia'.
VALUE LABELS CCI_5 0 'no Dementia' 1 'Dementia'.

*CCI 6.
COMPUTE CCI_6 =
    (CHAR.INDEX(UPCASE(ICD),'I278') > 0) OR
    (CHAR.INDEX(UPCASE(ICD),'I279') > 0) OR
    (CHAR.INDEX(UPCASE(ICD),'IJ40') > 0) OR
    (CHAR.INDEX(UPCASE(ICD),'J41') > 0) OR
    (CHAR.INDEX(UPCASE(ICD),'J42') > 0) OR
    (CHAR.INDEX(UPCASE(ICD),'J43') > 0) OR
    (CHAR.INDEX(UPCASE(ICD),'J44') > 0) OR
    (CHAR.INDEX(UPCASE(ICD),'J45') > 0) OR
    (CHAR.INDEX(UPCASE(ICD),'J46') > 0) OR
    (CHAR.INDEX(UPCASE(ICD),'J47') > 0) OR
    (CHAR.INDEX(UPCASE(ICD),'J60') > 0) OR
    (CHAR.INDEX(UPCASE(ICD),'J61') > 0) OR
    (CHAR.INDEX(UPCASE(ICD),'J62') > 0) OR
    (CHAR.INDEX(UPCASE(ICD),'J63') > 0) OR
    (CHAR.INDEX(UPCASE(ICD),'J64') > 0) OR
    (CHAR.INDEX(UPCASE(ICD),'J65') > 0) OR
    (CHAR.INDEX(UPCASE(ICD),'J66') > 0) OR
    (CHAR.INDEX(UPCASE(ICD),'J67') > 0) OR
```

```
    (CHAR.INDEX(UPCASE(ICD),'J684') > 0) OR
    (CHAR.INDEX(UPCASE(ICD),'J701') > 0) OR
    (CHAR.INDEX(UPCASE(ICD),'J703') > 0).

        EXECUTE.
VARIABLE LABELS CCI_6 'Chronic Pulmonary Disease'.
VALUE LABELS CCI_6 0 'no Chronic Pulmonary Disease' 1 'Chronic Pulmonary Disease'.

*CCI 7.
COMPUTE CCI_7 =
    (CHAR.INDEX(UPCASE(ICD),'M05') > 0) OR
    (CHAR.INDEX(UPCASE(ICD),'M06') > 0) OR
    (CHAR.INDEX(UPCASE(ICD),'M315') > 0) OR
    (CHAR.INDEX(UPCASE(ICD),'M32') > 0) OR
    (CHAR.INDEX(UPCASE(ICD),'M33') > 0) OR
    (CHAR.INDEX(UPCASE(ICD),'M34') > 0) OR
    (CHAR.INDEX(UPCASE(ICD),'M351') > 0) OR
    (CHAR.INDEX(UPCASE(ICD),'M353') > 0) OR
    (CHAR.INDEX(UPCASE(ICD),'M360') > 0) OR
    (CHAR.INDEX(UPCASE(ICD),'J47') > 0).

        EXECUTE.
VARIABLE LABELS CCI_7 'Connective Tissue Disease-Rheumatic Disease'.
VALUE LABELS CCI_7 0 'no Connective Tissue Disease-Rheumatic Disease' 1 'Connective Tissue Disease-
Rheumatic Disease'.

*CCI 8.
COMPUTE CCI_8 =
    (CHAR.INDEX(UPCASE(ICD),'K25') > 0) OR
    (CHAR.INDEX(UPCASE(ICD),'K26') > 0) OR
```

```
    (CHAR.INDEX(UPCASE(ICD),'K27') > 0) OR
    (CHAR.INDEX(UPCASE(ICD),'K28') > 0).

      EXECUTE.
VARIABLE LABELS CCI_8 'Peptic Ulcer Disease'.
VALUE LABELS CCI_8 0 'no Peptic Ulcer Disease' 1 'Peptic Ulcer Disease'.

*CCI 9.
COMPUTE CCI_9 = (CHAR.INDEX(UPCASE(ICD),'B18') > 0) OR
    (CHAR.INDEX(UPCASE(ICD),'K700') > 0) OR
    (CHAR.INDEX(UPCASE(ICD),'K701') > 0) OR
    (CHAR.INDEX(UPCASE(ICD),'K702') > 0) OR
    (CHAR.INDEX(UPCASE(ICD),'K703') > 0) OR
    (CHAR.INDEX(UPCASE(ICD),'K709') > 0) OR
    (CHAR.INDEX(UPCASE(ICD),'K713') > 0) OR
    (CHAR.INDEX(UPCASE(ICD),'K714') > 0) OR
    (CHAR.INDEX(UPCASE(ICD),'K715') > 0) OR
    (CHAR.INDEX(UPCASE(ICD),'K717') > 0) OR
    (CHAR.INDEX(UPCASE(ICD),'K73') > 0) OR
    (CHAR.INDEX(UPCASE(ICD),'K74') > 0) OR
    (CHAR.INDEX(UPCASE(ICD),'K760') > 0) OR
    (CHAR.INDEX(UPCASE(ICD),'K762') > 0) OR
    (CHAR.INDEX(UPCASE(ICD),'K763') > 0) OR
    (CHAR.INDEX(UPCASE(ICD),'K764') > 0) OR
    (CHAR.INDEX(UPCASE(ICD),'K768') > 0) OR
    (CHAR.INDEX(UPCASE(ICD),'K768') > 0) OR
    (CHAR.INDEX(UPCASE(ICD),'Z944') > 0).

      EXECUTE.
```

```
VARIABLE LABELS CCI_9 'Mild Liver Disease'.

VALUE LABELS CCI_9 0 'no Mild Liver Disease' 1 'Mild Liver Disease'.


*CCI 10.

COMPUTE CCI_10 =

   (CHAR.INDEX(UPCASE(ICD),'E100') > 0) OR

   (CHAR.INDEX(UPCASE(ICD),'E101') > 0) OR

   (CHAR.INDEX(UPCASE(ICD),'E106') > 0) OR

   (CHAR.INDEX(UPCASE(ICD),'E108') > 0) OR

   (CHAR.INDEX(UPCASE(ICD),'E109') > 0) OR

   (CHAR.INDEX(UPCASE(ICD),'E110') > 0) OR

   (CHAR.INDEX(UPCASE(ICD),'E111') > 0) OR

   (CHAR.INDEX(UPCASE(ICD),'E116') > 0) OR

   (CHAR.INDEX(UPCASE(ICD),'E118') > 0) OR

   (CHAR.INDEX(UPCASE(ICD),'E119') > 0) OR

   (CHAR.INDEX(UPCASE(ICD),'E120') > 0) OR

   (CHAR.INDEX(UPCASE(ICD),'E121') > 0) OR

   (CHAR.INDEX(UPCASE(ICD),'E126') > 0) OR

   (CHAR.INDEX(UPCASE(ICD),'E128') > 0) OR

   (CHAR.INDEX(UPCASE(ICD),'E129') > 0) OR

   (CHAR.INDEX(UPCASE(ICD),'E130') > 0) OR

   (CHAR.INDEX(UPCASE(ICD),'E131') > 0) OR

   (CHAR.INDEX(UPCASE(ICD),'E136') > 0) OR

   (CHAR.INDEX(UPCASE(ICD),'E138') > 0) OR

   (CHAR.INDEX(UPCASE(ICD),'E139') > 0) OR

   (CHAR.INDEX(UPCASE(ICD),'E140') > 0) OR

   (CHAR.INDEX(UPCASE(ICD),'E141') > 0) OR

   (CHAR.INDEX(UPCASE(ICD),'E146') > 0) OR

   (CHAR.INDEX(UPCASE(ICD),'E148') > 0) OR
```

```
   (CHAR.INDEX(UPCASE(ICD),'E149') > 0).

      EXECUTE.
VARIABLE LABELS CCI_10 'Diabetes without complications'.
VALUE LABELS CCI_10 0 'no Diabetes without complications' 1 'Diabetes without complications'.

*CCI 11.
COMPUTE CCI_11 =
   (CHAR.INDEX(UPCASE(ICD),'E102') > 0) OR
   (CHAR.INDEX(UPCASE(ICD),'E103') > 0) OR
   (CHAR.INDEX(UPCASE(ICD),'E104') > 0) OR
   (CHAR.INDEX(UPCASE(ICD),'E105') > 0) OR
   (CHAR.INDEX(UPCASE(ICD),'E107') > 0) OR
   (CHAR.INDEX(UPCASE(ICD),'E112') > 0) OR
   (CHAR.INDEX(UPCASE(ICD),'E113') > 0) OR
   (CHAR.INDEX(UPCASE(ICD),'E114') > 0) OR
   (CHAR.INDEX(UPCASE(ICD),'E115') > 0) OR
   (CHAR.INDEX(UPCASE(ICD),'E117') > 0) OR
   (CHAR.INDEX(UPCASE(ICD),'E122') > 0) OR
   (CHAR.INDEX(UPCASE(ICD),'E123') > 0) OR
   (CHAR.INDEX(UPCASE(ICD),'E124') > 0) OR
   (CHAR.INDEX(UPCASE(ICD),'E125') > 0) OR
   (CHAR.INDEX(UPCASE(ICD),'E127') > 0) OR
   (CHAR.INDEX(UPCASE(ICD),'E132') > 0) OR
   (CHAR.INDEX(UPCASE(ICD),'E133') > 0) OR
   (CHAR.INDEX(UPCASE(ICD),'E134') > 0) OR
   (CHAR.INDEX(UPCASE(ICD),'E135') > 0) OR
   (CHAR.INDEX(UPCASE(ICD),'E137') > 0) OR
   (CHAR.INDEX(UPCASE(ICD),'E142') > 0) OR
```

```
   (CHAR.INDEX(UPCASE(ICD),'E143') > 0) OR
   (CHAR.INDEX(UPCASE(ICD),'E144') > 0) OR
   (CHAR.INDEX(UPCASE(ICD),'E145') > 0) OR
   (CHAR.INDEX(UPCASE(ICD),'E147') > 0).

     EXECUTE.
VARIABLE LABELS CCI_11 'Diabetes with chronic complication'.
VALUE LABELS CCI_11 0 'no Diabetes with chronic complication' 1 'Diabetes with chronic complication'.

*CCI 12.
COMPUTE CCI_12 =
   (CHAR.INDEX(UPCASE(ICD),'G041') > 0) OR
   (CHAR.INDEX(UPCASE(ICD),'G114') > 0) OR
   (CHAR.INDEX(UPCASE(ICD),'G801') > 0) OR
   (CHAR.INDEX(UPCASE(ICD),'G802') > 0) OR
   (CHAR.INDEX(UPCASE(ICD),'G81') > 0) OR
   (CHAR.INDEX(UPCASE(ICD),'G82') > 0) OR
   (CHAR.INDEX(UPCASE(ICD),'G830') > 0) OR
   (CHAR.INDEX(UPCASE(ICD),'G831') > 0) OR
   (CHAR.INDEX(UPCASE(ICD),'G832') > 0) OR
   (CHAR.INDEX(UPCASE(ICD),'G833') > 0) OR
   (CHAR.INDEX(UPCASE(ICD),'G834') > 0) OR
   (CHAR.INDEX(UPCASE(ICD),'G839') > 0).

     EXECUTE.
VARIABLE LABELS CCI_12 'Hemiplegia or paraplegia'.
VALUE LABELS CCI_12 0 'no Hemiplegia or paraplegia' 1 'Hemiplegia or paraplegia'.

*CCI 13.
```

```
COMPUTE CCI_13 =
    (CHAR.INDEX(UPCASE(ICD),'I120') > 0) OR
    (CHAR.INDEX(UPCASE(ICD),'I131') > 0) OR
    (CHAR.INDEX(UPCASE(ICD),'N032') > 0) OR
    (CHAR.INDEX(UPCASE(ICD),'N033') > 0) OR
    (CHAR.INDEX(UPCASE(ICD),'N034') > 0) OR
    (CHAR.INDEX(UPCASE(ICD),'N035') > 0) OR
    (CHAR.INDEX(UPCASE(ICD),'N036') > 0) OR
    (CHAR.INDEX(UPCASE(ICD),'N037') > 0) OR
    (CHAR.INDEX(UPCASE(ICD),'N052') > 0) OR
    (CHAR.INDEX(UPCASE(ICD),'N053') > 0) OR
    (CHAR.INDEX(UPCASE(ICD),'N054') > 0) OR
    (CHAR.INDEX(UPCASE(ICD),'N055') > 0) OR
    (CHAR.INDEX(UPCASE(ICD),'N056') > 0) OR
    (CHAR.INDEX(UPCASE(ICD),'N057') > 0) OR
    (CHAR.INDEX(UPCASE(ICD),'N18') > 0) OR
    (CHAR.INDEX(UPCASE(ICD),'N19') > 0) OR
    (CHAR.INDEX(UPCASE(ICD),'N250') > 0) OR
    (CHAR.INDEX(UPCASE(ICD),'Z490') > 0) OR
    (CHAR.INDEX(UPCASE(ICD),'Z491') > 0) OR
    (CHAR.INDEX(UPCASE(ICD),'Z492') > 0) OR
    (CHAR.INDEX(UPCASE(ICD),'Z940') > 0) OR
    (CHAR.INDEX(UPCASE(ICD),'Z992') > 0).

        EXECUTE.
VARIABLE LABELS CCI_13 'Renal disease'.
VALUE LABELS CCI_13 0 'no Renal disease' 1 'Renal disease'.

*CCI 14.
```

```
COMPUTE CCI_14 =
  (CHAR.INDEX(UPCASE(ICD),'C0') > 0) OR
  (CHAR.INDEX(UPCASE(ICD),'C30') > 0) OR
  (CHAR.INDEX(UPCASE(ICD),'C31') > 0) OR
  (CHAR.INDEX(UPCASE(ICD),'C32') > 0) OR
  (CHAR.INDEX(UPCASE(ICD),'C33') > 0) OR
  (CHAR.INDEX(UPCASE(ICD),'C34') > 0) OR
  (CHAR.INDEX(UPCASE(ICD),'C37') > 0) OR
  (CHAR.INDEX(UPCASE(ICD),'C38') > 0) OR
  (CHAR.INDEX(UPCASE(ICD),'C39') > 0) OR
  (CHAR.INDEX(UPCASE(ICD),'C40') > 0) OR
  (CHAR.INDEX(UPCASE(ICD),'C41') > 0) OR
  (CHAR.INDEX(UPCASE(ICD),'C43') > 0) OR
  (CHAR.INDEX(UPCASE(ICD),'C45') > 0) OR
  (CHAR.INDEX(UPCASE(ICD),'C46') > 0) OR
  (CHAR.INDEX(UPCASE(ICD),'C47') > 0) OR
  (CHAR.INDEX(UPCASE(ICD),'C48') > 0) OR
  (CHAR.INDEX(UPCASE(ICD),'C49') > 0) OR
  (CHAR.INDEX(UPCASE(ICD),'C50') > 0) OR
  (CHAR.INDEX(UPCASE(ICD),'C51') > 0) OR
  (CHAR.INDEX(UPCASE(ICD),'C52') > 0) OR
  (CHAR.INDEX(UPCASE(ICD),'C53') > 0) OR
  (CHAR.INDEX(UPCASE(ICD),'C54') > 0) OR
  (CHAR.INDEX(UPCASE(ICD),'C55') > 0) OR
  (CHAR.INDEX(UPCASE(ICD),'C56') > 0) OR
  (CHAR.INDEX(UPCASE(ICD),'C57') > 0) OR
  (CHAR.INDEX(UPCASE(ICD),'C58') > 0) OR
  (CHAR.INDEX(UPCASE(ICD),'C6') > 0) OR
  (CHAR.INDEX(UPCASE(ICD),'C70') > 0) OR
```

```
  (CHAR.INDEX(UPCASE(ICD),'C71') > 0) OR
  (CHAR.INDEX(UPCASE(ICD),'C72') > 0) OR
  (CHAR.INDEX(UPCASE(ICD),'C73') > 0) OR
  (CHAR.INDEX(UPCASE(ICD),'C74') > 0) OR
  (CHAR.INDEX(UPCASE(ICD),'C75') > 0) OR
  (CHAR.INDEX(UPCASE(ICD),'C76') > 0) OR
  (CHAR.INDEX(UPCASE(ICD),'C81') > 0) OR
  (CHAR.INDEX(UPCASE(ICD),'C82') > 0) OR
  (CHAR.INDEX(UPCASE(ICD),'C83') > 0) OR
  (CHAR.INDEX(UPCASE(ICD),'C84') > 0) OR
  (CHAR.INDEX(UPCASE(ICD),'C85') > 0) OR
  (CHAR.INDEX(UPCASE(ICD),'C88') > 0) OR
  (CHAR.INDEX(UPCASE(ICD),'C90') > 0) OR
  (CHAR.INDEX(UPCASE(ICD),'C91') > 0) OR
  (CHAR.INDEX(UPCASE(ICD),'C92') > 0) OR
  (CHAR.INDEX(UPCASE(ICD),'C93') > 0) OR
  (CHAR.INDEX(UPCASE(ICD),'C94') > 0) OR
  (CHAR.INDEX(UPCASE(ICD),'C95') > 0) OR
  (CHAR.INDEX(UPCASE(ICD),'C96') > 0) OR
  (CHAR.INDEX(UPCASE(ICD),'C97') > 0).

    EXECUTE.
  VARIABLE LABELS CCI_14 'Any malignancy, including lymphoma and leukemia, except malignant neoplasm of skin'.
  VALUE LABELS CCI_14 0 'no Any malignancy, including lymphoma and leukemia, except malignant neoplasm of skin' 1 'Any malignancy, including lymphoma and leukemia, except malignant neoplasm of skin'.

*CCI 15.
COMPUTE CCI_15 =
  (CHAR.INDEX(UPCASE(ICD),'I850') > 0) OR
```

```
   (CHAR.INDEX(UPCASE(ICD),'I859') > 0) OR
   (CHAR.INDEX(UPCASE(ICD),'I864') > 0) OR
   (CHAR.INDEX(UPCASE(ICD),'I982') > 0) OR
   (CHAR.INDEX(UPCASE(ICD),'K704') > 0) OR
   (CHAR.INDEX(UPCASE(ICD),'K711') > 0) OR
   (CHAR.INDEX(UPCASE(ICD),'K721') > 0) OR
   (CHAR.INDEX(UPCASE(ICD),'K729') > 0) OR
   (CHAR.INDEX(UPCASE(ICD),'K765') > 0) OR
   (CHAR.INDEX(UPCASE(ICD),'K766') > 0) OR
   (CHAR.INDEX(UPCASE(ICD),'K767') > 0).

      EXECUTE.
   VARIABLE LABELS CCI_15 'Moderate or severe liver disease'.
   VALUE LABELS CCI_15 0 'no Moderate or severe liver disease' 1 'Moderate or severe liver disease'.

*CCI 16.
COMPUTE CCI_16 =
   (CHAR.INDEX(UPCASE(ICD),'C77') > 0) OR
   (CHAR.INDEX(UPCASE(ICD),'C78') > 0) OR
   (CHAR.INDEX(UPCASE(ICD),'C79') > 0) OR
   (CHAR.INDEX(UPCASE(ICD),'C80')  > 0).

      EXECUTE.
   VARIABLE LABELS CCI_16 'Metastatic solid tumor'.
   VALUE LABELS CCI_16 0 'no Metastatic solid tumor' 1 'Metastatic solid tumor'.

*CCI 17.
COMPUTE CCI_17 =
   (CHAR.INDEX(UPCASE(ICD),'B20') > 0) OR
```

```
    (CHAR.INDEX(UPCASE(ICD),'B21') > 0) OR
    (CHAR.INDEX(UPCASE(ICD),'B22') > 0) OR
    (CHAR.INDEX(UPCASE(ICD),'B24') > 0).

        EXECUTE.
VARIABLE LABELS CCI_17 'AIDS/HIV'.
VALUE LABELS CCI_17 0 'no AIDS/HIV' 1 'AIDS/HIV'.

RECODE CCI_1 to CCI_17 (0=0) (Else = 1).
    EXECUTE.
*Add up CCI.
COMPUTE CCI = 0.
If CCI_1 > 0 CCI = CCI + 0.
If CCI_2 > 0 CCI = CCI + 2.
If CCI_3 > 0 CCI = CCI + 0.
If CCI_4 > 0 CCI = CCI + 0.
If CCI_5 > 0 CCI = CCI + 2.
If CCI_6 > 0 CCI = CCI + 1.
If CCI_7 > 0 CCI = CCI + 1.
If CCI_8 > 0 CCI = CCI + 0.
If CCI_9 > 0 CCI = CCI + 2.
If CCI_10 > 0 CCI = CCI + 0.
If CCI_11 > 0 CCI = CCI + 1.
If CCI_12 > 0 CCI = CCI + 2.
If CCI_13 > 0 CCI = CCI + 1.
If CCI_14 > 0 CCI = CCI + 2.
If CCI_15 > 0 CCI = CCI + 4.
If CCI_16 > 0 CCI = CCI + 6.
If CCI_17 > 0 CCI = CCI + 4.
```

```
   EXECUTE.

*Subtract mild liver and tumor disease from those with severe disease.
IF CCI_9 > 0 and CCI_15 > 0 CCI = CCI - 2.
IF CCI_14 > 0 and CCI_16 > 0 CCI = CCI - 2.
   EXECUTE.

   FREQUENCIES CCI_1 to CCI.

SAVE OUTFILE 'Sepsis_CCI.sav'.
ADD FILES FILE*/KEEP ID CCI.
   EXECUTE.

GET FILE 'Sepsis_soc.sav'.
DATASET NAME Sepsis WINDOW=FRONT.
DATASET ACTIVATE Sepsis.


*Hitta komorbiditeterna.
NUMERIC Tid_Diagn_Inkl(f6).
     VARIABLE LABELS Tid_Diagn_Inkl 'Tid från diagnos till inklusion'.
COMPUTE Tid_Diagn_Inkl = datediff(InskrivDatum,UTDATUM,'days').
   EXECUTE.

NUMERIC Tid_Atg_Inkl(f6).
     VARIABLE LABELS Tid_Atg_Inkl 'Tid från åtgärd till inklusion'.
COMPUTE Tid_Atg_Inkl = datediff(InskrivDatum,UTDATUM,'days').
   EXECUTE.
```

*Ta bort åtgärder och diagnoser som inte varit före IVA med Sepsis.

```
SELECT IF Tid_Diagn_Inkl > 0.
  EXECUTE.
```

*Hitta Cytoadministreringar.

```
NUMERIC CytoAdm(f2).
NUMERIC CytoAdm2(f2).
  VARIABLE LABELS CytoAdm 'Cytoadministreringar -åtgärd- sista sex månaderna'.
  VARIABLE LABELS CytoAdm2 'Cytoadministreringar -åtgärd- sista 12 månaderna'.
  VALUE LABELS CytoAdm 0 'No' 1 'Yes'.
  COMPUTE AA = (CHAR.INDEX(UPCASE(Op),'DT107') > 0).
  COMPUTE BB = (CHAR.INDEX(UPCASE(Op),'DT108') > 0).
  COMPUTE CC = (CHAR.INDEX(UPCASE(Op),'DT112') > 0).
  COMPUTE DD = (CHAR.INDEX(UPCASE(Op),'DT116') > 0).
  COMPUTE EE = (CHAR.INDEX(UPCASE(Op),'DT135') > 0).
  IF AA > 0 or  BB > 0 or CC > 0 or DD > 0  or EE > 0 CytoAdm = 1.
    If CytoAdm = 1 CytoAdm2 = 1.
    If Tid_Atg_Inkl > 365 CytoAdm2 = 0.
    IF sysmis(CytoAdm2) CytoAdm2= 0.
  If Tid_Atg_Inkl > 182 CytoAdm = 0.
  IF sysmis(CytoAdm) CytoAdm= 0.
    EXECUTE.
  DELETE VARIABLES AA BB CC DD EE.
    EXECUTE.
```

*Hitta Strålbehandlingar.

```
NUMERIC Radiak(f2).
NUMERIC Radiak2(f2).
   VARIABLE LABELS Radiak 'Strålbehandlingar -åtgärd- sista sex månaderna'.
   VARIABLE LABELS Radiak2 'Strålbehandlingar -åtgärd- sista 12 månaderna'.
   VALUE LABELS Radiak 0 'No' 1 'Yes'.
   COMPUTE AA = (CHAR.INDEX(UPCASE(Op),'DV069') > 0).
   COMPUTE BB = (CHAR.INDEX(UPCASE(Op),'DV070') > 0).
   COMPUTE CC = (CHAR.INDEX(UPCASE(Op),'DV071') > 0).
   COMPUTE DD = (CHAR.INDEX(UPCASE(Op),'DV072') > 0).
   IF AA > 0 or  BB > 0 or CC > 0 or DD > 0 Radiak = 1.
   IF Radiak = 1 Radiak2 = 1.
      If Tid_Atg_Inkl > 365 Radiak2 = 0.
      IF sysmis(Radiak2) Radiak2= 0.
   If Tid_Atg_Inkl > 182 Radiak = 0.
   IF sysmis(Radiak) Radiak= 0.
      EXECUTE.
   DELETE VARIABLES AA BB CC DD.
      EXECUTE.

*Hitta Hemodialys.
NUMERIC HD_Cron(f2).
   VARIABLE LABELS HD_Cron 'Intermittent hemodialys -åtgärd- sista sex månaderna'.
   VALUE LABELS HD_Cron 0 'No' 1 'Yes'.
   COMPUTE AA = (CHAR.INDEX(UPCASE(Op),'DR016') > 0).
   IF AA > 0 HD_Cron = 1.
   If Tid_Atg_Inkl > 182 HD_Cron = 0.
   IF sysmis(HD_Cron) HD_Cron= 0.
      EXECUTE.
   DELETE VARIABLES AA.
```

```
    EXECUTE.


*Hitta Diagnos//////////////////////////////////////////////////////////////////////////////////////////////////////////////////////////////////////. Obs 5 årr!!! fixa datasetet!!!!!.

*OBS för diabetes II, övervikt och HT söks efter lm 5 år tillbaka!!!! Samma för åtärder för  njursvikt - HD PD´och övervikt.

*Hitta Ischemisk hjärtsjukdom.

NUMERIC IHD(f2).
  VARIABLE LABELS IHD 'Ischemisk hjärtsjukdom'.
  VALUE LABELS IHD 0 'No' 1 'Yes'.
  COMPUTE AA = (CHAR.INDEX(UPCASE(Diagnos),'I20') > 0).
  COMPUTE BB = (CHAR.INDEX(UPCASE(Diagnos),'I21') > 0).
  COMPUTE CC = (CHAR.INDEX(UPCASE(Diagnos),'I22') > 0).
  COMPUTE DD = (CHAR.INDEX(UPCASE(Diagnos),'I23') > 0).
  COMPUTE EE = (CHAR.INDEX(UPCASE(Diagnos),'I24') > 0).
  COMPUTE FF = (CHAR.INDEX(UPCASE(Diagnos),'I25') > 0).
  IF AA > 0 or  BB > 0 or CC > 0 or DD > 0 or EE > 0 or FF > 0 IHD = 1.
  IF sysmis(IHD) IHD= 0.
    EXECUTE.
  DELETE VARIABLES AA BB CC DD EE FF.
    EXECUTE.


*Hitta annan hjärtsjukdom.
NUMERIC OtherHD(f2).
  VARIABLE LABELS OtherHD 'Annan hjärtsjukdom - icke IHD'.
  VALUE LABELS OtherHD 0 'No' 1 'Yes'.
  COMPUTE AA = (CHAR.INDEX(UPCASE(Diagnos),'I3') > 0).
  COMPUTE BB = (CHAR.INDEX(UPCASE(Diagnos),'I4') > 0).
  COMPUTE CC = (CHAR.INDEX(UPCASE(Diagnos),'I5') > 0).
```

```
   COMPUTE DD = (CHAR.INDEX(UPCASE(Diagnos),'I01') > 0).
   COMPUTE EE = (CHAR.INDEX(UPCASE(Diagnos),'I05') > 0).
   COMPUTE FF = (CHAR.INDEX(UPCASE(Diagnos),'I06') > 0).
   COMPUTE GG = (CHAR.INDEX(UPCASE(Diagnos),'I07') > 0).
   COMPUTE HH = (CHAR.INDEX(UPCASE(Diagnos),'I08') > 0).
   COMPUTE II = (CHAR.INDEX(UPCASE(Diagnos),'I09') > 0).
   COMPUTE JJ = (CHAR.INDEX(UPCASE(Diagnos),'I11') > 0).
   COMPUTE KK = (CHAR.INDEX(UPCASE(Diagnos),'I13') > 0).
   COMPUTE LL = (CHAR.INDEX(UPCASE(Diagnos),'I27') > 0).
   IF AA > 0 or  BB > 0 or CC > 0 or DD > 0 or EE > 0 or FF > 0 or GG > 0 or HH > 0 or II > 0 or JJ > 0 or KK > 0 or LL
> 0 OtherHD = 1.
   IF sysmis(OtherHD) OtherHD= 0.
      EXECUTE.
   DELETE VARIABLES AA BB CC DD EE FF GG HH II JJ KK LL.
      EXECUTE.

*Hitta hypertoni.
NUMERIC Hypertens(f2).
   VARIABLE LABELS Hypertens 'Hypertoni'.
   VALUE LABELS Hypertens 0 'No' 1 'Yes'.
   COMPUTE AA = (CHAR.INDEX(UPCASE(Diagnos),'I1') > 0).
   IF AA > 0 Hypertens = 1.
   IF sysmis(Hypertens) Hypertens = 0.
      EXECUTE.
   DELETE VARIABLES AA.
      EXECUTE.

*Hitta Diabetes typ I.
NUMERIC Diabetes1(f2).
   VARIABLE LABELS Diabetes1 'Diabetes typ 1'.
```

```
   VALUE LABELS Diabetes1 0 'No' 1 'Yes'.
   COMPUTE AA = (CHAR.INDEX(UPCASE(Diagnos),'E10') > 0).
   IF AA > 0 Diabetes1 = 1.
   IF sysmis(Diabetes1) Diabetes1 = 0.
      EXECUTE.
   DELETE VARIABLES AA.
      EXECUTE.

*Hitta Diabetes typ II.
NUMERIC Diabetes2(f2).
   VARIABLE LABELS Diabetes2 'Diabetes typ 2'.
   VALUE LABELS Diabetes2 0 'No' 1 'Yes'.
   COMPUTE AA = (CHAR.INDEX(UPCASE(Diagnos),'E11') > 0).
      EXECUTE.
   IF AA > 0 Diabetes2 = 1.
   IF sysmis(Diabetes2) Diabetes2 = 0.
   EXECUTE.
   DELETE VARIABLES AA.
      EXECUTE.

*Hittta stroke.
NUMERIC Stroke(f2).
   VARIABLE LABELS Stroke 'Någon Cerebrovaskulär sjukdom'.
   VALUE LABELS Stroke 0 'No' 1 'Yes'.
   COMPUTE AA = (CHAR.INDEX(UPCASE(Diagnos),'I6') > 0).
   IF AA > 0 Stroke = 1.
   IF sysmis(Stroke) Stroke = 0.
      EXECUTE.
   DELETE VARIABLES AA.
```

```
    EXECUTE.


*Hitta kronisk njursvikt.

NUMERIC Njursvikt(f2).

  VARIABLE LABELS Njursvikt 'Njursvikt'.

  VALUE LABELS Njursvikt 0 'No' 1 'Yes'.

  COMPUTE N181 = (CHAR.INDEX(UPCASE(Diagnos),'N181') > 0).

  COMPUTE N182 = (CHAR.INDEX(UPCASE(Diagnos),'N182') > 0).

  COMPUTE N183 = (CHAR.INDEX(UPCASE(Diagnos),'N183') > 0).

  COMPUTE N184 = (CHAR.INDEX(UPCASE(Diagnos),'N184') > 0).

  COMPUTE N185 = (CHAR.INDEX(UPCASE(Diagnos),'N185') > 0).

  COMPUTE N189 = (CHAR.INDEX(UPCASE(Diagnos),'N189') > 0).

  *Hitta HD, PD senaste 5 åren.

    COMPUTE DR016 = (CHAR.INDEX(UPCASE(Op),'DR016') > 0).

    COMPUTE DR024 = (CHAR.INDEX(UPCASE(Op),'DR024') > 0).

    IF N181 > 0 or  N182 > 0 or  N183 > 0 or  N184 > 0 or  N185 > 0 or  N189 > 0 or  DR016 > 0 or DR024 > 0
Njursvikt = 1.

  IF sysmis(Njursvikt) Njursvikt = 0.

  RECODE N181 to DR024 (SYSMIS = 0).

    EXECUTE.


*Hitta KOL, inklusive emfysem och kronisk bronkit.

NUMERIC KOL(f2).

  VARIABLE LABELS KOL 'KOL, emfysem, kronisk bronkit'.

  VALUE LABELS KOL 0 'No' 1 'Yes'.

  COMPUTE AA = (CHAR.INDEX(UPCASE(Diagnos),'J41') > 0).

  COMPUTE BB = (CHAR.INDEX(UPCASE(Diagnos),'J42') > 0).

  COMPUTE CC = (CHAR.INDEX(UPCASE(Diagnos),'J43') > 0).

  COMPUTE DD = (CHAR.INDEX(UPCASE(Diagnos),'J44') > 0).

  IF AA > 0 or  BB > 0 or CC > 0 or DD > 0 KOL = 1.
```

```
   IF sysmis(KOL) KOL= 0.
      EXECUTE.
   DELETE VARIABLES AA BB CC DD.
      EXECUTE.

*Hitta Astma.
NUMERIC Astma(f2).
   VARIABLE LABELS Astma 'Astma'.
   VALUE LABELS Astma 0 'No' 1 'Yes'.
   COMPUTE AA = (CHAR.INDEX(UPCASE(Diagnos),'J45') > 0).
   COMPUTE BB = (CHAR.INDEX(UPCASE(Diagnos),'J46') > 0).
   IF AA > 0 or  BB > 0 Astma = 1.
   IF sysmis(Astma) Astma = 0.
      EXECUTE.
   DELETE VARIABLES AA BB.
      EXECUTE.

*Hitta fetma inklusive överviktskirurgi.
NUMERIC Fetma(f2).
   VARIABLE LABELS Fetma 'Fetma'.
   VALUE LABELS Fetma 0 'No' 1 'Yes'.
   COMPUTE AA = (CHAR.INDEX(UPCASE(Diagnos),'E66') > 0).
   *Hitta Överviktskirurgi.
      COMPUTE BB = (CHAR.INDEX(UPCASE(Op),'JDF') > 0).
      COMPUTE CC = (CHAR.INDEX(UPCASE(Op),'JFD03') > 0).
      IF AA > 0 or  BB > 0 or CC > 0 Fetma = 1.
   IF sysmis(Fetma) Fetma = 0.
      EXECUTE.
   DELETE VARIABLES AA BB CC.
```

```
    EXECUTE.


*Hitta Immunsupprimerande sjukdom eller efter åtgärd.
NUMERIC Immunsupprimerad(f2).
  VARIABLE LABELS Immunsupprimerad 'Immunsupprimerad'.
  VALUE LABELS Immunsupprimerad 0 'No' 1 'Yes'.
  COMPUTE AA = (CHAR.INDEX(UPCASE(Diagnos),'D71') > 0).
  COMPUTE BB = (CHAR.INDEX(UPCASE(Diagnos),'D80') > 0).
  COMPUTE CC = (CHAR.INDEX(UPCASE(Diagnos),'D81') > 0).
  COMPUTE DD = (CHAR.INDEX(UPCASE(Diagnos),'D82') > 0).
  COMPUTE EE = (CHAR.INDEX(UPCASE(Diagnos),'D83') > 0).
  COMPUTE FF = (CHAR.INDEX(UPCASE(Diagnos),'D84') > 0).
  COMPUTE GG = (CHAR.INDEX(UPCASE(Diagnos),'D89') > 0).
  IF AA > 0 or  BB > 0 or CC > 0 or DD > 0 or EE > 0 or FF > 0 or GG > 0  Immunsupprimerad = 1.
  *Hitta immunsupprimerande åtgärder lm senaste 6 månaderna.
  IF CytoAdm = 1 or Radiak = 1 Immunsupprimerad = 1.
  IF sysmis(Immunsupprimerad)  Immunsupprimerad = 0.
    EXECUTE.
  DELETE VARIABLES AA BB CC DD EE FF GG.
    EXECUTE.


*Hitta aktiv solid tumör senaste året dvs diagnossatt Senaste 12 mån..
NUMERIC Cancer_dia(f2).
NUMERIC CancerDiagn(f2).
  VARIABLE LABELS Cancer_dia 'Cancer'.
  VALUE LABELS Cancer_dia 0 'No' 1 'Yes'.
  COMPUTE AA = (CHAR.INDEX(UPCASE(Diagnos),'C0') > 0).
  COMPUTE BB = (CHAR.INDEX(UPCASE(Diagnos),'C1') > 0).
  COMPUTE CC = (CHAR.INDEX(UPCASE(Diagnos),'C2') > 0).
```

```
   COMPUTE DD = (CHAR.INDEX(UPCASE(Diagnos),'C3') > 0).
   COMPUTE EE = (CHAR.INDEX(UPCASE(Diagnos),'C4') > 0).
   COMPUTE FF = (CHAR.INDEX(UPCASE(Diagnos),'C5') > 0).
   COMPUTE GG = (CHAR.INDEX(UPCASE(Diagnos),'C6') > 0).
   COMPUTE HH = (CHAR.INDEX(UPCASE(Diagnos),'C7') > 0).
   COMPUTE II = (CHAR.INDEX(UPCASE(Diagnos),'C80') > 0).
   COMPUTE JJ = (CHAR.INDEX(UPCASE(Diagnos),'C97') > 0).
    IF AA > 0 or  BB > 0 or CC > 0 or DD > 0 or EE > 0 or FF > 0 or GG > 0 or HH > 0 or II > 0 or JJ > 0 CancerDiagn =
1.
   IF Tid_Diagn_Inkl > 365 CancerDiagn = 0.
   If CancerDiagn = 1 Cancer_dia = 1.
   IF sysmis(Cancer_dia) Cancer_dia = 0.
      EXECUTE.
   DELETE VARIABLES AA to JJ CancerDiagn.
      EXECUTE.

*Hitta aktiv blodcancer senaste året dvs diagnossatt Senaste 12 mån..
NUMERIC BlodCancer(f2).
NUMERIC BlodCancerDiagn(f2).
   VARIABLE LABELS BlodCancer 'Hematologisk malignitet'.
   VALUE LABELS BlodCancer 0 'No' 1 'Yes'.
   COMPUTE AA = (CHAR.INDEX(UPCASE(Diagnos),'C81') > 0).
   COMPUTE BB = (CHAR.INDEX(UPCASE(Diagnos),'C82') > 0).
   COMPUTE CC = (CHAR.INDEX(UPCASE(Diagnos),'C83') > 0).
   COMPUTE DD = (CHAR.INDEX(UPCASE(Diagnos),'C84') > 0).
   COMPUTE EE = (CHAR.INDEX(UPCASE(Diagnos),'C85') > 0).
   COMPUTE FF = (CHAR.INDEX(UPCASE(Diagnos),'C86') > 0).
   COMPUTE GG = (CHAR.INDEX(UPCASE(Diagnos),'C88') > 0).
   COMPUTE HH = (CHAR.INDEX(UPCASE(Diagnos),'C90') > 0).
   COMPUTE II = (CHAR.INDEX(UPCASE(Diagnos),'C91') > 0).
```

```
COMPUTE JJ = (CHAR.INDEX(UPCASE(Diagnos),'C92') > 0).
COMPUTE KK = (CHAR.INDEX(UPCASE(Diagnos),'C93') > 0).
COMPUTE LL = (CHAR.INDEX(UPCASE(Diagnos),'C94') > 0).
COMPUTE MM = (CHAR.INDEX(UPCASE(Diagnos),'C95') > 0).
COMPUTE NN = (CHAR.INDEX(UPCASE(Diagnos),'C96') > 0).
 IF AA > 0 or  BB > 0 or CC > 0 or DD > 0 or EE > 0 or FF > 0 or GG > 0 or HH > 0 or II > 0 or JJ > 0 or KK > 0 or LL
> 0 or MM > 0 or NN > 0 BlodCancerDiagn = 1.
IF Tid_Diagn_Inkl > 365 BlodCancerDiagn = 0.
If BlodCancerDiagn = 1 BlodCancer = 1.
IF sysmis(BlodCancer) BlodCancer= 0.
   EXECUTE.
DELETE VARIABLES AA to NN BlodCancerDiagn.
   EXECUTE.

*Hitta inflammatorisk sjukdom.
NUMERIC Inflam(f2).
VARIABLE LABELS Inflam 'Systeminflammatorisk sjukdom'.
VALUE LABELS Inflam 0 'No' 1 'Yes'.
COMPUTE AA = (CHAR.INDEX(UPCASE(Diagnos),'M3') > 0).
COMPUTE BB = (CHAR.INDEX(UPCASE(Diagnos),'M05') > 0).
COMPUTE CC = (CHAR.INDEX(UPCASE(Diagnos),'M06') > 0).
COMPUTE DD = (CHAR.INDEX(UPCASE(Diagnos),'M07') > 0).
COMPUTE EE = (CHAR.INDEX(UPCASE(Diagnos),'M08') > 0).
COMPUTE FF = (CHAR.INDEX(UPCASE(Diagnos),'M09') > 0).
COMPUTE GG = (CHAR.INDEX(UPCASE(Diagnos),'M10') > 0).
COMPUTE HH = (CHAR.INDEX(UPCASE(Diagnos),'M11') > 0).
COMPUTE II = (CHAR.INDEX(UPCASE(Diagnos),'M12') > 0).
COMPUTE JJ = (CHAR.INDEX(UPCASE(Diagnos),'M13') > 0).
COMPUTE KK = (CHAR.INDEX(UPCASE(Diagnos),'M14') > 0).
```

```
   IF AA > 0 or  BB > 0 or CC > 0 or DD > 0 or EE > 0 or FF > 0 or GG > 0 or HH > 0 or II > 0 or JJ > 0 or KK > 0 Inflam
= 1.
   IF sysmis(Inflam) Inflam= 0.
      EXECUTE.
   DELETE VARIABLES AA BB CC DD EE FF GG HH II JJ KK.
      EXECUTE.
*Hitta transplanterade.
NUMERIC Transplanterad(f2).
   VARIABLE LABELS Transplanterad 'Transplanterad'.
   VALUE LABELS Transplanterad 0 'No' 1 'Yes'.
   COMPUTE AA = (CHAR.INDEX(UPCASE(Diagnos),'Z940') > 0).
   COMPUTE BB = (CHAR.INDEX(UPCASE(Diagnos),'Z941') > 0).
   COMPUTE CC = (CHAR.INDEX(UPCASE(Diagnos),'Z942') > 0).
   COMPUTE DD = (CHAR.INDEX(UPCASE(Diagnos),'Z943') > 0).
   COMPUTE EE = (CHAR.INDEX(UPCASE(Diagnos),'Z944') > 0).

   IF AA > 0 or  BB > 0 or CC > 0 or DD > 0 or EE > 0 Transplanterad = 1.
   IF sysmis(Transplanterad)  Transplanterad = 0.
      EXECUTE.
   DELETE VARIABLES AA BB CC DD EE.
      EXECUTE.

DELETE VARIABLES CounterVTF.
   EXECUTE.
SORT CASES ID(a) INDATUM(a) UTDATUM(a).
   NUMERIC CounterVTF(f3).
   IF $casenum = 1 or ID ne lag(ID) CounterVTF = 1.
   IF ID = lag(ID) CounterVTF = lag(CounterVTF) + 1.
      EXECUTE.
```

```
SORT CASES ID(a) CounterVTF(d).

EXECUTE.

*Skapa en rad per individ.

If ID = lag(ID) CytoAdm = CytoAdm + lag(CytoAdm).

IF CytoAdm > 0 CytoAdm = 1.

If ID = lag(ID) CytoAdm2 = CytoAdm2 + lag(CytoAdm2).

IF CytoAdm2 > 0 CytoAdm2 = 1.

If ID = lag(ID) Radiak = Radiak + lag(Radiak).

IF Radiak > 0 Radiak = 1.

If ID = lag(ID) Radiak2 = Radiak2 + lag(Radiak2).

IF Radiak2 > 0 Radiak2 = 1.

If ID = lag(ID) HD_Cron = HD_Cron + lag(HD_Cron).

IF HD_Cron > 0 HD_Cron = 1.

If ID = lag(ID) IHD = IHD + lag(IHD).

IF IHD > 0 IHD = 1.

If ID = lag(ID) OtherHD = OtherHD + lag(OtherHD).

IF OtherHD > 0 OtherHD = 1.

If ID = lag(ID) Hypertens = Hypertens + lag(Hypertens).

IF Hypertens > 0 Hypertens = 1.

If ID = lag(ID) Diabetes1 = Diabetes1 + lag(Diabetes1).

IF Diabetes1 > 0 Diabetes1 = 1.

If ID = lag(ID) Diabetes2 = Diabetes2 + lag(Diabetes2).

IF Diabetes2 > 0 Diabetes2 = 1.

If ID = lag(ID) Stroke = Stroke + lag(Stroke).

IF Stroke > 0 Stroke = 1.

If ID = lag(ID) Njursvikt = Njursvikt + lag(Njursvikt).

IF Njursvikt > 0 Njursvikt = 1.

If ID = lag(ID) N181 = N181 + lag(N181).

IF N181 > 0 N181 = 1.
```

```
If ID = lag(ID) N182 = N182 + lag(N182).
IF N182 > 0 N182 = 1.
If ID = lag(ID) N183 = N183 + lag(N183).
IF N183 > 0 N183 = 1.
If ID = lag(ID) N184 = N184 + lag(N184).
IF N184 > 0 N184 = 1.
If ID = lag(ID) N185 = N185 + lag(N185).
IF N185 > 0 N185 = 1.
If ID = lag(ID) N189 = N189 + lag(N189).
IF N189 > 0 N189 = 1.
If ID = lag(ID) DR016 = DR016 + lag(DR016).
IF DR016 > 0 DR016 = 1.
If ID = lag(ID) DR024 = DR024 + lag(DR024).
IF DR024 > 0 DR024 = 1.
If ID = lag(ID) KOL = KOL + lag(KOL).
IF KOL > 0 KOL = 1.
If ID = lag(ID) Astma = Astma + lag(Astma).
IF Astma > 0 Astma = 1.
If ID = lag(ID) Fetma = Fetma + lag(Fetma).
IF Fetma > 0 Fetma = 1.
If ID = lag(ID) Immunsupprimerad = Immunsupprimerad + lag(Immunsupprimerad).
IF Immunsupprimerad > 0 Immunsupprimerad = 1.
If ID = lag(ID) Cancer_dia = Cancer_dia + lag(Cancer_dia).
IF Cancer_dia > 0 Cancer_dia = 1.
If ID = lag(ID) BlodCancer = BlodCancer + lag(BlodCancer).
IF BlodCancer > 0 BlodCancer = 1.
If ID = lag(ID) Inflam = Inflam + lag(Inflam).
IF Inflam > 0 Inflam = 1.
If ID = lag(ID) Transplanterad = Transplanterad + lag(Transplanterad).
```

```
IF Transplanterad > 0 Transplanterad = 1.


*Spara ett vtf per individ.
   EXECUTE.
SELECT IF CounterVTF = 1.
   EXECUTE.


ADD FILES FILE*
   /KEEP ID CytoAdm to Transplanterad.
      EXECUTE.


SAVE OUTFILE 'Sepsis_Comorb.sav'.
   DATASET NAME Sepsis_Comorb.
GET FILE 'Sepsis_temp.sav'.
   DATASET NAME SIR WINDOW=FRONT.


DATASET ACTIVATE SIR.
MATCH FILES /FILE=*
  /FILE='Sepsis_Comorb'
  /BY ID.
EXECUTE.


DATASET ACTIVATE SIR.
MATCH FILES /FILE=*
  /TABLE='Sepsis_CCI'
  /BY ID.
EXECUTE.
   DATASET CLOSE Sepsis_CCI.
   DATASET CLOSE Sepsis_Comorb.
```

```
   DATASET ACTIVATE SIR.


RECODE CytoAdm to CCI (SYSMIS = 0) (ELSE = COPY).
   EXECUTE.
SORT CASES ID(a).



NUMERIC ARDS(f2).
NUMERIC COVID(f2).
NUMERIC COVID2(f2).
      VALUE LABELS Sepsis 0 'No sepsis' 1 'Sepsis'.
      VALUE LABELS ARDS 0 'No ARDS' 1 'ARDS'.
      VALUE LABELS Covid 0 'No Covid' 1 'Covid'.
      VARIABLE LABELS COVID2 'Covid också'.
   COMPUTE ARDS = 0.
   COMPUTE COVID = 0.
   COMPUTE COVID2 = 0.
   COMPUTE Sepsis = 1.
      EXECUTE.

SORT CASES ID(a) InskrivDatum(a) UtskrivDatum(a).
   NUMERIC CounterIVA(f3).
   IF $casenum = 1 or ID ne lag(ID) CounterIVA = 1.
   IF ID = lag(ID) CounterIVA = lag(CounterIVA) + 1.
      EXECUTE.
DESCRIPTIVES ID.
FREQUENCIES CounterIVA.

SAVE OUTFILE 'TEMP11.sav'.
```

```
   DATASET CLOSE all.
   GET FILE TEMP11.sav.
   DATASET NAME SIR WINDOW=FRONT.
SORT CASES ID.
Get file 'Y:\Original_SPSS\ut_par_sv_8996_2017.sav'.
DATASET NAME Soc WINDOW=FRONT.
DATASET ACTIVATE Soc.

RENAME VARIABLES (lopnr LKF Kon = ID L Kon).
   FORMATS ID(f10).
      EXECUTE.

STRING LKF(a10).
COMPUTE LKF = concat(rtrim(LK),' ',rtrim(L)).
   EXECUTE.
   DELETE VARIABLES LK L.
      EXECUTE.
SORT CASES ID(a) INDATUM(a) UTDATUM(a).
   NUMERIC CounterVTF(f3).
   IF $casenum = 1 or ID ne lag(ID) CounterVTF = 1.
   IF ID = lag(ID) CounterVTF = lag(CounterVTF) + 1.
      EXECUTE.
SELECT IF CounterVTF = 1.
EXECUTE.

ADD FILES FILE*
   /KEEP ID SENUTV.
```

```
        EXECUTE.
DATASET ACTIVATE SIR.
MATCH FILES /FILE=*
  /FILE='Soc'
  /BY ID.
EXECUTE.


SELECT IF Sepsis = 1.
EXECUTE.


DATASET CLOSE Soc.
    DATASET ACTIVATE Sir.
    DATASET NAME Sepsis.
    DATASET ACTIVATE Sepsis.


SELECT IF InskrivDatum >0.
    EXECUTE.
COMPUTE ID_Sepsis = ID.
    EXECUTE.
COMPUTE ID = $casenum + 200000.
    EXECUTE.


ADD FILES FILE*
/KEEP ID Alder Kon SENUTV InskrivDatum UtskrivDatum SjukhusTyp Opererad SAPS3Score SAPS3_u_comorb
SAPS3ScoreBoxIII
Operationstyp APACHE_Score APACHE_AkutFysPoang IMV_tid CRRT_tid Sepsis_grad IMV CRRT ICU_LoS
CytoAdm CytoAdm2 Radiak Radiak2 HD_Cron IHD OtherHD Hypertens Diabetes1 Diabetes2 Stroke Njursvikt
N181 N182 N183 N184 N185
N189 DR016 DR024 KOL Astma Fetma Immunsupprimerad Cancer_dia BlodCancer Inflam Transplanterad
CCI COVID COVID2 Sepsis ARDS DeadICU_n DeathDate_ICU Dead_n DeathDate ID_Sepsis.
```

```
   EXECUTE.


DATASET ACTIVATE Sepsis.
RENAME VARIABLES (InskrivDatum Alder = InskrDat Age).
   EXECUTE.
NUMERIC Sex(f1).
   VARIABLE LABELS Sex 'Sex at ICU admission'.
   VALUE LABELS Sex 1 'Man' 2 'Kvinna'.
   IF Kon = 'M' Sex = 1.
   IF Kon = 'K' Sex = 2.
FORMATS Age(f3).
   VARIABLE LABELS Age 'Age at ICU admission'.
IF Opererad = 'Ja-elektivt' Opererad = 'Ja-elek'.
IF Opererad = 'Ja-akut' Opererad = 'Ja-akut'.
ALTER TYPE Opererad(a7).
   EXECUTE.



DESCRIPTIVES ID.
FREQUENCIES Sepsis.
SELECT IF Age > 17.
   EXECUTE.
DESCRIPTIVES ID.


FREQUENCIES COVID COVID2 Sepsis ARDS.


SAVE OUTFILE 'Sepsis_komplett.sav'.
```

```
*////////////////////////////////////////////////////////////////////////////////////////////////////////////////////
/////////////.

*ARDS.Sortera bort IVA före ARDS och ej ARDS.
Get file SIR_temp2.sav.
   DATASET NAME ARDS WINDOW = FRONT.
   DATASET ACTIVATE ARDS.

*ARDS!. Sortera bort IVA fötr ARDS och ej ARDS.
SELECT IF A = 1.
EXECUTE.

DELETE VARIABLES CounterIVA.
SORT CASES ID(a) InskrivDatum(a) UtskrivDatum(a).
   NUMERIC CounterIVA(f3).
   IF $casenum = 1 or ID ne lag(ID) CounterIVA = 1.
   IF ID = lag(ID) CounterIVA = lag(CounterIVA) + 1.
      EXECUTE.
FREQUENCIES CounterIVA ARDS.

*diff är dagar mellan intensivvårdstillfällena. 0 eller 1 dags skillnad innebär samma vtf.
Compute diff = datediff(InskrTid,lag(UtskrTid),'hours').
Compute diff = Diff / 24.
   VARIABLE LABELS diff ' days from out previous to in actual ICU episode'.
   IF ID ne lag(ID) diff = $sysmis.
   IF CounterIVA = 1 X = 1.
   IF lag(X) = 1 and diff < 1 X = 1.
      EXECUTE.
SELECT IF X = 1.
   EXECUTE.
```

```
DELETE VARIABLES CounterIVA.
SORT CASES ID(a) InskrivDatum(a) UtskrivDatum(a).
  NUMERIC CounterIVA(f3).
  IF $casenum = 1 or ID ne lag(ID) CounterIVA = 1.
  IF ID = lag(ID) CounterIVA = lag(CounterIVA) + 1.
    EXECUTE.
FREQUENCIES CounterIVA ARDS.

*Skapa och spara ett vtf.
DELETE VARIABLES CounterIVA.
  EXECUTE.
SORT CASES ID(a) InskrTid(a) UtskrTid(a).
NUMERIC CounterIVA(f3).
  IF $casenum = 1 or ID ne lag(ID) CounterIVA = 1.
  IF ID = lag(ID) CounterIVA = lag(CounterIVA) + 1.
  EXECUTE.

*Åtgärder.

*Lägg ihop mot första vårdtillfället.
SORT CASES ID(a) CounterIVA(d).
  EXECUTE.
IF ID = lag(ID) CRRT_tid = CRRT_tid + lag(CRRT_tid).
IF ID = lag(ID) IMV_tid = IMV_tid + lag(IMV_tid).
IF ID = lag(ID) and lag(Avliden_IVA) = 'Ja' Avliden_IVA = 'Ja'.
IF ID = lag(ID) UtskrivDatum = lag(UtskrivDatum).
IF ID = lag(ID) UtskrTid = lag(UtskrTid).
  EXECUTE.
```

```
SELECT IF CounterIVA = 1.
   EXECUTE.
FREQUENCIES CounterIVA.


NUMERIC IMV(f2).
   VALUE LABELS IMV 1 'IMV' 0 'No IMV'.
NUMERIC CRRT(f2).
   VALUE LABELS CRRT 1 'CRRT' 0 'No CRRT'.
   IF IMV_tid > 0 IMV = 1.
   IF sysmis(IMV) IMV = 0.
   IF CRRT_tid > 0 CRRT = 1.
   IF sysmis(CRRT) CRRT = 0.
      EXECUTE.


NUMERIC ICU_LoS(f6).
      VARIABLE LABELS ICU_LoS 'Total sammanhängande vårdtid på IVA, dygn.'.
   COMPUTE ICU_LoS = datediff(UtskrTid,InskrTid,'hours').
   COMPUTE ICU_LoS = ICU_LoS/24.
   EXECUTE.
NUMERIC Dead_ICU_n(f1).
   IF Avliden_IVA = 'Ja' DeadICU_n = 1.
   IF sysmis(DeadICU_n) DeadICU_n = 0.
IF DeadICU_n = 1 DeathDate_ICU = UtskrivDatum.
   FORMATS DeathDate_ICU(date11).
NUMERIC Dead_n(f2).
   IF Avliden = 'Ja' Dead_n = 1.
   IF sysmis(Dead_n) Dead_n = 0.
IF Dead_n = 1 DeathDate = AvregDatum.
   FORMATS DeathDate(date11).
```

```
ADD FILES FILE*

/KEEP ID InskrTid UtskrTid InskrivDatum UtskrivDatum DeadICU_n DeathDate_ICU Dead_n DeathDate SjukhusTyp Alder Kon Opererad SAPS3Score SAPS3_u_comorb

SAPS3ScoreBoxIII Operationstyp APACHE_Score APACHE_AkutFysPoang Status IMV_tid CRRT_tid IMV CRRT ICU_LoS ARDS.

    EXECUTE.


SAVE OUTFILE 'ARDS_temp.sav'.

*lägg till CCI och diagnoser.

DATASET CLOSE ARDS.

GET FILE 'ARDS_temp.sav'.

  DATASET NAME ARDS WINDOW=FRONT.




Get file 'Y:\Original_SPSS\ut_par_sv_8996_2017.sav'.

DATASET NAME Soc WINDOW=FRONT.

DATASET ACTIVATE Soc.


RENAME VARIABLES (lopnr LKF Kon = ID L Kon).

  FORMATS ID(f10).

    EXECUTE.

DELETE VARIABLES KON FODDAT ALDER to UTDATUMA UTDATUMA PVARD HDIA DIA_ANT SENUTV drg SJUKHUSNAMN.

  EXECUTE.

STRING LKF(a10).

COMPUTE LKF = concat(rtrim(LK),' ',rtrim(L)).

  EXECUTE.

  DELETE VARIABLES LK L.
```

```
      EXECUTE.
SORT CASES ID(a) INDATUM(a) UTDATUM(a).
   NUMERIC CounterVTF(f3).
   IF $casenum = 1 or ID ne lag(ID) CounterVTF = 1.
   IF ID = lag(ID) CounterVTF = lag(CounterVTF) + 1.
      EXECUTE.

DATASET ACTIVATE Soc.
MATCH FILES /FILE=*
  /TABLE='ARDS'
  /BY ID.
EXECUTE.

DATASET CLOSE ARDS.
   DATASET ACTIVATE Soc.
   DATASET NAME ARDS.
   DATASET ACTIVATE ARDS.

SELECT IF InskrTid >0.
   EXECUTE.

DELETE VARIABLES CounterVTF.
   EXECUTE.
SORT CASES ID(a) INDATUM(a) UTDATUM(a).
   NUMERIC CounterVTF(f3).
   IF $casenum = 1 or ID ne lag(ID) CounterVTF = 1.
   IF ID = lag(ID) CounterVTF = lag(CounterVTF) + 1.
      EXECUTE.
```

```
SAVE outfile 'ARDS_soc.sav'.

*    GET FILE 'ARDS_soc.sav'.

*    DATASET NAME ARDS WINDOW=FRONT.

*    DATASET ACTIVATE ARDS.

*Skapa comorbiditetsvariabeln.


*Skapa comorbiditetsvariabeln.

DATASET NAME ARDS_CCI.


SELECT IF INDATUM < InskrivDatum.

   EXECUTE.


DELETE VARIABLES CounterVTF.

   EXECUTE.

SORT CASES ID(a) INDATUM(a) UTDATUM(a).

   NUMERIC CounterVTF(f3).

   IF $casenum = 1 or ID ne lag(ID) CounterVTF = 1.

   IF ID = lag(ID) CounterVTF = lag(CounterVTF) + 1.

      EXECUTE.


ADD FILES FILE*/KEEP ID CounterVTF Diagnos.

   EXECUTE.


SORT CASES BY ID CounterVTF.

CASESTOVARS

 /ID=ID

 /INDEX=CounterVTF

 /GROUPBY=VARIABLE.
```

```
*Slå ihop diagnosvariablerna.

STRING v(A2100).

VECTOR v=DIAGNOS.1 TO DIAGNOS.172.

LOOP cnt=1 TO 172.

- COMPUTE v=CONCAT(RTRIM(v)," ",v(cnt)," ") .

END LOOP.

- COMPUTE v=CONCAT(RTRIM(v),v(172)) .

  EXECUTE.

VARIABLE WIDTH  v (15).

RENAME VARIABLES v = Comorbidity_IVA.

VARIABLE LABELS Comorbidity_IVA 'Comorbiditet till och med vtf före IVA'.


*Räkna tecken i Comorbidity_IVA så att Kolla att diagnoserna får plats (2090).

compute count_Comorbidity_IVA=char.length(Comorbidity_IVA).

  EXECUTE.

DESCRIPTIVES VARIABLES=count_Comorbidity_IVA

 /STATISTICS=MEAN MIN MAX.


*Ta bort överblivna variabler.


ADD FILES FILE*/KEEP ID Comorbidity_IVA.

  EXECUTE.


RENAME VARIABLES (Comorbidity_IVA = ICD).


*Create CCI according to Quan.

NUMERIC CCI_1(f2).

NUMERIC CCI_2(f2).
```

```
NUMERIC CCI_3(f2).
NUMERIC CCI_4(f2).
NUMERIC CCI_5(f2).
NUMERIC CCI_6(f2).
NUMERIC CCI_7(f2).
NUMERIC CCI_8(f2).
NUMERIC CCI_9(f2).
NUMERIC CCI_10(f2).
NUMERIC CCI_11(f2).
NUMERIC CCI_12(f2).
NUMERIC CCI_13(f2).
NUMERIC CCI_14(f2).
NUMERIC CCI_15(f2).
NUMERIC CCI_16(f2).
NUMERIC CCI_17(f2).
Numeric CCI(f8).
  VARIABLE LABELS CCI 'CCI according to Quan'.

*The code will give CCI.
*CCI 1.
COMPUTE CCI_1 =
  (CHAR.INDEX(UPCASE(ICD),'I21') > 0) OR
  (CHAR.INDEX(UPCASE(ICD),'I22') > 0) OR
  (CHAR.INDEX(UPCASE(ICD),'I252') > 0).

    EXECUTE.
  VARIABLE LABELS CCI_1 'Myocardial Infarction'.
  VALUE LABELS CCI_1 0 'no myocardial infarction' 1 'myocardial infarction'.
```

```
*CCI 2.

COMPUTE CCI_2 =

    (CHAR.INDEX(UPCASE(ICD),'I43') > 0) OR

    (CHAR.INDEX(UPCASE(ICD),'I50') > 0) OR

    (CHAR.INDEX(UPCASE(ICD),'I099') > 0) OR

    (CHAR.INDEX(UPCASE(ICD),'I110') > 0) OR

    (CHAR.INDEX(UPCASE(ICD),'I130') > 0) OR

    (CHAR.INDEX(UPCASE(ICD),'I132') > 0) OR

    (CHAR.INDEX(UPCASE(ICD),'I255') > 0) OR

    (CHAR.INDEX(UPCASE(ICD),'I420') > 0) OR

    (CHAR.INDEX(UPCASE(ICD),'I425') > 0) OR

    (CHAR.INDEX(UPCASE(ICD),'I426') > 0) OR

    (CHAR.INDEX(UPCASE(ICD),'I427') > 0) OR

    (CHAR.INDEX(UPCASE(ICD),'I428') > 0) OR

    (CHAR.INDEX(UPCASE(ICD),'I429') > 0) OR

    (CHAR.INDEX(UPCASE(ICD),'P290') > 0).


      EXECUTE.

   VARIABLE LABELS CCI_2 'Congestive Heart Failure'.

   VALUE LABELS CCI_2 0 'no Congestive Heart Failure' 1 'Congestive Heart Failure'.


*CCI 3.

COMPUTE CCI_3 =

   (CHAR.INDEX(UPCASE(ICD),'I70') > 0) OR

   (CHAR.INDEX(UPCASE(ICD),'I71') > 0) OR

   (CHAR.INDEX(UPCASE(ICD),'I731') > 0) OR

   (CHAR.INDEX(UPCASE(ICD),'I738') > 0) OR

   (CHAR.INDEX(UPCASE(ICD),'I739') > 0) OR

   (CHAR.INDEX(UPCASE(ICD),'I771') > 0) OR
```

```
   (CHAR.INDEX(UPCASE(ICD),'I790') > 0) OR
   (CHAR.INDEX(UPCASE(ICD),'I792') > 0) OR
   (CHAR.INDEX(UPCASE(ICD),'K551') > 0) OR
   (CHAR.INDEX(UPCASE(ICD),'K558') > 0) OR
   (CHAR.INDEX(UPCASE(ICD),'K559') > 0) OR
   (CHAR.INDEX(UPCASE(ICD),'Z958') > 0) OR
   (CHAR.INDEX(UPCASE(ICD),'Z959') > 0).

     EXECUTE.
   VARIABLE LABELS CCI_3 'Peripheral Vascular Disease'.
   VALUE LABELS CCI_3 0 'no Peripheral Vascular Disease' 1 'Peripheral Vascular Disease'.

*CCI 4.
COMPUTE CCI_4 =
   (CHAR.INDEX(UPCASE(ICD),'G45') > 0) OR
   (CHAR.INDEX(UPCASE(ICD),'G46') > 0) OR
   (CHAR.INDEX(UPCASE(ICD),'H340') > 0) OR
   (CHAR.INDEX(UPCASE(ICD),'I6') > 0).

     EXECUTE.
   VARIABLE LABELS CCI_4 'Cerebrovascular Disease'.
   VALUE LABELS CCI_4 0 'no Cerebrovascular Disease' 1 'Cerebrovascular Disease'.


*CCI 5.
COMPUTE CCI_5 =
   (CHAR.INDEX(UPCASE(ICD),'F00') > 0) OR
   (CHAR.INDEX(UPCASE(ICD),'F01') > 0) OR
   (CHAR.INDEX(UPCASE(ICD),'F02') > 0) OR
```

```
    (CHAR.INDEX(UPCASE(ICD),'F03') > 0) OR
    (CHAR.INDEX(UPCASE(ICD),'F051') > 0) OR
    (CHAR.INDEX(UPCASE(ICD),'G30') > 0) OR
    (CHAR.INDEX(UPCASE(ICD),'G311') > 0).

        EXECUTE.
VARIABLE LABELS CCI_5 'Dementia'.
VALUE LABELS CCI_5 0 'no Dementia' 1 'Dementia'.

*CCI 6.
COMPUTE CCI_6 =
    (CHAR.INDEX(UPCASE(ICD),'I278') > 0) OR
    (CHAR.INDEX(UPCASE(ICD),'I279') > 0) OR
    (CHAR.INDEX(UPCASE(ICD),'IJ40') > 0) OR
    (CHAR.INDEX(UPCASE(ICD),'J41') > 0) OR
    (CHAR.INDEX(UPCASE(ICD),'J42') > 0) OR
    (CHAR.INDEX(UPCASE(ICD),'J43') > 0) OR
    (CHAR.INDEX(UPCASE(ICD),'J44') > 0) OR
    (CHAR.INDEX(UPCASE(ICD),'J45') > 0) OR
    (CHAR.INDEX(UPCASE(ICD),'J46') > 0) OR
    (CHAR.INDEX(UPCASE(ICD),'J47') > 0) OR
    (CHAR.INDEX(UPCASE(ICD),'J60') > 0) OR
    (CHAR.INDEX(UPCASE(ICD),'J61') > 0) OR
    (CHAR.INDEX(UPCASE(ICD),'J62') > 0) OR
    (CHAR.INDEX(UPCASE(ICD),'J63') > 0) OR
    (CHAR.INDEX(UPCASE(ICD),'J64') > 0) OR
    (CHAR.INDEX(UPCASE(ICD),'J65') > 0) OR
    (CHAR.INDEX(UPCASE(ICD),'J66') > 0) OR
    (CHAR.INDEX(UPCASE(ICD),'J67') > 0) OR
```

```
   (CHAR.INDEX(UPCASE(ICD),'J684') > 0) OR
   (CHAR.INDEX(UPCASE(ICD),'J701') > 0) OR
   (CHAR.INDEX(UPCASE(ICD),'J703') > 0).

      EXECUTE.
VARIABLE LABELS CCI_6 'Chronic Pulmonary Disease'.
VALUE LABELS CCI_6 0 'no Chronic Pulmonary Disease' 1 'Chronic Pulmonary Disease'.

*CCI 7.
COMPUTE CCI_7 =
   (CHAR.INDEX(UPCASE(ICD),'M05') > 0) OR
   (CHAR.INDEX(UPCASE(ICD),'M06') > 0) OR
   (CHAR.INDEX(UPCASE(ICD),'M315') > 0) OR
   (CHAR.INDEX(UPCASE(ICD),'M32') > 0) OR
   (CHAR.INDEX(UPCASE(ICD),'M33') > 0) OR
   (CHAR.INDEX(UPCASE(ICD),'M34') > 0) OR
   (CHAR.INDEX(UPCASE(ICD),'M351') > 0) OR
   (CHAR.INDEX(UPCASE(ICD),'M353') > 0) OR
   (CHAR.INDEX(UPCASE(ICD),'M360') > 0) OR
   (CHAR.INDEX(UPCASE(ICD),'J47') > 0).

      EXECUTE.
VARIABLE LABELS CCI_7 'Connective Tissue Disease-Rheumatic Disease'.
VALUE LABELS CCI_7 0 'no Connective Tissue Disease-Rheumatic Disease' 1 'Connective Tissue Disease-
Rheumatic Disease'.

*CCI 8.
COMPUTE CCI_8 =
   (CHAR.INDEX(UPCASE(ICD),'K25') > 0) OR
   (CHAR.INDEX(UPCASE(ICD),'K26') > 0) OR
```

```
    (CHAR.INDEX(UPCASE(ICD),'K27') > 0) OR
    (CHAR.INDEX(UPCASE(ICD),'K28') > 0).

      EXECUTE.
VARIABLE LABELS CCI_8 'Peptic Ulcer Disease'.
VALUE LABELS CCI_8 0 'no Peptic Ulcer Disease' 1 'Peptic Ulcer Disease'.

*CCI 9.
COMPUTE CCI_9 = (CHAR.INDEX(UPCASE(ICD),'B18') > 0) OR
    (CHAR.INDEX(UPCASE(ICD),'K700') > 0) OR
    (CHAR.INDEX(UPCASE(ICD),'K701') > 0) OR
    (CHAR.INDEX(UPCASE(ICD),'K702') > 0) OR
    (CHAR.INDEX(UPCASE(ICD),'K703') > 0) OR
    (CHAR.INDEX(UPCASE(ICD),'K709') > 0) OR
    (CHAR.INDEX(UPCASE(ICD),'K713') > 0) OR
    (CHAR.INDEX(UPCASE(ICD),'K714') > 0) OR
    (CHAR.INDEX(UPCASE(ICD),'K715') > 0) OR
    (CHAR.INDEX(UPCASE(ICD),'K717') > 0) OR
    (CHAR.INDEX(UPCASE(ICD),'K73') > 0) OR
    (CHAR.INDEX(UPCASE(ICD),'K74') > 0) OR
    (CHAR.INDEX(UPCASE(ICD),'K760') > 0) OR
    (CHAR.INDEX(UPCASE(ICD),'K762') > 0) OR
    (CHAR.INDEX(UPCASE(ICD),'K763') > 0) OR
    (CHAR.INDEX(UPCASE(ICD),'K764') > 0) OR
    (CHAR.INDEX(UPCASE(ICD),'K768') > 0) OR
    (CHAR.INDEX(UPCASE(ICD),'K768') > 0) OR
    (CHAR.INDEX(UPCASE(ICD),'Z944') > 0).

      EXECUTE.
```

```
VARIABLE LABELS CCI_9 'Mild Liver Disease'.

VALUE LABELS CCI_9 0 'no Mild Liver Disease' 1 'Mild Liver Disease'.


*CCI 10.

COMPUTE CCI_10 =

   (CHAR.INDEX(UPCASE(ICD),'E100') > 0) OR

   (CHAR.INDEX(UPCASE(ICD),'E101') > 0) OR

   (CHAR.INDEX(UPCASE(ICD),'E106') > 0) OR

   (CHAR.INDEX(UPCASE(ICD),'E108') > 0) OR

   (CHAR.INDEX(UPCASE(ICD),'E109') > 0) OR

   (CHAR.INDEX(UPCASE(ICD),'E110') > 0) OR

   (CHAR.INDEX(UPCASE(ICD),'E111') > 0) OR

   (CHAR.INDEX(UPCASE(ICD),'E116') > 0) OR

   (CHAR.INDEX(UPCASE(ICD),'E118') > 0) OR

   (CHAR.INDEX(UPCASE(ICD),'E119') > 0) OR

   (CHAR.INDEX(UPCASE(ICD),'E120') > 0) OR

   (CHAR.INDEX(UPCASE(ICD),'E121') > 0) OR

   (CHAR.INDEX(UPCASE(ICD),'E126') > 0) OR

   (CHAR.INDEX(UPCASE(ICD),'E128') > 0) OR

   (CHAR.INDEX(UPCASE(ICD),'E129') > 0) OR

   (CHAR.INDEX(UPCASE(ICD),'E130') > 0) OR

   (CHAR.INDEX(UPCASE(ICD),'E131') > 0) OR

   (CHAR.INDEX(UPCASE(ICD),'E136') > 0) OR

   (CHAR.INDEX(UPCASE(ICD),'E138') > 0) OR

   (CHAR.INDEX(UPCASE(ICD),'E139') > 0) OR

   (CHAR.INDEX(UPCASE(ICD),'E140') > 0) OR

   (CHAR.INDEX(UPCASE(ICD),'E141') > 0) OR

   (CHAR.INDEX(UPCASE(ICD),'E146') > 0) OR

   (CHAR.INDEX(UPCASE(ICD),'E148') > 0) OR
```

```
   (CHAR.INDEX(UPCASE(ICD),'E149') > 0).

      EXECUTE.
VARIABLE LABELS CCI_10 'Diabetes without complications'.
VALUE LABELS CCI_10 0 'no Diabetes without complications' 1 'Diabetes without complications'.

*CCI 11.
COMPUTE CCI_11 =
   (CHAR.INDEX(UPCASE(ICD),'E102') > 0) OR
   (CHAR.INDEX(UPCASE(ICD),'E103') > 0) OR
   (CHAR.INDEX(UPCASE(ICD),'E104') > 0) OR
   (CHAR.INDEX(UPCASE(ICD),'E105') > 0) OR
   (CHAR.INDEX(UPCASE(ICD),'E107') > 0) OR
   (CHAR.INDEX(UPCASE(ICD),'E112') > 0) OR
   (CHAR.INDEX(UPCASE(ICD),'E113') > 0) OR
   (CHAR.INDEX(UPCASE(ICD),'E114') > 0) OR
   (CHAR.INDEX(UPCASE(ICD),'E115') > 0) OR
   (CHAR.INDEX(UPCASE(ICD),'E117') > 0) OR
   (CHAR.INDEX(UPCASE(ICD),'E122') > 0) OR
   (CHAR.INDEX(UPCASE(ICD),'E123') > 0) OR
   (CHAR.INDEX(UPCASE(ICD),'E124') > 0) OR
   (CHAR.INDEX(UPCASE(ICD),'E125') > 0) OR
   (CHAR.INDEX(UPCASE(ICD),'E127') > 0) OR
   (CHAR.INDEX(UPCASE(ICD),'E132') > 0) OR
   (CHAR.INDEX(UPCASE(ICD),'E133') > 0) OR
   (CHAR.INDEX(UPCASE(ICD),'E134') > 0) OR
   (CHAR.INDEX(UPCASE(ICD),'E135') > 0) OR
   (CHAR.INDEX(UPCASE(ICD),'E137') > 0) OR
   (CHAR.INDEX(UPCASE(ICD),'E142') > 0) OR
```

```
   (CHAR.INDEX(UPCASE(ICD),'E143') > 0) OR
   (CHAR.INDEX(UPCASE(ICD),'E144') > 0) OR
   (CHAR.INDEX(UPCASE(ICD),'E145') > 0) OR
   (CHAR.INDEX(UPCASE(ICD),'E147') > 0).

      EXECUTE.
VARIABLE LABELS CCI_11 'Diabetes with chronic complication'.
VALUE LABELS CCI_11 0 'no Diabetes with chronic complication' 1 'Diabetes with chronic complication'.

*CCI 12.
COMPUTE CCI_12 =
   (CHAR.INDEX(UPCASE(ICD),'G041') > 0) OR
   (CHAR.INDEX(UPCASE(ICD),'G114') > 0) OR
   (CHAR.INDEX(UPCASE(ICD),'G801') > 0) OR
   (CHAR.INDEX(UPCASE(ICD),'G802') > 0) OR
   (CHAR.INDEX(UPCASE(ICD),'G81') > 0) OR
   (CHAR.INDEX(UPCASE(ICD),'G82') > 0) OR
   (CHAR.INDEX(UPCASE(ICD),'G830') > 0) OR
   (CHAR.INDEX(UPCASE(ICD),'G831') > 0) OR
   (CHAR.INDEX(UPCASE(ICD),'G832') > 0) OR
   (CHAR.INDEX(UPCASE(ICD),'G833') > 0) OR
   (CHAR.INDEX(UPCASE(ICD),'G834') > 0) OR
   (CHAR.INDEX(UPCASE(ICD),'G839') > 0).

      EXECUTE.
VARIABLE LABELS CCI_12 'Hemiplegia or paraplegia'.
VALUE LABELS CCI_12 0 'no Hemiplegia or paraplegia' 1 'Hemiplegia or paraplegia'.

*CCI 13.
```

```
COMPUTE CCI_13 =
    (CHAR.INDEX(UPCASE(ICD),'I120') > 0) OR
    (CHAR.INDEX(UPCASE(ICD),'I131') > 0) OR
    (CHAR.INDEX(UPCASE(ICD),'N032') > 0) OR
    (CHAR.INDEX(UPCASE(ICD),'N033') > 0) OR
    (CHAR.INDEX(UPCASE(ICD),'N034') > 0) OR
    (CHAR.INDEX(UPCASE(ICD),'N035') > 0) OR
    (CHAR.INDEX(UPCASE(ICD),'N036') > 0) OR
    (CHAR.INDEX(UPCASE(ICD),'N037') > 0) OR
    (CHAR.INDEX(UPCASE(ICD),'N052') > 0) OR
    (CHAR.INDEX(UPCASE(ICD),'N053') > 0) OR
    (CHAR.INDEX(UPCASE(ICD),'N054') > 0) OR
    (CHAR.INDEX(UPCASE(ICD),'N055') > 0) OR
    (CHAR.INDEX(UPCASE(ICD),'N056') > 0) OR
    (CHAR.INDEX(UPCASE(ICD),'N057') > 0) OR
    (CHAR.INDEX(UPCASE(ICD),'N18') > 0) OR
    (CHAR.INDEX(UPCASE(ICD),'N19') > 0) OR
    (CHAR.INDEX(UPCASE(ICD),'N250') > 0) OR
    (CHAR.INDEX(UPCASE(ICD),'Z490') > 0) OR
    (CHAR.INDEX(UPCASE(ICD),'Z491') > 0) OR
    (CHAR.INDEX(UPCASE(ICD),'Z492') > 0) OR
    (CHAR.INDEX(UPCASE(ICD),'Z940') > 0) OR
    (CHAR.INDEX(UPCASE(ICD),'Z992') > 0).

        EXECUTE.
VARIABLE LABELS CCI_13 'Renal disease'.
VALUE LABELS CCI_13 0 'no Renal disease' 1 'Renal disease'.

*CCI 14.
```

```
COMPUTE CCI_14 =
  (CHAR.INDEX(UPCASE(ICD),'C0') > 0) OR
  (CHAR.INDEX(UPCASE(ICD),'C30') > 0) OR
  (CHAR.INDEX(UPCASE(ICD),'C31') > 0) OR
  (CHAR.INDEX(UPCASE(ICD),'C32') > 0) OR
  (CHAR.INDEX(UPCASE(ICD),'C33') > 0) OR
  (CHAR.INDEX(UPCASE(ICD),'C34') > 0) OR
  (CHAR.INDEX(UPCASE(ICD),'C37') > 0) OR
  (CHAR.INDEX(UPCASE(ICD),'C38') > 0) OR
  (CHAR.INDEX(UPCASE(ICD),'C39') > 0) OR
  (CHAR.INDEX(UPCASE(ICD),'C40') > 0) OR
  (CHAR.INDEX(UPCASE(ICD),'C41') > 0) OR
  (CHAR.INDEX(UPCASE(ICD),'C43') > 0) OR
  (CHAR.INDEX(UPCASE(ICD),'C45') > 0) OR
  (CHAR.INDEX(UPCASE(ICD),'C46') > 0) OR
  (CHAR.INDEX(UPCASE(ICD),'C47') > 0) OR
  (CHAR.INDEX(UPCASE(ICD),'C48') > 0) OR
  (CHAR.INDEX(UPCASE(ICD),'C49') > 0) OR
  (CHAR.INDEX(UPCASE(ICD),'C50') > 0) OR
  (CHAR.INDEX(UPCASE(ICD),'C51') > 0) OR
  (CHAR.INDEX(UPCASE(ICD),'C52') > 0) OR
  (CHAR.INDEX(UPCASE(ICD),'C53') > 0) OR
  (CHAR.INDEX(UPCASE(ICD),'C54') > 0) OR
  (CHAR.INDEX(UPCASE(ICD),'C55') > 0) OR
  (CHAR.INDEX(UPCASE(ICD),'C56') > 0) OR
  (CHAR.INDEX(UPCASE(ICD),'C57') > 0) OR
  (CHAR.INDEX(UPCASE(ICD),'C58') > 0) OR
  (CHAR.INDEX(UPCASE(ICD),'C6') > 0) OR
  (CHAR.INDEX(UPCASE(ICD),'C70') > 0) OR
```

```
(CHAR.INDEX(UPCASE(ICD),'C71') > 0) OR
(CHAR.INDEX(UPCASE(ICD),'C72') > 0) OR
(CHAR.INDEX(UPCASE(ICD),'C73') > 0) OR
(CHAR.INDEX(UPCASE(ICD),'C74') > 0) OR
(CHAR.INDEX(UPCASE(ICD),'C75') > 0) OR
(CHAR.INDEX(UPCASE(ICD),'C76') > 0) OR
(CHAR.INDEX(UPCASE(ICD),'C81') > 0) OR
(CHAR.INDEX(UPCASE(ICD),'C82') > 0) OR
(CHAR.INDEX(UPCASE(ICD),'C83') > 0) OR
(CHAR.INDEX(UPCASE(ICD),'C84') > 0) OR
(CHAR.INDEX(UPCASE(ICD),'C85') > 0) OR
(CHAR.INDEX(UPCASE(ICD),'C88') > 0) OR
(CHAR.INDEX(UPCASE(ICD),'C90') > 0) OR
(CHAR.INDEX(UPCASE(ICD),'C91') > 0) OR
(CHAR.INDEX(UPCASE(ICD),'C92') > 0) OR
(CHAR.INDEX(UPCASE(ICD),'C93') > 0) OR
(CHAR.INDEX(UPCASE(ICD),'C94') > 0) OR
(CHAR.INDEX(UPCASE(ICD),'C95') > 0) OR
(CHAR.INDEX(UPCASE(ICD),'C96') > 0) OR
(CHAR.INDEX(UPCASE(ICD),'C97') > 0).

EXECUTE.
VARIABLE LABELS CCI_14 'Any malignancy, including lymphoma and leukemia, except malignant neoplasm of skin'.
VALUE LABELS CCI_14 0 'no Any malignancy, including lymphoma and leukemia, except malignant neoplasm of skin' 1 'Any malignancy, including lymphoma and leukemia, except malignant neoplasm of skin'.

*CCI 15.
COMPUTE CCI_15 =
(CHAR.INDEX(UPCASE(ICD),'I850') > 0) OR
```

```
   (CHAR.INDEX(UPCASE(ICD),'I859') > 0) OR
   (CHAR.INDEX(UPCASE(ICD),'I864') > 0) OR
   (CHAR.INDEX(UPCASE(ICD),'I982') > 0) OR
   (CHAR.INDEX(UPCASE(ICD),'K704') > 0) OR
   (CHAR.INDEX(UPCASE(ICD),'K711') > 0) OR
   (CHAR.INDEX(UPCASE(ICD),'K721') > 0) OR
   (CHAR.INDEX(UPCASE(ICD),'K729') > 0) OR
   (CHAR.INDEX(UPCASE(ICD),'K765') > 0) OR
   (CHAR.INDEX(UPCASE(ICD),'K766') > 0) OR
   (CHAR.INDEX(UPCASE(ICD),'K767') > 0).

     EXECUTE.
   VARIABLE LABELS CCI_15 'Moderate or severe liver disease'.
   VALUE LABELS CCI_15 0 'no Moderate or severe liver disease' 1 'Moderate or severe liver disease'.

*CCI 16.
COMPUTE CCI_16 =
   (CHAR.INDEX(UPCASE(ICD),'C77') > 0) OR
   (CHAR.INDEX(UPCASE(ICD),'C78') > 0) OR
   (CHAR.INDEX(UPCASE(ICD),'C79') > 0) OR
   (CHAR.INDEX(UPCASE(ICD),'C80')  > 0).

     EXECUTE.
   VARIABLE LABELS CCI_16 'Metastatic solid tumor'.
   VALUE LABELS CCI_16 0 'no Metastatic solid tumor' 1 'Metastatic solid tumor'.

*CCI 17.
COMPUTE CCI_17 =
   (CHAR.INDEX(UPCASE(ICD),'B20') > 0) OR
```

```
    (CHAR.INDEX(UPCASE(ICD),'B21') > 0) OR
    (CHAR.INDEX(UPCASE(ICD),'B22') > 0) OR
    (CHAR.INDEX(UPCASE(ICD),'B24') > 0).

        EXECUTE.
VARIABLE LABELS CCI_17 'AIDS/HIV'.
VALUE LABELS CCI_17 0 'no AIDS/HIV' 1 'AIDS/HIV'.

RECODE CCI_1 to CCI_17 (0=0) (Else = 1).
    EXECUTE.
*Add up CCI.
COMPUTE CCI = 0.
If CCI_1 > 0 CCI = CCI + 0.
If CCI_2 > 0 CCI = CCI + 2.
If CCI_3 > 0 CCI = CCI + 0.
If CCI_4 > 0 CCI = CCI + 0.
If CCI_5 > 0 CCI = CCI + 2.
If CCI_6 > 0 CCI = CCI + 1.
If CCI_7 > 0 CCI = CCI + 1.
If CCI_8 > 0 CCI = CCI + 0.
If CCI_9 > 0 CCI = CCI + 2.
If CCI_10 > 0 CCI = CCI + 0.
If CCI_11 > 0 CCI = CCI + 1.
If CCI_12 > 0 CCI = CCI + 2.
If CCI_13 > 0 CCI = CCI + 1.
If CCI_14 > 0 CCI = CCI + 2.
If CCI_15 > 0 CCI = CCI + 4.
If CCI_16 > 0 CCI = CCI + 6.
If CCI_17 > 0 CCI = CCI + 4.
```

```
    EXECUTE.

*Subtract mild liver and tumor disease from those with severe disease.
IF CCI_9 > 0 and CCI_15 > 0 CCI = CCI - 2.
IF CCI_14 > 0 and CCI_16 > 0 CCI = CCI - 2.
    EXECUTE.

    FREQUENCIES CCI_1 to CCI.

SAVE OUTFILE 'ARDS_CCI.sav'.
ADD FILES FILE*/KEEP ID CCI.
    EXECUTE.

GET FILE 'ARDS_soc.sav'.
DATASET NAME ARDS WINDOW=FRONT.
DATASET ACTIVATE ARDS.


*Hitta komorbiditeterna.
NUMERIC Tid_Diagn_Inkl(f6).
        VARIABLE LABELS Tid_Diagn_Inkl 'Tid från diagnos till inklusion'.
COMPUTE Tid_Diagn_Inkl = datediff(InskrivDatum,UTDATUM,'days').
    EXECUTE.

NUMERIC Tid_Atg_Inkl(f6).
        VARIABLE LABELS Tid_Atg_Inkl 'Tid från åtgärd till inklusion'.
COMPUTE Tid_Atg_Inkl = datediff(InskrivDatum,UTDATUM,'days').
    EXECUTE.
```

*Ta bort åtgärder och diagnoser som inte varit före IVA med Sepsis.

```
SELECT IF Tid_Diagn_Inkl > 0.
  EXECUTE.
```

*Hitta Cytoadministreringar.

```
NUMERIC CytoAdm(f2).
NUMERIC CytoAdm2(f2).
  VARIABLE LABELS CytoAdm 'Cytoadministreringar -åtgärd- sista sex månaderna'.
  VARIABLE LABELS CytoAdm2 'Cytoadministreringar -åtgärd- sista 12 månaderna'.
  VALUE LABELS CytoAdm 0 'No' 1 'Yes'.
  COMPUTE AA = (CHAR.INDEX(UPCASE(Op),'DT107') > 0).
  COMPUTE BB = (CHAR.INDEX(UPCASE(Op),'DT108') > 0).
  COMPUTE CC = (CHAR.INDEX(UPCASE(Op),'DT112') > 0).
  COMPUTE DD = (CHAR.INDEX(UPCASE(Op),'DT116') > 0).
  COMPUTE EE = (CHAR.INDEX(UPCASE(Op),'DT135') > 0).
  IF AA > 0 or  BB > 0 or CC > 0 or DD > 0  or EE > 0 CytoAdm = 1.
    If CytoAdm = 1 CytoAdm2 = 1.
    If Tid_Atg_Inkl > 365 CytoAdm2 = 0.
    IF sysmis(CytoAdm2) CytoAdm2= 0.
  If Tid_Atg_Inkl > 182 CytoAdm = 0.
  IF sysmis(CytoAdm) CytoAdm= 0.
    EXECUTE.
  DELETE VARIABLES AA BB CC DD EE.
    EXECUTE.
```

*Hitta Strålbehandlingar.

```
NUMERIC Radiak(f2).

NUMERIC Radiak2(f2).

 VARIABLE LABELS Radiak 'Strålbehandlingar -åtgärd- sista sex månaderna'.

 VARIABLE LABELS Radiak2 'Strålbehandlingar -åtgärd- sista 12 månaderna'.

 VALUE LABELS Radiak 0 'No' 1 'Yes'.

 COMPUTE AA = (CHAR.INDEX(UPCASE(Op),'DV069') > 0).

 COMPUTE BB = (CHAR.INDEX(UPCASE(Op),'DV070') > 0).

 COMPUTE CC = (CHAR.INDEX(UPCASE(Op),'DV071') > 0).

 COMPUTE DD = (CHAR.INDEX(UPCASE(Op),'DV072') > 0).

 IF AA > 0 or  BB > 0 or CC > 0 or DD > 0 Radiak = 1.

 IF Radiak = 1 Radiak2 = 1.

  If Tid_Atg_Inkl > 365 Radiak2 = 0.

  IF sysmis(Radiak2) Radiak2= 0.

 If Tid_Atg_Inkl > 182 Radiak = 0.

 IF sysmis(Radiak) Radiak= 0.

  EXECUTE.

 DELETE VARIABLES AA BB CC DD.

  EXECUTE.


*Hitta Hemodialys.

NUMERIC HD_Cron(f2).

 VARIABLE LABELS HD_Cron 'Intermittent hemodialys -åtgärd- sista sex månaderna'.

 VALUE LABELS HD_Cron 0 'No' 1 'Yes'.

 COMPUTE AA = (CHAR.INDEX(UPCASE(Op),'DR016') > 0).

 IF AA > 0 HD_Cron = 1.

 If Tid_Atg_Inkl > 182 HD_Cron = 0.

 IF sysmis(HD_Cron) HD_Cron= 0.

  EXECUTE.

 DELETE VARIABLES AA.
```

```
      EXECUTE.


*Hitta
Diagnos/////////////////////////////////////////////////////////////////////////////////////////////////////////////////
/////////////////////. Obs 5 årr!!! fixa datasetet!!!!!.

*OBS för diabetes II, övervikt och HT söks efter lm 5 år tillbaka!!!! Samma för åtärder för  njursvikt - HD PD´och
övervikt.

*Hitta Ischemisk hjärtsjukdom.

NUMERIC IHD(f2).

   VARIABLE LABELS IHD 'Ischemisk hjärtsjukdom'.

   VALUE LABELS IHD 0 'No' 1 'Yes'.

   COMPUTE AA = (CHAR.INDEX(UPCASE(Diagnos),'I20') > 0).

   COMPUTE BB = (CHAR.INDEX(UPCASE(Diagnos),'I21') > 0).

   COMPUTE CC = (CHAR.INDEX(UPCASE(Diagnos),'I22') > 0).

   COMPUTE DD = (CHAR.INDEX(UPCASE(Diagnos),'I23') > 0).

   COMPUTE EE = (CHAR.INDEX(UPCASE(Diagnos),'I24') > 0).

   COMPUTE FF = (CHAR.INDEX(UPCASE(Diagnos),'I25') > 0).

   IF AA > 0 or  BB > 0 or CC > 0 or DD > 0 or EE > 0 or FF > 0 IHD = 1.

   IF sysmis(IHD) IHD= 0.

      EXECUTE.

   DELETE VARIABLES AA BB CC DD EE FF.

      EXECUTE.


*Hitta annan hjärtsjukdom.

NUMERIC OtherHD(f2).

   VARIABLE LABELS OtherHD 'Annan hjärtsjukdom - icke IHD'.

   VALUE LABELS OtherHD 0 'No' 1 'Yes'.

   COMPUTE AA = (CHAR.INDEX(UPCASE(Diagnos),'I3') > 0).

   COMPUTE BB = (CHAR.INDEX(UPCASE(Diagnos),'I4') > 0).

   COMPUTE CC = (CHAR.INDEX(UPCASE(Diagnos),'I5') > 0).
```

```
   COMPUTE DD = (CHAR.INDEX(UPCASE(Diagnos),'I01') > 0).
   COMPUTE EE = (CHAR.INDEX(UPCASE(Diagnos),'I05') > 0).
   COMPUTE FF = (CHAR.INDEX(UPCASE(Diagnos),'I06') > 0).
   COMPUTE GG = (CHAR.INDEX(UPCASE(Diagnos),'I07') > 0).
   COMPUTE HH = (CHAR.INDEX(UPCASE(Diagnos),'I08') > 0).
   COMPUTE II = (CHAR.INDEX(UPCASE(Diagnos),'I09') > 0).
   COMPUTE JJ = (CHAR.INDEX(UPCASE(Diagnos),'I11') > 0).
   COMPUTE KK = (CHAR.INDEX(UPCASE(Diagnos),'I13') > 0).
   COMPUTE LL = (CHAR.INDEX(UPCASE(Diagnos),'I27') > 0).
   IF AA > 0 or  BB > 0 or CC > 0 or DD > 0 or EE > 0 or FF > 0 or GG > 0 or HH > 0 or II > 0 or JJ > 0 or KK > 0 or LL
> 0 OtherHD = 1.
   IF sysmis(OtherHD) OtherHD= 0.
     EXECUTE.
   DELETE VARIABLES AA BB CC DD EE FF GG HH II JJ KK LL.
     EXECUTE.

*Hitta hypertoni.
NUMERIC Hypertens(f2).
   VARIABLE LABELS Hypertens 'Hypertoni'.
   VALUE LABELS Hypertens 0 'No' 1 'Yes'.
   COMPUTE AA = (CHAR.INDEX(UPCASE(Diagnos),'I1') > 0).
   IF AA > 0 Hypertens = 1.
   IF sysmis(Hypertens) Hypertens = 0.
     EXECUTE.
   DELETE VARIABLES AA.
     EXECUTE.

*Hitta Diabetes typ I.
NUMERIC Diabetes1(f2).
   VARIABLE LABELS Diabetes1 'Diabetes typ 1'.
```

```
   VALUE LABELS Diabetes1 0 'No' 1 'Yes'.
   COMPUTE AA = (CHAR.INDEX(UPCASE(Diagnos),'E10') > 0).
   IF AA > 0 Diabetes1 = 1.
   IF sysmis(Diabetes1) Diabetes1 = 0.
      EXECUTE.
   DELETE VARIABLES AA.
      EXECUTE.

*Hitta Diabetes typ II.
NUMERIC Diabetes2(f2).
   VARIABLE LABELS Diabetes2 'Diabetes typ 2'.
   VALUE LABELS Diabetes2 0 'No' 1 'Yes'.
   COMPUTE AA = (CHAR.INDEX(UPCASE(Diagnos),'E11') > 0).
      EXECUTE.
   IF AA > 0 Diabetes2 = 1.
   IF sysmis(Diabetes2) Diabetes2 = 0.
   EXECUTE.
   DELETE VARIABLES AA.
      EXECUTE.

*Hittta stroke.
NUMERIC Stroke(f2).
   VARIABLE LABELS Stroke 'Någon Cerebrovaskulär sjukdom'.
   VALUE LABELS Stroke 0 'No' 1 'Yes'.
   COMPUTE AA = (CHAR.INDEX(UPCASE(Diagnos),'I6') > 0).
   IF AA > 0 Stroke = 1.
   IF sysmis(Stroke) Stroke = 0.
      EXECUTE.
   DELETE VARIABLES AA.
```

```
    EXECUTE.

*Hitta kronisk njursvikt.
NUMERIC Njursvikt(f2).
  VARIABLE LABELS Njursvikt 'Njursvikt'.
  VALUE LABELS Njursvikt 0 'No' 1 'Yes'.
  COMPUTE N181 = (CHAR.INDEX(UPCASE(Diagnos),'N181') > 0).
  COMPUTE N182 = (CHAR.INDEX(UPCASE(Diagnos),'N182') > 0).
  COMPUTE N183 = (CHAR.INDEX(UPCASE(Diagnos),'N183') > 0).
  COMPUTE N184 = (CHAR.INDEX(UPCASE(Diagnos),'N184') > 0).
  COMPUTE N185 = (CHAR.INDEX(UPCASE(Diagnos),'N185') > 0).
  COMPUTE N189 = (CHAR.INDEX(UPCASE(Diagnos),'N189') > 0).
  *Hitta HD, PD senaste 5 åren.
    COMPUTE DR016 = (CHAR.INDEX(UPCASE(Op),'DR016') > 0).
    COMPUTE DR024 = (CHAR.INDEX(UPCASE(Op),'DR024') > 0).
    IF N181 > 0 or  N182 > 0 or  N183 > 0 or  N184 > 0 or  N185 > 0 or  N189 > 0 or  DR016 > 0 or DR024 > 0
Njursvikt = 1.
  IF sysmis(Njursvikt) Njursvikt = 0.
  RECODE N181 to DR024 (SYSMIS = 0).
    EXECUTE.

*Hitta KOL, inklusive emfysem och kronisk bronkit.
NUMERIC KOL(f2).
  VARIABLE LABELS KOL 'KOL, emfysem, kronisk bronkit'.
  VALUE LABELS KOL 0 'No' 1 'Yes'.
  COMPUTE AA = (CHAR.INDEX(UPCASE(Diagnos),'J41') > 0).
  COMPUTE BB = (CHAR.INDEX(UPCASE(Diagnos),'J42') > 0).
  COMPUTE CC = (CHAR.INDEX(UPCASE(Diagnos),'J43') > 0).
  COMPUTE DD = (CHAR.INDEX(UPCASE(Diagnos),'J44') > 0).
  IF AA > 0 or  BB > 0 or CC > 0 or DD > 0 KOL = 1.
```

```
IF sysmis(KOL) KOL= 0.
    EXECUTE.
DELETE VARIABLES AA BB CC DD.
    EXECUTE.

*Hitta Astma.
NUMERIC Astma(f2).
  VARIABLE LABELS Astma 'Astma'.
  VALUE LABELS Astma 0 'No' 1 'Yes'.
  COMPUTE AA = (CHAR.INDEX(UPCASE(Diagnos),'J45') > 0).
  COMPUTE BB = (CHAR.INDEX(UPCASE(Diagnos),'J46') > 0).
  IF AA > 0 or  BB > 0 Astma = 1.
  IF sysmis(Astma) Astma = 0.
    EXECUTE.
  DELETE VARIABLES AA BB.
    EXECUTE.

*Hitta fetma inklusive överviktskirurgi.
NUMERIC Fetma(f2).
  VARIABLE LABELS Fetma 'Fetma'.
  VALUE LABELS Fetma 0 'No' 1 'Yes'.
  COMPUTE AA = (CHAR.INDEX(UPCASE(Diagnos),'E66') > 0).
  *Hitta Överviktskirurgi.
    COMPUTE BB = (CHAR.INDEX(UPCASE(Op),'JDF') > 0).
    COMPUTE CC = (CHAR.INDEX(UPCASE(Op),'JFD03') > 0).
    IF AA > 0 or  BB > 0 or CC > 0 Fetma = 1.
  IF sysmis(Fetma) Fetma = 0.
    EXECUTE.
  DELETE VARIABLES AA BB CC.
```

```
    EXECUTE.

*Hitta Immunsupprimerande sjukdom eller efter åtgärd.
NUMERIC Immunsupprimerad(f2).
  VARIABLE LABELS Immunsupprimerad 'Immunsupprimerad'.
  VALUE LABELS Immunsupprimerad 0 'No' 1 'Yes'.
  COMPUTE AA = (CHAR.INDEX(UPCASE(Diagnos),'D71') > 0).
  COMPUTE BB = (CHAR.INDEX(UPCASE(Diagnos),'D80') > 0).
  COMPUTE CC = (CHAR.INDEX(UPCASE(Diagnos),'D81') > 0).
  COMPUTE DD = (CHAR.INDEX(UPCASE(Diagnos),'D82') > 0).
  COMPUTE EE = (CHAR.INDEX(UPCASE(Diagnos),'D83') > 0).
  COMPUTE FF = (CHAR.INDEX(UPCASE(Diagnos),'D84') > 0).
  COMPUTE GG = (CHAR.INDEX(UPCASE(Diagnos),'D89') > 0).
  IF AA > 0 or  BB > 0 or CC > 0 or DD > 0 or EE > 0 or FF > 0 or GG > 0  Immunsupprimerad = 1.
  *Hitta immunsupprimerande åtgärder lm senaste 6 månaderna.
  IF CytoAdm = 1 or Radiak = 1 Immunsupprimerad = 1.
  IF sysmis(Immunsupprimerad)  Immunsupprimerad = 0.
    EXECUTE.
  DELETE VARIABLES AA BB CC DD EE FF GG.
    EXECUTE.

*Hitta aktiv solid tumör senaste året dvs diagnossatt Senaste 12 mån..
NUMERIC Cancer_dia(f2).
NUMERIC CancerDiagn(f2).
  VARIABLE LABELS Cancer_dia 'Cancer'.
  VALUE LABELS Cancer_dia 0 'No' 1 'Yes'.
  COMPUTE AA = (CHAR.INDEX(UPCASE(Diagnos),'C0') > 0).
  COMPUTE BB = (CHAR.INDEX(UPCASE(Diagnos),'C1') > 0).
  COMPUTE CC = (CHAR.INDEX(UPCASE(Diagnos),'C2') > 0).
```

```
   COMPUTE DD = (CHAR.INDEX(UPCASE(Diagnos),'C3') > 0).
   COMPUTE EE = (CHAR.INDEX(UPCASE(Diagnos),'C4') > 0).
   COMPUTE FF = (CHAR.INDEX(UPCASE(Diagnos),'C5') > 0).
   COMPUTE GG = (CHAR.INDEX(UPCASE(Diagnos),'C6') > 0).
   COMPUTE HH = (CHAR.INDEX(UPCASE(Diagnos),'C7') > 0).
   COMPUTE II = (CHAR.INDEX(UPCASE(Diagnos),'C80') > 0).
   COMPUTE JJ = (CHAR.INDEX(UPCASE(Diagnos),'C97') > 0).
    IF AA > 0 or  BB > 0 or CC > 0 or DD > 0 or EE > 0 or FF > 0 or GG > 0 or HH > 0 or II > 0 or JJ > 0 CancerDiagn =
1.
   IF Tid_Diagn_Inkl > 365 CancerDiagn = 0.
   If CancerDiagn = 1 Cancer_dia = 1.
   IF sysmis(Cancer_dia) Cancer_dia = 0.
      EXECUTE.
   DELETE VARIABLES AA to JJ CancerDiagn.
      EXECUTE.


*Hitta aktiv blodcancer senaste året dvs diagnossatt Senaste 12 mån..
NUMERIC BlodCancer(f2).
NUMERIC BlodCancerDiagn(f2).
   VARIABLE LABELS BlodCancer 'Hematologisk malignitet'.
   VALUE LABELS BlodCancer 0 'No' 1 'Yes'.
   COMPUTE AA = (CHAR.INDEX(UPCASE(Diagnos),'C81') > 0).
   COMPUTE BB = (CHAR.INDEX(UPCASE(Diagnos),'C82') > 0).
   COMPUTE CC = (CHAR.INDEX(UPCASE(Diagnos),'C83') > 0).
   COMPUTE DD = (CHAR.INDEX(UPCASE(Diagnos),'C84') > 0).
   COMPUTE EE = (CHAR.INDEX(UPCASE(Diagnos),'C85') > 0).
   COMPUTE FF = (CHAR.INDEX(UPCASE(Diagnos),'C86') > 0).
   COMPUTE GG = (CHAR.INDEX(UPCASE(Diagnos),'C88') > 0).
   COMPUTE HH = (CHAR.INDEX(UPCASE(Diagnos),'C90') > 0).
   COMPUTE II = (CHAR.INDEX(UPCASE(Diagnos),'C91') > 0).
```

```
   COMPUTE JJ = (CHAR.INDEX(UPCASE(Diagnos),'C92') > 0).
   COMPUTE KK = (CHAR.INDEX(UPCASE(Diagnos),'C93') > 0).
   COMPUTE LL = (CHAR.INDEX(UPCASE(Diagnos),'C94') > 0).
   COMPUTE MM = (CHAR.INDEX(UPCASE(Diagnos),'C95') > 0).
   COMPUTE NN = (CHAR.INDEX(UPCASE(Diagnos),'C96') > 0).
    IF AA > 0 or  BB > 0 or CC > 0 or DD > 0 or EE > 0 or FF > 0 or GG > 0 or HH > 0 or II > 0 or JJ > 0 or KK > 0 or LL
> 0 or MM > 0 or NN > 0 BlodCancerDiagn = 1.
   IF Tid_Diagn_Inkl > 365 BlodCancerDiagn = 0.
   If BlodCancerDiagn = 1 BlodCancer = 1.
   IF sysmis(BlodCancer) BlodCancer= 0.
      EXECUTE.
   DELETE VARIABLES AA to NN BlodCancerDiagn.
      EXECUTE.

*Hitta inflammatorisk sjukdom.
NUMERIC Inflam(f2).
   VARIABLE LABELS Inflam 'Systeminflammatorisk sjukdom'.
   VALUE LABELS Inflam 0 'No' 1 'Yes'.
   COMPUTE AA = (CHAR.INDEX(UPCASE(Diagnos),'M3') > 0).
   COMPUTE BB = (CHAR.INDEX(UPCASE(Diagnos),'M05') > 0).
   COMPUTE CC = (CHAR.INDEX(UPCASE(Diagnos),'M06') > 0).
   COMPUTE DD = (CHAR.INDEX(UPCASE(Diagnos),'M07') > 0).
   COMPUTE EE = (CHAR.INDEX(UPCASE(Diagnos),'M08') > 0).
   COMPUTE FF = (CHAR.INDEX(UPCASE(Diagnos),'M09') > 0).
   COMPUTE GG = (CHAR.INDEX(UPCASE(Diagnos),'M10') > 0).
   COMPUTE HH = (CHAR.INDEX(UPCASE(Diagnos),'M11') > 0).
   COMPUTE II = (CHAR.INDEX(UPCASE(Diagnos),'M12') > 0).
   COMPUTE JJ = (CHAR.INDEX(UPCASE(Diagnos),'M13') > 0).
   COMPUTE KK = (CHAR.INDEX(UPCASE(Diagnos),'M14') > 0).
```

```
   IF AA > 0 or  BB > 0 or CC > 0 or DD > 0 or EE > 0 or FF > 0 or GG > 0 or HH > 0 or II > 0 or JJ > 0 or KK > 0 Inflam = 1.
   IF sysmis(Inflam) Inflam= 0.
      EXECUTE.
   DELETE VARIABLES AA BB CC DD EE FF GG HH II JJ KK.
      EXECUTE.
*Hitta transplanterade.
NUMERIC Transplanterad(f2).
   VARIABLE LABELS Transplanterad 'Transplanterad'.
   VALUE LABELS Transplanterad 0 'No' 1 'Yes'.
   COMPUTE AA = (CHAR.INDEX(UPCASE(Diagnos),'Z940') > 0).
   COMPUTE BB = (CHAR.INDEX(UPCASE(Diagnos),'Z941') > 0).
   COMPUTE CC = (CHAR.INDEX(UPCASE(Diagnos),'Z942') > 0).
   COMPUTE DD = (CHAR.INDEX(UPCASE(Diagnos),'Z943') > 0).
   COMPUTE EE = (CHAR.INDEX(UPCASE(Diagnos),'Z944') > 0).

   IF AA > 0 or  BB > 0 or CC > 0 or DD > 0 or EE > 0 Transplanterad = 1.
   IF sysmis(Transplanterad)  Transplanterad = 0.
      EXECUTE.
   DELETE VARIABLES AA BB CC DD EE.
      EXECUTE.

DELETE VARIABLES CounterVTF.
   EXECUTE.
SORT CASES ID(a) INDATUM(a) UTDATUM(a).
   NUMERIC CounterVTF(f3).
   IF $casenum = 1 or ID ne lag(ID) CounterVTF = 1.
   IF ID = lag(ID) CounterVTF = lag(CounterVTF) + 1.
      EXECUTE.
SORT CASES ID(a) CounterVTF(d).
```

EXECUTE.

*Skapa en rad per individ.

If ID = lag(ID) CytoAdm = CytoAdm + lag(CytoAdm).

IF CytoAdm > 0 CytoAdm = 1.

If ID = lag(ID) CytoAdm2 = CytoAdm2 + lag(CytoAdm2).

IF CytoAdm2 > 0 CytoAdm2 = 1.

If ID = lag(ID) Radiak = Radiak + lag(Radiak).

IF Radiak > 0 Radiak = 1.

If ID = lag(ID) Radiak2 = Radiak2 + lag(Radiak2).

IF Radiak2 > 0 Radiak2 = 1.

If ID = lag(ID) HD_Cron = HD_Cron + lag(HD_Cron).

IF HD_Cron > 0 HD_Cron = 1.

If ID = lag(ID) IHD = IHD + lag(IHD).

IF IHD > 0 IHD = 1.

If ID = lag(ID) OtherHD = OtherHD + lag(OtherHD).

IF OtherHD > 0 OtherHD = 1.

If ID = lag(ID) Hypertens = Hypertens + lag(Hypertens).

IF Hypertens > 0 Hypertens = 1.

If ID = lag(ID) Diabetes1 = Diabetes1 + lag(Diabetes1).

IF Diabetes1 > 0 Diabetes1 = 1.

If ID = lag(ID) Diabetes2 = Diabetes2 + lag(Diabetes2).

IF Diabetes2 > 0 Diabetes2 = 1.

If ID = lag(ID) Stroke = Stroke + lag(Stroke).

IF Stroke > 0 Stroke = 1.

If ID = lag(ID) Njursvikt = Njursvikt + lag(Njursvikt).

IF Njursvikt > 0 Njursvikt = 1.

If ID = lag(ID) N181 = N181 + lag(N181).

IF N181 > 0 N181 = 1.

If ID = lag(ID) N182 = N182 + lag(N182).

```
IF N182 > 0 N182 = 1.

If ID = lag(ID) N183 = N183 + lag(N183).

IF N183 > 0 N183 = 1.

If ID = lag(ID) N184 = N184 + lag(N184).

IF N184 > 0 N184 = 1.

If ID = lag(ID) N185 = N185 + lag(N185).

IF N185 > 0 N185 = 1.

If ID = lag(ID) N189 = N189 + lag(N189).

IF N189 > 0 N189 = 1.

If ID = lag(ID) DR016 = DR016 + lag(DR016).

IF DR016 > 0 DR016 = 1.

If ID = lag(ID) DR024 = DR024 + lag(DR024).

IF DR024 > 0 DR024 = 1.

If ID = lag(ID) KOL = KOL + lag(KOL).

IF KOL > 0 KOL = 1.

If ID = lag(ID) Astma = Astma + lag(Astma).

IF Astma > 0 Astma = 1.

If ID = lag(ID) Fetma = Fetma + lag(Fetma).

IF Fetma > 0 Fetma = 1.

If ID = lag(ID) Immunsupprimerad = Immunsupprimerad + lag(Immunsupprimerad).

IF Immunsupprimerad > 0 Immunsupprimerad = 1.

If ID = lag(ID) Cancer_dia = Cancer_dia + lag(Cancer_dia).

IF Cancer_dia > 0 Cancer_dia = 1.

If ID = lag(ID) BlodCancer = BlodCancer + lag(BlodCancer).

IF BlodCancer > 0 BlodCancer = 1.

If ID = lag(ID) Inflam = Inflam + lag(Inflam).

IF Inflam > 0 Inflam = 1.

If ID = lag(ID) Transplanterad = Transplanterad + lag(Transplanterad).

IF Transplanterad > 0 Transplanterad = 1.
```

```
*Spara ett vtf per individ.
  EXECUTE.
SELECT IF CounterVTF = 1.
  EXECUTE.

ADD FILES FILE*
  /KEEP ID CytoAdm to Transplanterad.
    EXECUTE.

SAVE OUTFILE 'ARDS_Comorb.sav'.
  DATASET NAME ARDS_Comorb.
GET FILE 'ARDS_temp.sav'.
  DATASET NAME SIR WINDOW=FRONT.

DATASET ACTIVATE SIR.
MATCH FILES /FILE=*
 /FILE='ARDS_Comorb'
 /BY ID.
EXECUTE.

DATASET ACTIVATE SIR.
MATCH FILES /FILE=*
 /TABLE='ARDS_CCI'
 /BY ID.
EXECUTE.
  DATASET CLOSE ARDS_CCI.
  DATASET CLOSE ARDS_Comorb.
```

```
   DATASET ACTIVATE SIR.


RECODE CytoAdm to CCI (SYSMIS = 0) (ELSE = COPY).
   EXECUTE.
SORT CASES ID(a).


NUMERIC Sepsis(f2)
NUMERIC COVID(f2).
NUMERIC COVID2(f2).
      VALUE LABELS ARDS 0 'No ARDS' 1 'ARDS'.
      VALUE LABELS Sepsis 0 'No Sepsis' 1 'Sepsis'.
      VALUE LABELS Covid 0 'No Covid' 1 'Covid'.
      VARIABLE LABELS COVID2 'Covid också'.
   COMPUTE ARDS = 1.
   COMPUTE Sepsis = 0.
   COMPUTE COVID = 0.
   COMPUTE COVID2 = 0.
      EXECUTE.
SAVE OUTFILE 'TEMP12.sav'.
SORT CASES ID.


Get file 'Y:\Original_SPSS\ut_par_sv_8996_2017.sav'.
DATASET NAME Soc WINDOW=FRONT.
DATASET ACTIVATE Soc.


RENAME VARIABLES (lopnr LKF Kon = ID L Kon).
   FORMATS ID(f10).
      EXECUTE.
```

```
STRING LKF(a10).
COMPUTE LKF = concat(rtrim(LK),' ',rtrim(L)).
  EXECUTE.
  DELETE VARIABLES LK L.
    EXECUTE.
SORT CASES ID(a) INDATUM(a) UTDATUM(a).
  NUMERIC CounterVTF(f3).
  IF $casenum = 1 or ID ne lag(ID) CounterVTF = 1.
  IF ID = lag(ID) CounterVTF = lag(CounterVTF) + 1.
    EXECUTE.
SELECT IF CounterVTF = 1.
EXECUTE.

ADD FILES FILE*
  /KEEP ID SENUTV.
    EXECUTE.
DATASET ACTIVATE SIR.
MATCH FILES /FILE=*
 /FILE='Soc'
 /BY ID.
EXECUTE.

SELECT IF ARDS = 1.
EXECUTE.

  DATASET NAME ARDS.
  DATASET CLOSE Soc.
  DATASET ACTIVATE ARDS.
```

```
SELECT IF InskrTid >0.
   EXECUTE.
COMPUTE ID_ARDS = ID.
   EXECUTE.
COMPUTE ID = $casenum + 300000.
   EXECUTE.

ADD FILES FILE*
/KEEP ID Alder Kon InskrivDatum UtskrivDatum SjukhusTyp Opererad SAPS3Score SAPS3_u_comorb SAPS3ScoreBoxIII
Operationstyp APACHE_Score APACHE_AkutFysPoang IMV_tid CRRT_tid  IMV CRRT ICU_LoS
CytoAdm CytoAdm2 Radiak Radiak2 HD_Cron IHD OtherHD Hypertens Diabetes1 Diabetes2 Stroke Njursvikt N181 N182 N183 N184 N185
N189 DR016 DR024 KOL Astma Fetma Immunsupprimerad Cancer_dia BlodCancer Inflam Transplanterad
CCI COVID COVID2 Sepsis ARDS DeadICU_n DeathDate_ICU Dead_n DeathDate SENUTV ID_ARDS.
   EXECUTE.

*Ensa dataseten.
DATASET ACTIVATE ARDS.

RENAME VARIABLES (InskrivDatum Alder = InskrDat Age).
   EXECUTE.
NUMERIC Sex(f1).
   VARIABLE LABELS Sex 'Sex at ICU admission'.
   VALUE LABELS Sex 1 'Man' 2 'Kvinna'.
   IF Kon = 'M' Sex = 1.
   IF Kon = 'K' Sex = 2.
FORMATS Age(f3).
   VARIABLE LABELS Age 'Age at ICU admission'.
IF Opererad = 'Ja-elektivt' Opererad = 'Ja-elek'.
```

```
IF Opererad = 'Ja-akut' Opererad = 'Ja-akut'.

ALTER TYPE Opererad(a7).

    EXECUTE.


DESCRIPTIVES ID.

SELECT IF Age > 17.

DESCRIPTIVES ID.

FREQUENCIES COVID COVID2 Sepsis ARDS.

SAVE OUTFILE 'ARDS_komplett.sav'.


DATASET CLOSE all.

* Encoding: UTF-8.

*COVID skapas i Skapa_COVIDrev.sps.

DATASET CLOSE all.

*Montera SIR_data_05_17_fr_Gunnar_ickeBin på X.


cd 'Z:/'.

*Obs filen ligger på X!.

*GET DATA

  /TYPE=XLSX

  /FILE='X:\SIR-data_05_17_fr_Gunanr_ickeBin.xlsx'

  /SHEET=name 'SIR-data'

  /CELLRANGE=FULL

  /READNAMES=ON

  /TRAILINGSPACES IGNORE=YES

  /DATATYPEMIN PERCENTAGE=95.0

  /HIDDEN IGNORE=YES.

*EXECUTE.

*DATASET NAME SIR WINDOW=FRONT.
```

```
SAVE OUTFILE 'SIR_start.sav'.

DATASET CLOSE all.
GET FILE 'SIR_start.sav'.
DATASET NAME SIR WINDOW=FRONT.
DATASET ACTIVATE SIR.

RENAME VARIABLES (LÃ¶pNr_Pnr LÃ¶pNr_VTF AvlidenPÃ¥IVA = ID LpnrVTF Avliden_IVA).

FORMATS InskrivDatum(date11).
  FORMATS UtskrivDatum(date11).
  FORMATS AvregDatum(date11).
  FORMATS ID(f10).
  FORMATS LpnrVTF(f10).
  FORMATS Inskr_Ar(f4).
  FORMATS Alder(f4).
  FORMATS CancerTerapi(f2).
    EXECUTE.

*Slå ihop alla ICD-variabler.
STRING IVA_diagnos(A230).
Compute IVA_Diagnos = concat(rtrim(@1ICD10),' ',rtrim(@2ICD10),' ',rtrim(@3ICD10),' ',rtrim(@4ICD10),' ',rtrim(@5ICD10),' ',rtrim(@6ICD10),' ',rtrim(@7ICD10),
' ',rtrim(@8ICD10),' ',rtrim(@9ICD10),' ',rtrim(@10ICD10),' ',rtrim(@11ICD10),' ',rtrim(@12ICD10),' ',rtrim(@13ICD10),' ',rtrim(@14ICD10),' ',rtrim(@15ICD10),' ',rtrim(@16ICD10),
' ',rtrim(@17ICD10),' ',rtrim(@18ICD10),' ',rtrim(@19ICD10),' ',rtrim(@20ICD10),' ',rtrim(@21ICD10),' ',rtrim(@22ICD10),' ',rtrim(@23ICD10),' ',rtrim(@24ICD10),' ',rtrim(@25ICD10),
' ',rtrim(@26ICD10),' ',rtrim(@27ICD10),' ',rtrim(@28ICD10)).
```

```
EXECUTE.

*Undersök bredden (221).
compute count_IVA_Diagnos=char.length(IVA_Diagnos).
EXECUTE.
FREQUENCIES VARIABLES=count_IVA_Diagnos
  /STATISTICS=MINIMUM MAXIMUM MEAN
  /ORDER=ANALYSIS.
DELETE VARIABLES count_IVA_Diagnos @1ICD10 to @28ICD10.
   EXECUTE.

*Skapa SAPS3 utan ålder och comorb..
NUMERIC SAPS3_TEMP(f4).
   RECODE Alder  (1 thru 39 = 0) (40 thru 59 = 3) (60 thru 69 = 9) (70 thru 74 = 13) (75 thru 79 = 15) (80 thru hi = 18) INTO SAPS3_TEMP.
   EXECUTE.
   IF CancerTerapi = 2  SAPS3_TEMP =  SAPS3_TEMP + 3.
   IF KronHjartsvikt = 2 SAPS3_TEMP =  SAPS3_TEMP + 6.
   IF Blodmalignitet = 2 SAPS3_TEMP =  SAPS3_TEMP + 6.
   IF Cirrhos = 2 SAPS3_TEMP =  SAPS3_TEMP + 9.
   IF AIDS  = 2 SAPS3_TEMP =  SAPS3_TEMP + 9.
   IF Cancer = 2 SAPS3_TEMP =  SAPS3_TEMP + 9.
NUMERIC SAPS3_u_comorb(f4).
   COMPUTE  SAPS3_u_comorb = SAPS3Score - SAPS3_TEMP.
   EXECUTE.


SORT CASES LpnrVTF(a).
*Lägg till Inskrivnings TID.
```

```
*Hämta Tider_ML_krypt från servern. Lägg i Z. Ta bort skrivskydd.

GET file 'Y:\Original_SPSS\KonsekvenserAvKritiskSjukdom_ML_Tider.sav'.

DATASET NAME Tider WINDOW=FRONT.

DATASET ACTIVATE Tider.

DELETE VARIABLES L�pNr_Pnr.

  EXECUTE.

RENAME VARIABLES (L�pNr_VTF = LpnrVTF).

  EXECUTE.

  FORMATS LpnrVTF(f10).


SORT CASES LpnrVTF(a).


DATASET ACTIVATE SIR.

MATCH FILES /FILE=*

 /FILE='Tider'

 /BY LpnrVTF.

    EXECUTE.

  DATASET CLOSE Tider.

  DATASET ACTIVATE SIR.


*Lägg till ventilatorbehandling.

Get file 'Y:\Original_SPSS\Ventilator.sav'.

DATASET NAME Vent.

DATASET ACTIVATE Vent.

  RENAME VARIABLES (L�pNr_VTF CRRT AtgardTidMinuter= LpnrVTF IMV IMV_tid).

    EXECUTE.

  FORMATS LpnrVTF(f10).

 SORT CASES LpnrVTF(a) StartTidpunkt(a).

  NUMERIC Counter(f3).
```

```
   IF $casenum =1 or LpnrVTF ne lag(LpnrVTF) Counter = 1.
   IF LpnrVTF = lag(LpnrVTF) Counter = lag(Counter) + 1.
      EXECUTE.


*Lägg ihop mot rad 1 per vtf.
SORT CASES LpnrVTF(a) Counter(d).
IF LpnrVTF = lag(LpnrVTF) IMV_tid = IMV_tid + lag(IMV_tid).
     EXECUTE.
SELECT IF Counter = 1.
   EXECUTE.
ADD FILES FILE*
   /KEEP LpnrVTF IMV_tid.
   EXECUTE.

SORT CASES LpnrVTF(a).

DATASET ACTIVATE SIR.
MATCH FILES /FILE=*
  /FILE='Vent'
  /BY LpnrVTF.
      EXECUTE.
   DATASET CLOSE Vent.
   DATASET ACTIVATE SIR.

*Lägg till dialysbehandling.
Get file 'Y:\Original_SPSS\CRRT.sav'.
DATASET NAME C.
DATASET ACTIVATE C.
```

```
   RENAME VARIABLES (L�pNr_VTF AtgardTidMinuter = LpnrVTF CRRT_tid).
      EXECUTE.
VARIABLE LABELS CRRT_tid 'CRRT_tid i minuter'.
   EXECUTE.
   FORMATS LpnrVTF(f10).

  SORT CASES LpnrVTF(a) StartTidpunkt(a).
   NUMERIC Counter(f3).
   IF $casenum =1 or LpnrVTF ne lag(LpnrVTF) Counter = 1.
   IF LpnrVTF = lag(LpnrVTF) Counter = lag(Counter) + 1.
      EXECUTE.

*Lägg ihop mot rad 1 per vtf.
SORT CASES LpnrVTF(a) Counter(d).
IF LpnrVTF = lag(LpnrVTF) CRRT_tid = CRRT_tid + lag(CRRT_tid).
     EXECUTE.
SELECT IF Counter = 1.
   EXECUTE.
ADD FILES FILE*
   /KEEP LpnrVTF CRRT_tid.
   EXECUTE.
SORT CASES LpnrVTF(a).

DATASET ACTIVATE SIR.
MATCH FILES /FILE=*
  /FILE='C'
  /BY LpnrVTF.
      EXECUTE.
   DATASET CLOSE C.
```

```
   DATASET ACTIVATE SIR.


*Ta bort OptOutade 3 st.
DESCRIPTIVES ID.
SELECT IF InskrTid > 0.
   EXECUTE.
DESCRIPTIVES ID.
SELECT IF InskrivDatum > 0.
   EXECUTE.
DESCRIPTIVES ID.


DELETE VARIABLES InskrivDatum UtskrivDatum.
   EXECUTE.
compute day = xdate.mday(InskrTid).
compute month = xdate.month(InskrTid).
compute year = xdate.year(InskrTid).
compute InskrivDatum = date.dmy(day,month,year).
formats InskrivDatum(date11).
   EXECUTE.
compute day = xdate.mday(UtskrTid).
compute month = xdate.month(UtskrTid).
compute year = xdate.year(UtskrTid).
compute UtskrivDatum = date.dmy(day,month,year).
formats UtskrivDatum(date11).
   EXECUTE.




SORT CASES ID(a) InskrTid(a) Utskrtid(a).
```

```
NUMERIC CounterIVA(f3).
   IF $casenum = 1 or ID ne lag(ID) CounterIVA = 1.
   IF ID = lag(ID) CounterIVA = lag(CounterIVA) + 1.
   EXECUTE.
FREQUENCIES CounterIVA Inskr_Ar.

*Ta bort vårdtillfällen före 2011.
SELECT IF (InskrivDatum > DATE.DMY(31,12,2010)).
   EXECUTE.

DELETE VARIABLES CounterIVA.
   EXECUTE.
SORT CASES ID(a) InskrTid(a) Utskrtid(a).
NUMERIC CounterIVA(f3).
   IF $casenum = 1 or ID ne lag(ID) CounterIVA = 1.
   IF ID = lag(ID) CounterIVA = lag(CounterIVA) + 1.
   EXECUTE.
FREQUENCIES CounterIVA.

*Ta bort vårdtillfällen efter 2016-12-31.
SELECT IF (InskrivDatum < DATE.DMY(01,01,2017)).
   EXECUTE.
DELETE VARIABLES CounterIVA.
   EXECUTE.
SORT CASES ID(a) InskrTid(a) Utskrtid(a).
NUMERIC CounterIVA(f3).
   IF $casenum = 1 or ID ne lag(ID) CounterIVA = 1.
   IF ID = lag(ID) CounterIVA = lag(CounterIVA) + 1.
   EXECUTE.
```

```
FREQUENCIES CounterIVA.


ADD FILES FILE*
   /KEEP LpnrVTF ID InskrTid UtskrTid InskrivDatum UtskrivDatum Avliden AvregDatum Avliden_IVA SjukhusTyp Alder Kon Opererad
   SAPS3Score SAPS3ScoreBoxIII Operationstyp APACHE_Score APACHE_AkutFysPoang Status
   IVA_diagnos SAPS3_TEMP SAPS3_u_comorb IMV_tid CRRT_tid CounterIVA.
      EXECUTE.
*Sepsis.
   COMPUTE BB = (CHAR.INDEX(UPCASE(IVA_diagnos),'R651') > 0).
   COMPUTE CC = (CHAR.INDEX(UPCASE(IVA_diagnos),'R572') > 0).
   COMPUTE AA = (CHAR.INDEX(UPCASE(IVA_diagnos),'A419') > 0).
      EXECUTE.
NUMERIC Sepsis_grad(f2).
   VALUE LABELS Sepsis_grad 0 'Ingen sepsis' 1 'Severe sepsis' 2 'Septic shock' 5 'Unspecified Sepsis'.
   IF AA > 0 Sepsis_grad = 5.
   IF BB > 0 Sepsis_grad = 1.
   IF CC > 0 Sepsis_grad = 2.
   IF sysmis(Sepsis_grad) Sepsis_grad = 0.
      EXECUTE.


NUMERIC Sepsis(f2).
   IF AA > 0 or BB > 0 or CC> 0 Sepsis = 1.
   IF sysmis(Sepsis) Sepsis = 0.
      EXECUTE.
FREQUENCIES Sepsis.


   DELETE VARIABLES AA BB CC.
      EXECUTE.
```

```
*ARDS.
NUMERIC ARDS(f2).
 VARIABLE LABELS ARDS 'ARDS'.
 COMPUTE ARDS = (CHAR.INDEX(UPCASE(IVA_diagnos),'J809') > 0).
 IF sysmis(ARDS) ARDS = 0.
  EXECUTE.
FREQUENCIES ARDS.
*Markera sepsisvårdtillfälle + alla efterföljande.
NUMERIC S(f2).
 VARIABLE LABELS S 'vårdtillfälle med sepsis eller efter sepsisvårdtillfälle'.
If Sepsis = 1 S = 1.
IF ID = lag(ID) and lag(S) = 1 S = 1.

*Markera ARDS-vårdtillfälle + alla efterföljande.
NUMERIC A(f2).
 VARIABLE LABELS A 'vårdtillfälle med ARDS eller efter ARDS-vårdtillfälle'.
If ARDS = 1 A = 1.
IF ID = lag(ID) and lag(A) = 1 A = 1.
 EXECUTE.


SAVE OUTFILE 'SIR_temp2.sav'.
 DATASET CLOSE all.
GET FILE 'SIR_temp2.sav'.
DATASET NAME Sepsis WINDOW=FRONT.
DATASET ACTIVATE Sepsis.

DELETE VARIABLES CounterIVA.
 EXECUTE.
```

```
SORT CASES ID(a) InskrTid(a) Utskrtid(a).
NUMERIC CounterIVA(f3).
   IF $casenum = 1 or ID ne lag(ID) CounterIVA = 1.
   IF ID = lag(ID) CounterIVA = lag(CounterIVA) + 1.
   EXECUTE.
FREQUENCIES CounterIVA.


*SEPSIS!. Sortera bort IVA före sepsis och ej sepsis.
SELECT IF S = 1.
EXECUTE.
DELETE VARIABLES CounterIVA.
   EXECUTE.
SORT CASES ID(a) InskrTid(a) Utskrtid(a).
NUMERIC CounterIVA(f3).
   IF $casenum = 1 or ID ne lag(ID) CounterIVA = 1.
   IF ID = lag(ID) CounterIVA = lag(CounterIVA) + 1.
   EXECUTE.
FREQUENCIES CounterIVA.
*diff är dagar mellan intensivvårdstillfällena. 0 eller 1 dags skillnad innebär samma vtf.
Compute diff = datediff(InskrTid,lag(UtskrTid),'hours').
Compute diff = Diff / 24.
   VARIABLE LABELS diff ' days from out previous to in actual ICU episode'.
   IF ID ne lag(ID) diff = $sysmis.
   IF CounterIVA = 1 X = 1.
   IF lag(X) = 1 and diff < 1 X = 1.
      EXECUTE.
SELECT IF X = 1.
   EXECUTE.
FREQUENCIES CounterIVA.
```

```
*Skapa och spara ett vtf.

DELETE VARIABLES CounterIVA.

   EXECUTE.

SORT CASES ID(a) InskrTid(a) UtskrTid(a).

NUMERIC CounterIVA(f3).

   IF $casenum = 1 or ID ne lag(ID) CounterIVA = 1.

   IF ID = lag(ID) CounterIVA = lag(CounterIVA) + 1.

   EXECUTE.

*Åtgärder.


*Lägg ihop mot första vårdtillfället.


SORT CASES ID(a) CounterIVA(d).

   EXECUTE.

IF ID = lag(ID) CRRT_tid = CRRT_tid + lag(CRRT_tid).

IF ID = lag(ID) IMV_tid = IMV_tid + lag(IMV_tid).

IF ID = lag(ID) and lag(Avliden_IVA) = 'Ja' Avliden_IVA = 'Ja'.

IF ID = lag(ID) UtskrivDatum = lag(UtskrivDatum).

IF ID = lag(ID) UtskrTid = lag(UtskrTid).

IF ID = lag(ID) and lag(Sepsis_grad) = 1 Sepsis_grad = 1.

IF ID = lag(ID) and lag(Sepsis_grad) = 2 Sepsis_grad = 2.

IF ID = lag(ID) and lag(Sepsis_grad) = 5 Sepsis_grad = 5.

      EXECUTE.

SELECT IF CounterIVA = 1.

   EXECUTE.

DESCRIPTIVES ID.
```

```
NUMERIC IMV(f2).
   VALUE LABELS IMV 1 'IMV' 0 'No IMV'.
NUMERIC CRRT(f2).
   VALUE LABELS CRRT 1 'CRRT' 0 'No CRRT'.
   IF IMV_tid > 0 IMV = 1.
   IF sysmis(IMV) IMV = 0.
   IF CRRT_tid > 0 CRRT = 1.
   IF sysmis(CRRT) CRRT = 0.
      EXECUTE.

NUMERIC ICU_LoS(f6).
      VARIABLE LABELS ICU_LoS 'Total sammanhängande vårdtid på IVA, days.'.
   COMPUTE ICU_LoS = datediff(UtskrTid,InskrTid,'hours').
   COMPUTE ICU_LoS = ICU_LoS/24.
   EXECUTE.
NUMERIC Dead_ICU_n(f1).
   IF Avliden_IVA = 'Ja' DeadICU_n = 1.
   IF sysmis(DeadICU_n) DeadICU_n = 0.
IF DeadICU_n = 1 DeathDate_ICU = UtskrivDatum.
   FORMATS DeathDate_ICU(date11).
NUMERIC Dead_n(f2).
   IF Avliden = 'Ja' Dead_n = 1.
   IF sysmis(Dead_n) Dead_n = 0.
IF Dead_n = 1 DeathDate = AvregDatum.
   FORMATS DeathDate(date11).

   ADD FILES FILE*
   /KEEP ID InskrTid UtskrTid InskrivDatum UtskrivDatum DeadICU_n DeathDate_ICU Dead_n DeathDate
SjukhusTyp Alder Kon Opererad SAPS3Score SAPS3_u_comorb
```

```
   SAPS3ScoreBoxIII Operationstyp APACHE_Score APACHE_AkutFysPoang Status IMV_tid CRRT_tid Sepsis_grad
Sepsis IMV CRRT ICU_LoS.
     EXECUTE.

SAVE OUTFILE 'Sepsis_temp.sav'.
*lägg till CCI och diagnoser.

*Hämta ut_par_sv_8996_2017.sav.

Get file 'Y:\Original_SPSS\ut_par_sv_8996_2017.sav'.
DATASET NAME Soc WINDOW=FRONT.
DATASET ACTIVATE Soc.

RENAME VARIABLES (lopnr LKF Kon = ID L Kon).
   FORMATS ID(f10).
     EXECUTE.
DELETE VARIABLES KON FODDAT ALDER to UTDATUMA UTDATUMA PVARD HDIA DIA_ANT SENUTV drg
SJUKHUSNAMN.
   EXECUTE.
STRING LKF(a10).
COMPUTE LKF = concat(rtrim(LK),' ',rtrim(L)).
   EXECUTE.
   DELETE VARIABLES LK L.
     EXECUTE.
SORT CASES ID(a) INDATUM(a) UTDATUM(a).
   NUMERIC CounterVTF(f3).
   IF $casenum = 1 or ID ne lag(ID) CounterVTF = 1.
   IF ID = lag(ID) CounterVTF = lag(CounterVTF) + 1.
     EXECUTE.
```

```
DATASET ACTIVATE Soc.
MATCH FILES /FILE=*
  /TABLE='Sepsis'
  /BY ID.
EXECUTE.

DATASET CLOSE Sepsis.
    DATASET ACTIVATE Soc.
    DATASET NAME Sepsis.
    DATASET ACTIVATE Sepsis.

SELECT IF InskrTid >0.
    EXECUTE.

DELETE VARIABLES CounterVTF.
    EXECUTE.
SORT CASES ID(a) INDATUM(a) UTDATUM(a).
    NUMERIC CounterVTF(f3).
    IF $casenum = 1 or ID ne lag(ID) CounterVTF = 1.
    IF ID = lag(ID) CounterVTF = lag(CounterVTF) + 1.
        EXECUTE.

SAVE outfile 'Sepsis_soc.sav'.
   * GET FILE 'Sepsis_soc.sav'.
   * DATASET NAME Sepsis WINDOW=FRONT.
   * DATASET ACTIVATE Sepsis.
*Skapa comorbiditetsvariabeln.
DATASET NAME Sepsis_CCI.
```

```
SELECT IF INDATUM < InskrivDatum.
   EXECUTE.

ADD FILES FILE*/KEEP ID CounterVTF Diagnos.
   EXECUTE.

SORT CASES BY ID CounterVTF.
CASESTOVARS
  /ID=ID
  /INDEX=CounterVTF
  /GROUPBY=VARIABLE.

*Slå ihop diagnosvariablerna.
STRING v(A3245).
VECTOR v=DIAGNOS.1 TO DIAGNOS.257.
LOOP cnt=1 TO 257.
- COMPUTE v=CONCAT(RTRIM(v)," ",v(cnt)," ") .
END LOOP.
- COMPUTE v=CONCAT(RTRIM(v),v(257)) .
   EXECUTE.

VARIABLE WIDTH  v (15).

RENAME VARIABLES v = Comorbidity_IVA.
VARIABLE LABELS Comorbidity_IVA 'Comorbiditet till och med vtf före in IVA'.

*Räkna tecken i Comorbidity_IVA så att Kolla att diagnoserna får plats (3237).
compute count_Comorbidity_IVA=char.length(Comorbidity_IVA).
   EXECUTE.
```

```
DESCRIPTIVES VARIABLES=count_Comorbidity_IVA
  /STATISTICS=MEAN MIN MAX.


*Ta bort överblivna variabler.
    DELETE VARIABLES Diagnos.1 to Diagnos.257 count_Comorbidity_IVA cnt.
     EXECUTE.


RENAME VARIABLES (Comorbidity_IVA = ICD).


*Create CCI according to Quan.
NUMERIC CCI_1(f2).
NUMERIC CCI_2(f2).
NUMERIC CCI_3(f2).
NUMERIC CCI_4(f2).
NUMERIC CCI_5(f2).
NUMERIC CCI_6(f2).
NUMERIC CCI_7(f2).
NUMERIC CCI_8(f2).
NUMERIC CCI_9(f2).
NUMERIC CCI_10(f2).
NUMERIC CCI_11(f2).
NUMERIC CCI_12(f2).
NUMERIC CCI_13(f2).
NUMERIC CCI_14(f2).
NUMERIC CCI_15(f2).
NUMERIC CCI_16(f2).
NUMERIC CCI_17(f2).
Numeric CCI(f8).
    VARIABLE LABELS CCI 'CCI according to Quan'.
```

```
*The code will give CCI.

*CCI 1.

COMPUTE CCI_1 =

   (CHAR.INDEX(UPCASE(ICD),'I21') > 0) OR

   (CHAR.INDEX(UPCASE(ICD),'I22') > 0) OR

   (CHAR.INDEX(UPCASE(ICD),'I252') > 0).


      EXECUTE.

   VARIABLE LABELS CCI_1 'Myocardial Infarction'.

   VALUE LABELS CCI_1 0 'no myocardial infarction' 1 'myocardial infarction'.


*CCI 2.

COMPUTE CCI_2 =

    (CHAR.INDEX(UPCASE(ICD),'I43') > 0) OR

    (CHAR.INDEX(UPCASE(ICD),'I50') > 0) OR

    (CHAR.INDEX(UPCASE(ICD),'I099') > 0) OR

    (CHAR.INDEX(UPCASE(ICD),'I110') > 0) OR

    (CHAR.INDEX(UPCASE(ICD),'I130') > 0) OR

    (CHAR.INDEX(UPCASE(ICD),'I132') > 0) OR

    (CHAR.INDEX(UPCASE(ICD),'I255') > 0) OR

    (CHAR.INDEX(UPCASE(ICD),'I420') > 0) OR

    (CHAR.INDEX(UPCASE(ICD),'I425') > 0) OR

    (CHAR.INDEX(UPCASE(ICD),'I426') > 0) OR

    (CHAR.INDEX(UPCASE(ICD),'I427') > 0) OR

    (CHAR.INDEX(UPCASE(ICD),'I428') > 0) OR

    (CHAR.INDEX(UPCASE(ICD),'I429') > 0) OR

    (CHAR.INDEX(UPCASE(ICD),'P290') > 0).
```

```
    EXECUTE.
  VARIABLE LABELS CCI_2 'Congestive Heart Failure'.
  VALUE LABELS CCI_2 0 'no Congestive Heart Failure' 1 'Congestive Heart Failure'.

*CCI 3.
COMPUTE CCI_3 =
  (CHAR.INDEX(UPCASE(ICD),'I70') > 0) OR
  (CHAR.INDEX(UPCASE(ICD),'I71') > 0) OR
  (CHAR.INDEX(UPCASE(ICD),'I731') > 0) OR
  (CHAR.INDEX(UPCASE(ICD),'I738') > 0) OR
  (CHAR.INDEX(UPCASE(ICD),'I739') > 0) OR
  (CHAR.INDEX(UPCASE(ICD),'I771') > 0) OR
  (CHAR.INDEX(UPCASE(ICD),'I790') > 0) OR
  (CHAR.INDEX(UPCASE(ICD),'I792') > 0) OR
  (CHAR.INDEX(UPCASE(ICD),'K551') > 0) OR
  (CHAR.INDEX(UPCASE(ICD),'K558') > 0) OR
  (CHAR.INDEX(UPCASE(ICD),'K559') > 0) OR
  (CHAR.INDEX(UPCASE(ICD),'Z958') > 0) OR
  (CHAR.INDEX(UPCASE(ICD),'Z959') > 0).

    EXECUTE.
  VARIABLE LABELS CCI_3 'Peripheral Vascular Disease'.
  VALUE LABELS CCI_3 0 'no Peripheral Vascular Disease' 1 'Peripheral Vascular Disease'.

*CCI 4.
COMPUTE CCI_4 =
  (CHAR.INDEX(UPCASE(ICD),'G45') > 0) OR
  (CHAR.INDEX(UPCASE(ICD),'G46') > 0) OR
  (CHAR.INDEX(UPCASE(ICD),'H340') > 0) OR
```

```
   (CHAR.INDEX(UPCASE(ICD),'I6') > 0).

      EXECUTE.
   VARIABLE LABELS CCI_4 'Cerebrovascular Disease'.
   VALUE LABELS CCI_4 0 'no Cerebrovascular Disease' 1 'Cerebrovascular Disease'.

*CCI 5.
COMPUTE CCI_5 =
   (CHAR.INDEX(UPCASE(ICD),'F00') > 0) OR
   (CHAR.INDEX(UPCASE(ICD),'F01') > 0) OR
   (CHAR.INDEX(UPCASE(ICD),'F02') > 0) OR
   (CHAR.INDEX(UPCASE(ICD),'F03') > 0) OR
   (CHAR.INDEX(UPCASE(ICD),'F051') > 0) OR
   (CHAR.INDEX(UPCASE(ICD),'G30') > 0) OR
   (CHAR.INDEX(UPCASE(ICD),'G311') > 0).

      EXECUTE.
VARIABLE LABELS CCI_5 'Dementia'.
VALUE LABELS CCI_5 0 'no Dementia' 1 'Dementia'.

*CCI 6.
COMPUTE CCI_6 =
   (CHAR.INDEX(UPCASE(ICD),'I278') > 0) OR
   (CHAR.INDEX(UPCASE(ICD),'I279') > 0) OR
   (CHAR.INDEX(UPCASE(ICD),'IJ40') > 0) OR
   (CHAR.INDEX(UPCASE(ICD),'J41') > 0) OR
   (CHAR.INDEX(UPCASE(ICD),'J42') > 0) OR
   (CHAR.INDEX(UPCASE(ICD),'J43') > 0) OR
```

```
    (CHAR.INDEX(UPCASE(ICD),'J44') > 0) OR
    (CHAR.INDEX(UPCASE(ICD),'J45') > 0) OR
    (CHAR.INDEX(UPCASE(ICD),'J46') > 0) OR
    (CHAR.INDEX(UPCASE(ICD),'J47') > 0) OR
    (CHAR.INDEX(UPCASE(ICD),'J60') > 0) OR
    (CHAR.INDEX(UPCASE(ICD),'J61') > 0) OR
    (CHAR.INDEX(UPCASE(ICD),'J62') > 0) OR
    (CHAR.INDEX(UPCASE(ICD),'J63') > 0) OR
    (CHAR.INDEX(UPCASE(ICD),'J64') > 0) OR
    (CHAR.INDEX(UPCASE(ICD),'J65') > 0) OR
    (CHAR.INDEX(UPCASE(ICD),'J66') > 0) OR
    (CHAR.INDEX(UPCASE(ICD),'J67') > 0) OR
    (CHAR.INDEX(UPCASE(ICD),'J684') > 0) OR
    (CHAR.INDEX(UPCASE(ICD),'J701') > 0) OR
    (CHAR.INDEX(UPCASE(ICD),'J703') > 0).

      EXECUTE.
VARIABLE LABELS CCI_6 'Chronic Pulmonary Disease'.
VALUE LABELS CCI_6 0 'no Chronic Pulmonary Disease' 1 'Chronic Pulmonary Disease'.

*CCI 7.
COMPUTE CCI_7 =
    (CHAR.INDEX(UPCASE(ICD),'M05') > 0) OR
    (CHAR.INDEX(UPCASE(ICD),'M06') > 0) OR
    (CHAR.INDEX(UPCASE(ICD),'M315') > 0) OR
    (CHAR.INDEX(UPCASE(ICD),'M32') > 0) OR
    (CHAR.INDEX(UPCASE(ICD),'M33') > 0) OR
    (CHAR.INDEX(UPCASE(ICD),'M34') > 0) OR
    (CHAR.INDEX(UPCASE(ICD),'M351') > 0) OR
```

```
   (CHAR.INDEX(UPCASE(ICD),'M353') > 0) OR
   (CHAR.INDEX(UPCASE(ICD),'M360') > 0) OR
   (CHAR.INDEX(UPCASE(ICD),'J47') > 0).

      EXECUTE.
VARIABLE LABELS CCI_7 'Connective Tissue Disease-Rheumatic Disease'.
VALUE LABELS CCI_7 0 'no Connective Tissue Disease-Rheumatic Disease' 1 'Connective Tissue Disease-Rheumatic Disease'.

*CCI 8.
COMPUTE CCI_8 =
   (CHAR.INDEX(UPCASE(ICD),'K25') > 0) OR
   (CHAR.INDEX(UPCASE(ICD),'K26') > 0) OR
   (CHAR.INDEX(UPCASE(ICD),'K27') > 0) OR
   (CHAR.INDEX(UPCASE(ICD),'K28') > 0).

      EXECUTE.
VARIABLE LABELS CCI_8 'Peptic Ulcer Disease'.
VALUE LABELS CCI_8 0 'no Peptic Ulcer Disease' 1 'Peptic Ulcer Disease'.

*CCI 9.
COMPUTE CCI_9 = (CHAR.INDEX(UPCASE(ICD),'B18') > 0) OR
   (CHAR.INDEX(UPCASE(ICD),'K700') > 0) OR
   (CHAR.INDEX(UPCASE(ICD),'K701') > 0) OR
   (CHAR.INDEX(UPCASE(ICD),'K702') > 0) OR
   (CHAR.INDEX(UPCASE(ICD),'K703') > 0) OR
   (CHAR.INDEX(UPCASE(ICD),'K709') > 0) OR
   (CHAR.INDEX(UPCASE(ICD),'K713') > 0) OR
   (CHAR.INDEX(UPCASE(ICD),'K714') > 0) OR
   (CHAR.INDEX(UPCASE(ICD),'K715') > 0) OR
```

```
    (CHAR.INDEX(UPCASE(ICD),'K717') > 0) OR
    (CHAR.INDEX(UPCASE(ICD),'K73') > 0) OR
    (CHAR.INDEX(UPCASE(ICD),'K74') > 0) OR
    (CHAR.INDEX(UPCASE(ICD),'K760') > 0) OR
    (CHAR.INDEX(UPCASE(ICD),'K762') > 0) OR
    (CHAR.INDEX(UPCASE(ICD),'K763') > 0) OR
    (CHAR.INDEX(UPCASE(ICD),'K764') > 0) OR
    (CHAR.INDEX(UPCASE(ICD),'K768') > 0) OR
    (CHAR.INDEX(UPCASE(ICD),'K768') > 0) OR
    (CHAR.INDEX(UPCASE(ICD),'Z944') > 0).

      EXECUTE.
VARIABLE LABELS CCI_9 'Mild Liver Disease'.
VALUE LABELS CCI_9 0 'no Mild Liver Disease' 1 'Mild Liver Disease'.

*CCI 10.
COMPUTE CCI_10 =
    (CHAR.INDEX(UPCASE(ICD),'E100') > 0) OR
    (CHAR.INDEX(UPCASE(ICD),'E101') > 0) OR
    (CHAR.INDEX(UPCASE(ICD),'E106') > 0) OR
    (CHAR.INDEX(UPCASE(ICD),'E108') > 0) OR
    (CHAR.INDEX(UPCASE(ICD),'E109') > 0) OR
    (CHAR.INDEX(UPCASE(ICD),'E110') > 0) OR
    (CHAR.INDEX(UPCASE(ICD),'E111') > 0) OR
    (CHAR.INDEX(UPCASE(ICD),'E116') > 0) OR
    (CHAR.INDEX(UPCASE(ICD),'E118') > 0) OR
    (CHAR.INDEX(UPCASE(ICD),'E119') > 0) OR
    (CHAR.INDEX(UPCASE(ICD),'E120') > 0) OR
    (CHAR.INDEX(UPCASE(ICD),'E121') > 0) OR
```

```
    (CHAR.INDEX(UPCASE(ICD),'E126') > 0) OR
    (CHAR.INDEX(UPCASE(ICD),'E128') > 0) OR
    (CHAR.INDEX(UPCASE(ICD),'E129') > 0) OR
    (CHAR.INDEX(UPCASE(ICD),'E130') > 0) OR
    (CHAR.INDEX(UPCASE(ICD),'E131') > 0) OR
    (CHAR.INDEX(UPCASE(ICD),'E136') > 0) OR
    (CHAR.INDEX(UPCASE(ICD),'E138') > 0) OR
    (CHAR.INDEX(UPCASE(ICD),'E139') > 0) OR
    (CHAR.INDEX(UPCASE(ICD),'E140') > 0) OR
    (CHAR.INDEX(UPCASE(ICD),'E141') > 0) OR
    (CHAR.INDEX(UPCASE(ICD),'E146') > 0) OR
    (CHAR.INDEX(UPCASE(ICD),'E148') > 0) OR
    (CHAR.INDEX(UPCASE(ICD),'E149') > 0).

      EXECUTE.
VARIABLE LABELS CCI_10 'Diabetes without complications'.
VALUE LABELS CCI_10 0 'no Diabetes without complications' 1 'Diabetes without complications'.

*CCI 11.
COMPUTE CCI_11 =
    (CHAR.INDEX(UPCASE(ICD),'E102') > 0) OR
    (CHAR.INDEX(UPCASE(ICD),'E103') > 0) OR
    (CHAR.INDEX(UPCASE(ICD),'E104') > 0) OR
    (CHAR.INDEX(UPCASE(ICD),'E105') > 0) OR
    (CHAR.INDEX(UPCASE(ICD),'E107') > 0) OR
    (CHAR.INDEX(UPCASE(ICD),'E112') > 0) OR
    (CHAR.INDEX(UPCASE(ICD),'E113') > 0) OR
    (CHAR.INDEX(UPCASE(ICD),'E114') > 0) OR
    (CHAR.INDEX(UPCASE(ICD),'E115') > 0) OR
```

```
   (CHAR.INDEX(UPCASE(ICD),'E117') > 0) OR
   (CHAR.INDEX(UPCASE(ICD),'E122') > 0) OR
   (CHAR.INDEX(UPCASE(ICD),'E123') > 0) OR
   (CHAR.INDEX(UPCASE(ICD),'E124') > 0) OR
   (CHAR.INDEX(UPCASE(ICD),'E125') > 0) OR
   (CHAR.INDEX(UPCASE(ICD),'E127') > 0) OR
   (CHAR.INDEX(UPCASE(ICD),'E132') > 0) OR
   (CHAR.INDEX(UPCASE(ICD),'E133') > 0) OR
   (CHAR.INDEX(UPCASE(ICD),'E134') > 0) OR
   (CHAR.INDEX(UPCASE(ICD),'E135') > 0) OR
   (CHAR.INDEX(UPCASE(ICD),'E137') > 0) OR
   (CHAR.INDEX(UPCASE(ICD),'E142') > 0) OR
   (CHAR.INDEX(UPCASE(ICD),'E143') > 0) OR
   (CHAR.INDEX(UPCASE(ICD),'E144') > 0) OR
   (CHAR.INDEX(UPCASE(ICD),'E145') > 0) OR
   (CHAR.INDEX(UPCASE(ICD),'E147') > 0).

     EXECUTE.
VARIABLE LABELS CCI_11 'Diabetes with chronic complication'.
VALUE LABELS CCI_11 0 'no Diabetes with chronic complication' 1 'Diabetes with chronic complication'.

*CCI 12.
COMPUTE CCI_12 =
   (CHAR.INDEX(UPCASE(ICD),'G041') > 0) OR
   (CHAR.INDEX(UPCASE(ICD),'G114') > 0) OR
   (CHAR.INDEX(UPCASE(ICD),'G801') > 0) OR
   (CHAR.INDEX(UPCASE(ICD),'G802') > 0) OR
   (CHAR.INDEX(UPCASE(ICD),'G81') > 0) OR
   (CHAR.INDEX(UPCASE(ICD),'G82') > 0) OR
```

```
    (CHAR.INDEX(UPCASE(ICD),'G830') > 0) OR
    (CHAR.INDEX(UPCASE(ICD),'G831') > 0) OR
    (CHAR.INDEX(UPCASE(ICD),'G832') > 0) OR
    (CHAR.INDEX(UPCASE(ICD),'G833') > 0) OR
    (CHAR.INDEX(UPCASE(ICD),'G834') > 0) OR
    (CHAR.INDEX(UPCASE(ICD),'G839') > 0).

      EXECUTE.
VARIABLE LABELS CCI_12 'Hemiplegia or paraplegia'.
VALUE LABELS CCI_12 0 'no Hemiplegia or paraplegia' 1 'Hemiplegia or paraplegia'.

*CCI 13.
COMPUTE CCI_13 =
    (CHAR.INDEX(UPCASE(ICD),'I120') > 0) OR
    (CHAR.INDEX(UPCASE(ICD),'I131') > 0) OR
    (CHAR.INDEX(UPCASE(ICD),'N032') > 0) OR
    (CHAR.INDEX(UPCASE(ICD),'N033') > 0) OR
    (CHAR.INDEX(UPCASE(ICD),'N034') > 0) OR
    (CHAR.INDEX(UPCASE(ICD),'N035') > 0) OR
    (CHAR.INDEX(UPCASE(ICD),'N036') > 0) OR
    (CHAR.INDEX(UPCASE(ICD),'N037') > 0) OR
    (CHAR.INDEX(UPCASE(ICD),'N052') > 0) OR
    (CHAR.INDEX(UPCASE(ICD),'N053') > 0) OR
    (CHAR.INDEX(UPCASE(ICD),'N054') > 0) OR
    (CHAR.INDEX(UPCASE(ICD),'N055') > 0) OR
    (CHAR.INDEX(UPCASE(ICD),'N056') > 0) OR
    (CHAR.INDEX(UPCASE(ICD),'N057') > 0) OR
    (CHAR.INDEX(UPCASE(ICD),'N18') > 0) OR
    (CHAR.INDEX(UPCASE(ICD),'N19') > 0) OR
```

```
(CHAR.INDEX(UPCASE(ICD),'N250') > 0) OR
(CHAR.INDEX(UPCASE(ICD),'Z490') > 0) OR
(CHAR.INDEX(UPCASE(ICD),'Z491') > 0) OR
(CHAR.INDEX(UPCASE(ICD),'Z492') > 0) OR
(CHAR.INDEX(UPCASE(ICD),'Z940') > 0) OR
(CHAR.INDEX(UPCASE(ICD),'Z992') > 0).

EXECUTE.
VARIABLE LABELS CCI_13 'Renal disease'.
VALUE LABELS CCI_13 0 'no Renal disease' 1 'Renal disease'.

*CCI 14.
COMPUTE CCI_14 =
(CHAR.INDEX(UPCASE(ICD),'C0') > 0) OR
(CHAR.INDEX(UPCASE(ICD),'C30') > 0) OR
(CHAR.INDEX(UPCASE(ICD),'C31') > 0) OR
(CHAR.INDEX(UPCASE(ICD),'C32') > 0) OR
(CHAR.INDEX(UPCASE(ICD),'C33') > 0) OR
(CHAR.INDEX(UPCASE(ICD),'C34') > 0) OR
(CHAR.INDEX(UPCASE(ICD),'C37') > 0) OR
(CHAR.INDEX(UPCASE(ICD),'C38') > 0) OR
(CHAR.INDEX(UPCASE(ICD),'C39') > 0) OR
(CHAR.INDEX(UPCASE(ICD),'C40') > 0) OR
(CHAR.INDEX(UPCASE(ICD),'C41') > 0) OR
(CHAR.INDEX(UPCASE(ICD),'C43') > 0) OR
(CHAR.INDEX(UPCASE(ICD),'C45') > 0) OR
(CHAR.INDEX(UPCASE(ICD),'C46') > 0) OR
(CHAR.INDEX(UPCASE(ICD),'C47') > 0) OR
(CHAR.INDEX(UPCASE(ICD),'C48') > 0) OR
```

```
(CHAR.INDEX(UPCASE(ICD),'C49') > 0) OR
(CHAR.INDEX(UPCASE(ICD),'C50') > 0) OR
(CHAR.INDEX(UPCASE(ICD),'C51') > 0) OR
(CHAR.INDEX(UPCASE(ICD),'C52') > 0) OR
(CHAR.INDEX(UPCASE(ICD),'C53') > 0) OR
(CHAR.INDEX(UPCASE(ICD),'C54') > 0) OR
(CHAR.INDEX(UPCASE(ICD),'C55') > 0) OR
(CHAR.INDEX(UPCASE(ICD),'C56') > 0) OR
(CHAR.INDEX(UPCASE(ICD),'C57') > 0) OR
(CHAR.INDEX(UPCASE(ICD),'C58') > 0) OR
(CHAR.INDEX(UPCASE(ICD),'C6') > 0) OR
(CHAR.INDEX(UPCASE(ICD),'C70') > 0) OR
(CHAR.INDEX(UPCASE(ICD),'C71') > 0) OR
(CHAR.INDEX(UPCASE(ICD),'C72') > 0) OR
(CHAR.INDEX(UPCASE(ICD),'C73') > 0) OR
(CHAR.INDEX(UPCASE(ICD),'C74') > 0) OR
(CHAR.INDEX(UPCASE(ICD),'C75') > 0) OR
(CHAR.INDEX(UPCASE(ICD),'C76') > 0) OR
(CHAR.INDEX(UPCASE(ICD),'C81') > 0) OR
(CHAR.INDEX(UPCASE(ICD),'C82') > 0) OR
(CHAR.INDEX(UPCASE(ICD),'C83') > 0) OR
(CHAR.INDEX(UPCASE(ICD),'C84') > 0) OR
(CHAR.INDEX(UPCASE(ICD),'C85') > 0) OR
(CHAR.INDEX(UPCASE(ICD),'C88') > 0) OR
(CHAR.INDEX(UPCASE(ICD),'C90') > 0) OR
(CHAR.INDEX(UPCASE(ICD),'C91') > 0) OR
(CHAR.INDEX(UPCASE(ICD),'C92') > 0) OR
(CHAR.INDEX(UPCASE(ICD),'C93') > 0) OR
(CHAR.INDEX(UPCASE(ICD),'C94') > 0) OR
```

```
  (CHAR.INDEX(UPCASE(ICD),'C95') > 0) OR
  (CHAR.INDEX(UPCASE(ICD),'C96') > 0) OR
  (CHAR.INDEX(UPCASE(ICD),'C97') > 0).

    EXECUTE.
   VARIABLE LABELS CCI_14 'Any malignancy, including lymphoma and leukemia, except malignant neoplasm of skin'.
   VALUE LABELS CCI_14 0 'no Any malignancy, including lymphoma and leukemia, except malignant neoplasm of skin' 1 'Any malignancy, including lymphoma and leukemia, except malignant neoplasm of skin'.

*CCI 15.
COMPUTE CCI_15 =
   (CHAR.INDEX(UPCASE(ICD),'I850') > 0) OR
   (CHAR.INDEX(UPCASE(ICD),'I859') > 0) OR
   (CHAR.INDEX(UPCASE(ICD),'I864') > 0) OR
   (CHAR.INDEX(UPCASE(ICD),'I982') > 0) OR
   (CHAR.INDEX(UPCASE(ICD),'K704') > 0) OR
   (CHAR.INDEX(UPCASE(ICD),'K711') > 0) OR
   (CHAR.INDEX(UPCASE(ICD),'K721') > 0) OR
   (CHAR.INDEX(UPCASE(ICD),'K729') > 0) OR
   (CHAR.INDEX(UPCASE(ICD),'K765') > 0) OR
   (CHAR.INDEX(UPCASE(ICD),'K766') > 0) OR
   (CHAR.INDEX(UPCASE(ICD),'K767') > 0).

    EXECUTE.
   VARIABLE LABELS CCI_15 'Moderate or severe liver disease'.
   VALUE LABELS CCI_15 0 'no Moderate or severe liver disease' 1 'Moderate or severe liver disease'.

*CCI 16.
COMPUTE CCI_16 =
```

```
   (CHAR.INDEX(UPCASE(ICD),'C77') > 0) OR
   (CHAR.INDEX(UPCASE(ICD),'C78') > 0) OR
   (CHAR.INDEX(UPCASE(ICD),'C79') > 0) OR
   (CHAR.INDEX(UPCASE(ICD),'C80')  > 0).

      EXECUTE.
   VARIABLE LABELS CCI_16 'Metastatic solid tumor'.
   VALUE LABELS CCI_16 0 'no Metastatic solid tumor' 1 'Metastatic solid tumor'.

*CCI 17.
COMPUTE CCI_17 =
   (CHAR.INDEX(UPCASE(ICD),'B20') > 0) OR
   (CHAR.INDEX(UPCASE(ICD),'B21') > 0) OR
   (CHAR.INDEX(UPCASE(ICD),'B22') > 0) OR
   (CHAR.INDEX(UPCASE(ICD),'B24') > 0).

      EXECUTE.
VARIABLE LABELS CCI_17 'AIDS/HIV'.
VALUE LABELS CCI_17 0 'no AIDS/HIV' 1 'AIDS/HIV'.

RECODE CCI_1 to CCI_17 (0=0) (Else = 1).
   EXECUTE.
*Add up CCI.
COMPUTE CCI = 0.
If CCI_1 > 0 CCI = CCI + 0.
If CCI_2 > 0 CCI = CCI + 2.
If CCI_3 > 0 CCI = CCI + 0.
If CCI_4 > 0 CCI = CCI + 0.
If CCI_5 > 0 CCI = CCI + 2.
```

```
If CCI_6 > 0 CCI = CCI + 1.

If CCI_7 > 0 CCI = CCI + 1.

If CCI_8 > 0 CCI = CCI + 0.

If CCI_9 > 0 CCI = CCI + 2.

If CCI_10 > 0 CCI = CCI + 0.

If CCI_11 > 0 CCI = CCI + 1.

If CCI_12 > 0 CCI = CCI + 2.

If CCI_13 > 0 CCI = CCI + 1.

If CCI_14 > 0 CCI = CCI + 2.

If CCI_15 > 0 CCI = CCI + 4.

If CCI_16 > 0 CCI = CCI + 6.

If CCI_17 > 0 CCI = CCI + 4.


   EXECUTE.


*Subtract mild liver and tumor disease from those with severe disease.

IF CCI_9 > 0 and CCI_15 > 0 CCI = CCI - 2.

IF CCI_14 > 0 and CCI_16 > 0 CCI = CCI - 2.

   EXECUTE.


   FREQUENCIES CCI_1 to CCI.


SAVE OUTFILE 'Sepsis_CCI.sav'.

ADD FILES FILE*/KEEP ID CCI.

   EXECUTE.


GET FILE 'Sepsis_soc.sav'.

DATASET NAME Sepsis WINDOW=FRONT.

DATASET ACTIVATE Sepsis.
```

```
*Hitta komorbiditeterna.

NUMERIC Tid_Diagn_Inkl(f6).

      VARIABLE LABELS Tid_Diagn_Inkl 'Tid från diagnos till inklusion'.

COMPUTE Tid_Diagn_Inkl = datediff(InskrivDatum,UTDATUM,'days').

   EXECUTE.


NUMERIC Tid_Atg_Inkl(f6).

      VARIABLE LABELS Tid_Atg_Inkl 'Tid från åtgärd till inklusion'.

COMPUTE Tid_Atg_Inkl = datediff(InskrivDatum,UTDATUM,'days').

   EXECUTE.


*Ta bort åtgärder och diagnoser som inte varit före IVA med Sepsis.

SELECT IF Tid_Diagn_Inkl > 0.

   EXECUTE.


*Hitta Cytoadministreringar.

NUMERIC CytoAdm(f2).

NUMERIC CytoAdm2(f2).

   VARIABLE LABELS CytoAdm 'Cytoadministreringar -åtgärd- sista sex månaderna'.

   VARIABLE LABELS CytoAdm2 'Cytoadministreringar -åtgärd- sista 12 månaderna'.

   VALUE LABELS CytoAdm 0 'No' 1 'Yes'.

   COMPUTE AA = (CHAR.INDEX(UPCASE(Op),'DT107') > 0).

   COMPUTE BB = (CHAR.INDEX(UPCASE(Op),'DT108') > 0).

   COMPUTE CC = (CHAR.INDEX(UPCASE(Op),'DT112') > 0).

   COMPUTE DD = (CHAR.INDEX(UPCASE(Op),'DT116') > 0).
```

```
   COMPUTE EE = (CHAR.INDEX(UPCASE(Op),'DT135') > 0).
   IF AA > 0 or  BB > 0 or CC > 0 or DD > 0  or EE > 0 CytoAdm = 1.
      If CytoAdm = 1 CytoAdm2 = 1.
      If Tid_Atg_Inkl > 365 CytoAdm2 = 0.
      IF sysmis(CytoAdm2) CytoAdm2= 0.
   If Tid_Atg_Inkl > 182 CytoAdm = 0.
   IF sysmis(CytoAdm) CytoAdm= 0.
      EXECUTE.
   DELETE VARIABLES AA BB CC DD EE.
      EXECUTE.

*Hitta Strålbehandlingar.
NUMERIC Radiak(f2).
NUMERIC Radiak2(f2).
   VARIABLE LABELS Radiak 'Strålbehandlingar -åtgärd- sista sex månaderna'.
   VARIABLE LABELS Radiak2 'Strålbehandlingar -åtgärd- sista 12 månaderna'.
   VALUE LABELS Radiak 0 'No' 1 'Yes'.
   COMPUTE AA = (CHAR.INDEX(UPCASE(Op),'DV069') > 0).
   COMPUTE BB = (CHAR.INDEX(UPCASE(Op),'DV070') > 0).
   COMPUTE CC = (CHAR.INDEX(UPCASE(Op),'DV071') > 0).
   COMPUTE DD = (CHAR.INDEX(UPCASE(Op),'DV072') > 0).
   IF AA > 0 or  BB > 0 or CC > 0 or DD > 0 Radiak = 1.
   IF Radiak = 1 Radiak2 = 1.
      If Tid_Atg_Inkl > 365 Radiak2 = 0.
      IF sysmis(Radiak2) Radiak2= 0.
   If Tid_Atg_Inkl > 182 Radiak = 0.
   IF sysmis(Radiak) Radiak= 0.
      EXECUTE.
   DELETE VARIABLES AA BB CC DD.
```

EXECUTE.

*Hitta Hemodialys.

NUMERIC HD_Cron(f2).

VARIABLE LABELS HD_Cron 'Intermittent hemodialys -åtgärd- sista sex månaderna'.

VALUE LABELS HD_Cron 0 'No' 1 'Yes'.

COMPUTE AA = (CHAR.INDEX(UPCASE(Op),'DR016') > 0).

IF AA > 0 HD_Cron = 1.

If Tid_Atg_Inkl > 182 HD_Cron = 0.

IF sysmis(HD_Cron) HD_Cron= 0.

EXECUTE.

DELETE VARIABLES AA.

EXECUTE.

*Hitta Diagnos///. Obs 5 årr!!! fixa datasetet!!!!!.

*OBS för diabetes II, övervikt och HT söks efter lm 5 år tillbaka!!!! Samma för åtärder för njursvikt - HD PD´och övervikt.

*Hitta Ischemisk hjärtsjukdom.

NUMERIC IHD(f2).

VARIABLE LABELS IHD 'Ischemisk hjärtsjukdom'.

VALUE LABELS IHD 0 'No' 1 'Yes'.

COMPUTE AA = (CHAR.INDEX(UPCASE(Diagnos),'I20') > 0).

COMPUTE BB = (CHAR.INDEX(UPCASE(Diagnos),'I21') > 0).

COMPUTE CC = (CHAR.INDEX(UPCASE(Diagnos),'I22') > 0).

COMPUTE DD = (CHAR.INDEX(UPCASE(Diagnos),'I23') > 0).

COMPUTE EE = (CHAR.INDEX(UPCASE(Diagnos),'I24') > 0).

COMPUTE FF = (CHAR.INDEX(UPCASE(Diagnos),'I25') > 0).

IF AA > 0 or BB > 0 or CC > 0 or DD > 0 or EE > 0 or FF > 0 IHD = 1.

```
   IF sysmis(IHD) IHD= 0.
      EXECUTE.
   DELETE VARIABLES AA BB CC DD EE FF.
      EXECUTE.

*Hitta annan hjärtsjukdom.
NUMERIC OtherHD(f2).
   VARIABLE LABELS OtherHD 'Annan hjärtsjukdom - icke IHD'.
   VALUE LABELS OtherHD 0 'No' 1 'Yes'.
   COMPUTE AA = (CHAR.INDEX(UPCASE(Diagnos),'I3') > 0).
   COMPUTE BB = (CHAR.INDEX(UPCASE(Diagnos),'I4') > 0).
   COMPUTE CC = (CHAR.INDEX(UPCASE(Diagnos),'I5') > 0).
   COMPUTE DD = (CHAR.INDEX(UPCASE(Diagnos),'I01') > 0).
   COMPUTE EE = (CHAR.INDEX(UPCASE(Diagnos),'I05') > 0).
   COMPUTE FF = (CHAR.INDEX(UPCASE(Diagnos),'I06') > 0).
   COMPUTE GG = (CHAR.INDEX(UPCASE(Diagnos),'I07') > 0).
   COMPUTE HH = (CHAR.INDEX(UPCASE(Diagnos),'I08') > 0).
   COMPUTE II = (CHAR.INDEX(UPCASE(Diagnos),'I09') > 0).
   COMPUTE JJ = (CHAR.INDEX(UPCASE(Diagnos),'I11') > 0).
   COMPUTE KK = (CHAR.INDEX(UPCASE(Diagnos),'I13') > 0).
   COMPUTE LL = (CHAR.INDEX(UPCASE(Diagnos),'I27') > 0).
   IF AA > 0 or  BB > 0 or CC > 0 or DD > 0 or EE > 0 or FF > 0 or GG > 0 or HH > 0 or II > 0 or JJ > 0 or KK > 0 or LL
> 0 OtherHD = 1.
   IF sysmis(OtherHD) OtherHD= 0.
      EXECUTE.
   DELETE VARIABLES AA BB CC DD EE FF GG HH II JJ KK LL.
      EXECUTE.

*Hitta hypertoni.
NUMERIC Hypertens(f2).
```

```
   VARIABLE LABELS Hypertens 'Hypertoni'.
   VALUE LABELS Hypertens 0 'No' 1 'Yes'.
   COMPUTE AA = (CHAR.INDEX(UPCASE(Diagnos),'I1') > 0).
   IF AA > 0 Hypertens = 1.
   IF sysmis(Hypertens) Hypertens = 0.
      EXECUTE.
   DELETE VARIABLES AA.
      EXECUTE.

*Hitta Diabetes typ I.
NUMERIC Diabetes1(f2).
   VARIABLE LABELS Diabetes1 'Diabetes typ 1'.
   VALUE LABELS Diabetes1 0 'No' 1 'Yes'.
   COMPUTE AA = (CHAR.INDEX(UPCASE(Diagnos),'E10') > 0).
   IF AA > 0 Diabetes1 = 1.
   IF sysmis(Diabetes1) Diabetes1 = 0.
      EXECUTE.
   DELETE VARIABLES AA.
      EXECUTE.

*Hitta Diabetes typ II.
NUMERIC Diabetes2(f2).
   VARIABLE LABELS Diabetes2 'Diabetes typ 2'.
   VALUE LABELS Diabetes2 0 'No' 1 'Yes'.
   COMPUTE AA = (CHAR.INDEX(UPCASE(Diagnos),'E11') > 0).
      EXECUTE.
   IF AA > 0 Diabetes2 = 1.
   IF sysmis(Diabetes2) Diabetes2 = 0.
   EXECUTE.
```

```
   DELETE VARIABLES AA.
      EXECUTE.


*Hittta stroke.
NUMERIC Stroke(f2).
   VARIABLE LABELS Stroke 'Någon Cerebrovaskulär sjukdom'.
   VALUE LABELS Stroke 0 'No' 1 'Yes'.
   COMPUTE AA = (CHAR.INDEX(UPCASE(Diagnos),'I6') > 0).
   IF AA > 0 Stroke = 1.
   IF sysmis(Stroke) Stroke = 0.
      EXECUTE.
   DELETE VARIABLES AA.
      EXECUTE.


*Hitta kronisk njursvikt.
NUMERIC Njursvikt(f2).
   VARIABLE LABELS Njursvikt 'Njursvikt'.
   VALUE LABELS Njursvikt 0 'No' 1 'Yes'.
   COMPUTE N181 = (CHAR.INDEX(UPCASE(Diagnos),'N181') > 0).
   COMPUTE N182 = (CHAR.INDEX(UPCASE(Diagnos),'N182') > 0).
   COMPUTE N183 = (CHAR.INDEX(UPCASE(Diagnos),'N183') > 0).
   COMPUTE N184 = (CHAR.INDEX(UPCASE(Diagnos),'N184') > 0).
   COMPUTE N185 = (CHAR.INDEX(UPCASE(Diagnos),'N185') > 0).
   COMPUTE N189 = (CHAR.INDEX(UPCASE(Diagnos),'N189') > 0).
   *Hitta HD, PD senaste 5 åren.
      COMPUTE DR016 = (CHAR.INDEX(UPCASE(Op),'DR016') > 0).
      COMPUTE DR024 = (CHAR.INDEX(UPCASE(Op),'DR024') > 0).
      IF N181 > 0 or  N182 > 0 or  N183 > 0 or  N184 > 0 or  N185 > 0 or  N189 > 0 or  DR016 > 0 or DR024 > 0
Njursvikt = 1.
   IF sysmis(Njursvikt) Njursvikt = 0.
```

```
   RECODE N181 to DR024 (SYSMIS = 0).
      EXECUTE.


*Hitta KOL, inklusive emfysem och kronisk bronkit.
NUMERIC KOL(f2).
   VARIABLE LABELS KOL 'KOL, emfysem, kronisk bronkit'.
   VALUE LABELS KOL 0 'No' 1 'Yes'.
   COMPUTE AA = (CHAR.INDEX(UPCASE(Diagnos),'J41') > 0).
   COMPUTE BB = (CHAR.INDEX(UPCASE(Diagnos),'J42') > 0).
   COMPUTE CC = (CHAR.INDEX(UPCASE(Diagnos),'J43') > 0).
   COMPUTE DD = (CHAR.INDEX(UPCASE(Diagnos),'J44') > 0).
   IF AA > 0 or  BB > 0 or CC > 0 or DD > 0 KOL = 1.
   IF sysmis(KOL) KOL= 0.
      EXECUTE.
   DELETE VARIABLES AA BB CC DD.
      EXECUTE.


*Hitta Astma.
NUMERIC Astma(f2).
   VARIABLE LABELS Astma 'Astma'.
   VALUE LABELS Astma 0 'No' 1 'Yes'.
   COMPUTE AA = (CHAR.INDEX(UPCASE(Diagnos),'J45') > 0).
   COMPUTE BB = (CHAR.INDEX(UPCASE(Diagnos),'J46') > 0).
   IF AA > 0 or  BB > 0 Astma = 1.
   IF sysmis(Astma) Astma = 0.
      EXECUTE.
   DELETE VARIABLES AA BB.
      EXECUTE.
```

```
*Hitta fetma inklusive överviktskirurgi.
NUMERIC Fetma(f2).
  VARIABLE LABELS Fetma 'Fetma'.
  VALUE LABELS Fetma 0 'No' 1 'Yes'.
  COMPUTE AA = (CHAR.INDEX(UPCASE(Diagnos),'E66') > 0).
  *Hitta Överviktskirurgi.
    COMPUTE BB = (CHAR.INDEX(UPCASE(Op),'JDF') > 0).
    COMPUTE CC = (CHAR.INDEX(UPCASE(Op),'JFD03') > 0).
    IF AA > 0 or  BB > 0 or CC > 0 Fetma = 1.
  IF sysmis(Fetma) Fetma = 0.
    EXECUTE.
  DELETE VARIABLES AA BB CC.
    EXECUTE.


*Hitta Immunsupprimerande sjukdom eller efter åtgärd.
NUMERIC Immunsupprimerad(f2).
  VARIABLE LABELS Immunsupprimerad 'Immunsupprimerad'.
  VALUE LABELS Immunsupprimerad 0 'No' 1 'Yes'.
  COMPUTE AA = (CHAR.INDEX(UPCASE(Diagnos),'D71') > 0).
  COMPUTE BB = (CHAR.INDEX(UPCASE(Diagnos),'D80') > 0).
  COMPUTE CC = (CHAR.INDEX(UPCASE(Diagnos),'D81') > 0).
  COMPUTE DD = (CHAR.INDEX(UPCASE(Diagnos),'D82') > 0).
  COMPUTE EE = (CHAR.INDEX(UPCASE(Diagnos),'D83') > 0).
  COMPUTE FF = (CHAR.INDEX(UPCASE(Diagnos),'D84') > 0).
  COMPUTE GG = (CHAR.INDEX(UPCASE(Diagnos),'D89') > 0).
  IF AA > 0 or  BB > 0 or CC > 0 or DD > 0 or EE > 0 or FF > 0 or GG > 0  Immunsupprimerad = 1.
  *Hitta immunsupprimerande åtgärder lm senaste 6 månaderna.
  IF CytoAdm = 1 or Radiak = 1 Immunsupprimerad = 1.
  IF sysmis(Immunsupprimerad)  Immunsupprimerad = 0.
```

```
    EXECUTE.
  DELETE VARIABLES AA BB CC DD EE FF GG.
    EXECUTE.

*Hitta aktiv solid tumör senaste året dvs diagnossatt Senaste 12 mån..
NUMERIC Cancer_dia(f2).
NUMERIC CancerDiagn(f2).
  VARIABLE LABELS Cancer_dia 'Cancer'.
  VALUE LABELS Cancer_dia 0 'No' 1 'Yes'.
  COMPUTE AA = (CHAR.INDEX(UPCASE(Diagnos),'C0') > 0).
  COMPUTE BB = (CHAR.INDEX(UPCASE(Diagnos),'C1') > 0).
  COMPUTE CC = (CHAR.INDEX(UPCASE(Diagnos),'C2') > 0).
  COMPUTE DD = (CHAR.INDEX(UPCASE(Diagnos),'C3') > 0).
  COMPUTE EE = (CHAR.INDEX(UPCASE(Diagnos),'C4') > 0).
  COMPUTE FF = (CHAR.INDEX(UPCASE(Diagnos),'C5') > 0).
  COMPUTE GG = (CHAR.INDEX(UPCASE(Diagnos),'C6') > 0).
  COMPUTE HH = (CHAR.INDEX(UPCASE(Diagnos),'C7') > 0).
  COMPUTE II = (CHAR.INDEX(UPCASE(Diagnos),'C80') > 0).
  COMPUTE JJ = (CHAR.INDEX(UPCASE(Diagnos),'C97') > 0).
   IF AA > 0 or  BB > 0 or CC > 0 or DD > 0 or EE > 0 or FF > 0 or GG > 0 or HH > 0 or II > 0 or JJ > 0 CancerDiagn =
1.
  IF Tid_Diagn_Inkl > 365 CancerDiagn = 0.
  If CancerDiagn = 1 Cancer_dia = 1.
  IF sysmis(Cancer_dia) Cancer_dia = 0.
    EXECUTE.
  DELETE VARIABLES AA to JJ CancerDiagn.
    EXECUTE.

*Hitta aktiv blodcancer senaste året dvs diagnossatt Senaste 12 mån..
NUMERIC BlodCancer(f2).
```

```
NUMERIC BlodCancerDiagn(f2).
   VARIABLE LABELS BlodCancer 'Hematologisk malignitet'.
   VALUE LABELS BlodCancer 0 'No' 1 'Yes'.
   COMPUTE AA = (CHAR.INDEX(UPCASE(Diagnos),'C81') > 0).
   COMPUTE BB = (CHAR.INDEX(UPCASE(Diagnos),'C82') > 0).
   COMPUTE CC = (CHAR.INDEX(UPCASE(Diagnos),'C83') > 0).
   COMPUTE DD = (CHAR.INDEX(UPCASE(Diagnos),'C84') > 0).
   COMPUTE EE = (CHAR.INDEX(UPCASE(Diagnos),'C85') > 0).
   COMPUTE FF = (CHAR.INDEX(UPCASE(Diagnos),'C86') > 0).
   COMPUTE GG = (CHAR.INDEX(UPCASE(Diagnos),'C88') > 0).
   COMPUTE HH = (CHAR.INDEX(UPCASE(Diagnos),'C90') > 0).
   COMPUTE II = (CHAR.INDEX(UPCASE(Diagnos),'C91') > 0).
   COMPUTE JJ = (CHAR.INDEX(UPCASE(Diagnos),'C92') > 0).
   COMPUTE KK = (CHAR.INDEX(UPCASE(Diagnos),'C93') > 0).
   COMPUTE LL = (CHAR.INDEX(UPCASE(Diagnos),'C94') > 0).
   COMPUTE MM = (CHAR.INDEX(UPCASE(Diagnos),'C95') > 0).
   COMPUTE NN = (CHAR.INDEX(UPCASE(Diagnos),'C96') > 0).
    IF AA > 0 or  BB > 0 or CC > 0 or DD > 0 or EE > 0 or FF > 0 or GG > 0 or HH > 0 or II > 0 or JJ > 0 or KK > 0 or LL
> 0 or MM > 0 or NN > 0 BlodCancerDiagn = 1.
   IF Tid_Diagn_Inkl > 365 BlodCancerDiagn = 0.
   If BlodCancerDiagn = 1 BlodCancer = 1.
   IF sysmis(BlodCancer) BlodCancer= 0.
      EXECUTE.
   DELETE VARIABLES AA to NN BlodCancerDiagn.
      EXECUTE.


*Hitta inflammatorisk sjukdom.
NUMERIC Inflam(f2).
   VARIABLE LABELS Inflam 'Systeminflammatorisk sjukdom'.
   VALUE LABELS Inflam 0 'No' 1 'Yes'.
```

```
   COMPUTE AA = (CHAR.INDEX(UPCASE(Diagnos),'M3') > 0).
   COMPUTE BB = (CHAR.INDEX(UPCASE(Diagnos),'M05') > 0).
   COMPUTE CC = (CHAR.INDEX(UPCASE(Diagnos),'M06') > 0).
   COMPUTE DD = (CHAR.INDEX(UPCASE(Diagnos),'M07') > 0).
   COMPUTE EE = (CHAR.INDEX(UPCASE(Diagnos),'M08') > 0).
   COMPUTE FF = (CHAR.INDEX(UPCASE(Diagnos),'M09') > 0).
   COMPUTE GG = (CHAR.INDEX(UPCASE(Diagnos),'M10') > 0).
   COMPUTE HH = (CHAR.INDEX(UPCASE(Diagnos),'M11') > 0).
   COMPUTE II = (CHAR.INDEX(UPCASE(Diagnos),'M12') > 0).
   COMPUTE JJ = (CHAR.INDEX(UPCASE(Diagnos),'M13') > 0).
   COMPUTE KK = (CHAR.INDEX(UPCASE(Diagnos),'M14') > 0).
   IF AA > 0 or  BB > 0 or CC > 0 or DD > 0 or EE > 0 or FF > 0 or GG > 0 or HH > 0 or II > 0 or JJ > 0 or KK > 0 Inflam
= 1.
   IF sysmis(Inflam) Inflam= 0.
     EXECUTE.
   DELETE VARIABLES AA BB CC DD EE FF GG HH II JJ KK.
     EXECUTE.
*Hitta transplanterade.
NUMERIC Transplanterad(f2).
   VARIABLE LABELS Transplanterad 'Transplanterad'.
   VALUE LABELS Transplanterad 0 'No' 1 'Yes'.
   COMPUTE AA = (CHAR.INDEX(UPCASE(Diagnos),'Z940') > 0).
   COMPUTE BB = (CHAR.INDEX(UPCASE(Diagnos),'Z941') > 0).
   COMPUTE CC = (CHAR.INDEX(UPCASE(Diagnos),'Z942') > 0).
   COMPUTE DD = (CHAR.INDEX(UPCASE(Diagnos),'Z943') > 0).
   COMPUTE EE = (CHAR.INDEX(UPCASE(Diagnos),'Z944') > 0).

   IF AA > 0 or  BB > 0 or CC > 0 or DD > 0 or EE > 0 Transplanterad = 1.
   IF sysmis(Transplanterad)  Transplanterad = 0.
     EXECUTE.
```

```
   DELETE VARIABLES AA BB CC DD EE.
      EXECUTE.

DELETE VARIABLES CounterVTF.
   EXECUTE.
SORT CASES ID(a) INDATUM(a) UTDATUM(a).
   NUMERIC CounterVTF(f3).
   IF $casenum = 1 or ID ne lag(ID) CounterVTF = 1.
   IF ID = lag(ID) CounterVTF = lag(CounterVTF) + 1.
      EXECUTE.

SORT CASES ID(a) CounterVTF(d).
EXECUTE.
*Skapa en rad per individ.
If ID = lag(ID) CytoAdm = CytoAdm + lag(CytoAdm).
IF CytoAdm > 0 CytoAdm = 1.
If ID = lag(ID) CytoAdm2 = CytoAdm2 + lag(CytoAdm2).
IF CytoAdm2 > 0 CytoAdm2 = 1.
If ID = lag(ID) Radiak = Radiak + lag(Radiak).
IF Radiak > 0 Radiak = 1.
If ID = lag(ID) Radiak2 = Radiak2 + lag(Radiak2).
IF Radiak2 > 0 Radiak2 = 1.
If ID = lag(ID) HD_Cron = HD_Cron + lag(HD_Cron).
IF HD_Cron > 0 HD_Cron = 1.
If ID = lag(ID) IHD = IHD + lag(IHD).
IF IHD > 0 IHD = 1.
If ID = lag(ID) OtherHD = OtherHD + lag(OtherHD).
IF OtherHD > 0 OtherHD = 1.
If ID = lag(ID) Hypertens = Hypertens + lag(Hypertens).
```

```
IF Hypertens > 0 Hypertens = 1.

If ID = lag(ID) Diabetes1 = Diabetes1 + lag(Diabetes1).

IF Diabetes1 > 0 Diabetes1 = 1.

If ID = lag(ID) Diabetes2 = Diabetes2 + lag(Diabetes2).

IF Diabetes2 > 0 Diabetes2 = 1.

If ID = lag(ID) Stroke = Stroke + lag(Stroke).

IF Stroke > 0 Stroke = 1.

If ID = lag(ID) Njursvikt = Njursvikt + lag(Njursvikt).

IF Njursvikt > 0 Njursvikt = 1.

If ID = lag(ID) N181 = N181 + lag(N181).

IF N181 > 0 N181 = 1.

If ID = lag(ID) N182 = N182 + lag(N182).

IF N182 > 0 N182 = 1.

If ID = lag(ID) N183 = N183 + lag(N183).

IF N183 > 0 N183 = 1.

If ID = lag(ID) N184 = N184 + lag(N184).

IF N184 > 0 N184 = 1.

If ID = lag(ID) N185 = N185 + lag(N185).

IF N185 > 0 N185 = 1.

If ID = lag(ID) N189 = N189 + lag(N189).

IF N189 > 0 N189 = 1.

If ID = lag(ID) DR016 = DR016 + lag(DR016).

IF DR016 > 0 DR016 = 1.

If ID = lag(ID) DR024 = DR024 + lag(DR024).

IF DR024 > 0 DR024 = 1.

If ID = lag(ID) KOL = KOL + lag(KOL).

IF KOL > 0 KOL = 1.

If ID = lag(ID) Astma = Astma + lag(Astma).

IF Astma > 0 Astma = 1.
```

```
If ID = lag(ID) Fetma = Fetma + lag(Fetma).

IF Fetma > 0 Fetma = 1.

If ID = lag(ID) Immunsupprimerad = Immunsupprimerad + lag(Immunsupprimerad).

IF Immunsupprimerad > 0 Immunsupprimerad = 1.

If ID = lag(ID) Cancer_dia = Cancer_dia + lag(Cancer_dia).

IF Cancer_dia > 0 Cancer_dia = 1.

If ID = lag(ID) BlodCancer = BlodCancer + lag(BlodCancer).

IF BlodCancer > 0 BlodCancer = 1.

If ID = lag(ID) Inflam = Inflam + lag(Inflam).

IF Inflam > 0 Inflam = 1.

If ID = lag(ID) Transplanterad = Transplanterad + lag(Transplanterad).

IF Transplanterad > 0 Transplanterad = 1.


*Spara ett vtf per individ.

  EXECUTE.

SELECT IF CounterVTF = 1.

  EXECUTE.


ADD FILES FILE*

  /KEEP ID CytoAdm to Transplanterad.

    EXECUTE.


SAVE OUTFILE 'Sepsis_Comorb.sav'.

  DATASET NAME Sepsis_Comorb.

GET FILE 'Sepsis_temp.sav'.

  DATASET NAME SIR WINDOW=FRONT.


DATASET ACTIVATE SIR.

MATCH FILES /FILE=*
```

```
  /FILE='Sepsis_Comorb'
  /BY ID.
EXECUTE.

DATASET ACTIVATE SIR.
MATCH FILES /FILE=*
  /TABLE='Sepsis_CCI'
  /BY ID.
EXECUTE.
    DATASET CLOSE Sepsis_CCI.
    DATASET CLOSE Sepsis_Comorb.
    DATASET ACTIVATE SIR.

RECODE CytoAdm to CCI (SYSMIS = 0) (ELSE = COPY).
    EXECUTE.
SORT CASES ID(a).


NUMERIC ARDS(f2).
NUMERIC COVID(f2).
NUMERIC COVID2(f2).
        VALUE LABELS Sepsis 0 'No sepsis' 1 'Sepsis'.
        VALUE LABELS ARDS 0 'No ARDS' 1 'ARDS'.
        VALUE LABELS Covid 0 'No Covid' 1 'Covid'.
        VARIABLE LABELS COVID2 'Covid också'.
    COMPUTE ARDS = 0.
    COMPUTE COVID = 0.
    COMPUTE COVID2 = 0.
    COMPUTE Sepsis = 1.
```

```
        EXECUTE.

SORT CASES ID(a) InskrivDatum(a) UtskrivDatum(a).
    NUMERIC CounterIVA(f3).
    IF $casenum = 1 or ID ne lag(ID) CounterIVA = 1.
    IF ID = lag(ID) CounterIVA = lag(CounterIVA) + 1.
        EXECUTE.
DESCRIPTIVES ID.
FREQUENCIES CounterIVA.

SAVE OUTFILE 'TEMP11.sav'.


    DATASET CLOSE all.
    GET FILE TEMP11.sav.
    DATASET NAME SIR WINDOW=FRONT.
SORT CASES ID.
Get file 'Y:\Original_SPSS\ut_par_sv_8996_2017.sav'.
DATASET NAME Soc WINDOW=FRONT.
DATASET ACTIVATE Soc.

RENAME VARIABLES (lopnr LKF Kon = ID L Kon).
    FORMATS ID(f10).
        EXECUTE.

STRING LKF(a10).
COMPUTE LKF = concat(rtrim(LK),' ',rtrim(L)).
    EXECUTE.
    DELETE VARIABLES LK L.
```

```
        EXECUTE.
SORT CASES ID(a) INDATUM(a) UTDATUM(a).
    NUMERIC CounterVTF(f3).
    IF $casenum = 1 or ID ne lag(ID) CounterVTF = 1.
    IF ID = lag(ID) CounterVTF = lag(CounterVTF) + 1.
        EXECUTE.
SELECT IF CounterVTF = 1.
EXECUTE.

ADD FILES FILE*
    /KEEP ID SENUTV.
        EXECUTE.
DATASET ACTIVATE SIR.
MATCH FILES /FILE=*
  /FILE='Soc'
  /BY ID.
EXECUTE.

SELECT IF Sepsis = 1.
EXECUTE.

DATASET CLOSE Soc.
    DATASET ACTIVATE Sir.
    DATASET NAME Sepsis.
    DATASET ACTIVATE Sepsis.

SELECT IF InskrivDatum >0.
    EXECUTE.
COMPUTE ID_Sepsis = ID.
```

```
   EXECUTE.
COMPUTE ID = $casenum + 200000.
   EXECUTE.

ADD FILES FILE*
/KEEP ID Alder Kon SENUTV InskrivDatum UtskrivDatum SjukhusTyp Opererad SAPS3Score SAPS3_u_comorb SAPS3ScoreBoxIII
Operationstyp APACHE_Score APACHE_AkutFysPoang IMV_tid CRRT_tid Sepsis_grad IMV CRRT ICU_LoS
CytoAdm CytoAdm2 Radiak Radiak2 HD_Cron IHD OtherHD Hypertens Diabetes1 Diabetes2 Stroke Njursvikt N181 N182 N183 N184 N185
N189 DR016 DR024 KOL Astma Fetma Immunsupprimerad Cancer_dia BlodCancer Inflam Transplanterad
CCI COVID COVID2 Sepsis ARDS DeadICU_n DeathDate_ICU Dead_n DeathDate ID_Sepsis.
   EXECUTE.

DATASET ACTIVATE Sepsis.
RENAME VARIABLES (InskrivDatum Alder = InskrDat Age).
   EXECUTE.
NUMERIC Sex(f1).
   VARIABLE LABELS Sex 'Sex at ICU admission'.
   VALUE LABELS Sex 1 'Man' 2 'Kvinna'.
   IF Kon = 'M' Sex = 1.
   IF Kon = 'K' Sex = 2.
FORMATS Age(f3).
   VARIABLE LABELS Age 'Age at ICU admission'.
IF Opererad = 'Ja-elektivt' Opererad = 'Ja-elek'.
IF Opererad = 'Ja-akut' Opererad = 'Ja-akut'.
ALTER TYPE Opererad(a7).
   EXECUTE.
```

```
DESCRIPTIVES ID.

FREQUENCIES Sepsis.

SELECT IF Age > 17.

  EXECUTE.

DESCRIPTIVES ID.


FREQUENCIES COVID COVID2 Sepsis ARDS.


SAVE OUTFILE 'Sepsis_komplett.sav'.




*////////////////////////////////////////////////////////////////////////////////////////////////////////////////////
/////////////.


*ARDS.Sortera bort IVA före ARDS och ej ARDS.

Get file SIR_temp2.sav.

  DATASET NAME ARDS WINDOW = FRONT.

  DATASET ACTIVATE ARDS.


*ARDS!. Sortera bort IVA fötr ARDS och ej ARDS.

SELECT IF A = 1.

EXECUTE.


DELETE VARIABLES CounterIVA.

SORT CASES ID(a) InskrivDatum(a) UtskrivDatum(a).

  NUMERIC CounterIVA(f3).

  IF $casenum = 1 or ID ne lag(ID) CounterIVA = 1.

  IF ID = lag(ID) CounterIVA = lag(CounterIVA) + 1.

    EXECUTE.

FREQUENCIES CounterIVA ARDS.
```

```
*diff är dagar mellan intensivvårdstillfällena. 0 eller 1 dags skillnad innebär samma vtf.
Compute diff = datediff(InskrTid,lag(UtskrTid),'hours').
Compute diff = Diff / 24.
  VARIABLE LABELS diff ' days from out previous to in actual ICU episode'.
  IF ID ne lag(ID) diff = $sysmis.
  IF CounterIVA = 1 X = 1.
  IF lag(X) = 1 and diff < 1 X = 1.
    EXECUTE.
SELECT IF X = 1.
  EXECUTE.

DELETE VARIABLES CounterIVA.
SORT CASES ID(a) InskrivDatum(a) UtskrivDatum(a).
  NUMERIC CounterIVA(f3).
  IF $casenum = 1 or ID ne lag(ID) CounterIVA = 1.
  IF ID = lag(ID) CounterIVA = lag(CounterIVA) + 1.
    EXECUTE.
FREQUENCIES CounterIVA ARDS.

*Skapa och spara ett vtf.
DELETE VARIABLES CounterIVA.
  EXECUTE.
SORT CASES ID(a) InskrTid(a) UtskrTid(a).
NUMERIC CounterIVA(f3).
  IF $casenum = 1 or ID ne lag(ID) CounterIVA = 1.
  IF ID = lag(ID) CounterIVA = lag(CounterIVA) + 1.
  EXECUTE.
```

```
*Åtgärder.


*Lägg ihop mot första vårdtillfället.
SORT CASES ID(a) CounterIVA(d).
   EXECUTE.
IF ID = lag(ID) CRRT_tid = CRRT_tid + lag(CRRT_tid).
IF ID = lag(ID) IMV_tid = IMV_tid + lag(IMV_tid).
IF ID = lag(ID) and lag(Avliden_IVA) = 'Ja' Avliden_IVA = 'Ja'.
IF ID = lag(ID) UtskrivDatum = lag(UtskrivDatum).
IF ID = lag(ID) UtskrTid = lag(UtskrTid).
   EXECUTE.
SELECT IF CounterIVA = 1.
   EXECUTE.
FREQUENCIES CounterIVA.


NUMERIC IMV(f2).
   VALUE LABELS IMV 1 'IMV' 0 'No IMV'.
NUMERIC CRRT(f2).
   VALUE LABELS CRRT 1 'CRRT' 0 'No CRRT'.
   IF IMV_tid > 0 IMV = 1.
   IF sysmis(IMV) IMV = 0.
   IF CRRT_tid > 0 CRRT = 1.
   IF sysmis(CRRT) CRRT = 0.
      EXECUTE.


NUMERIC ICU_LoS(f6).
      VARIABLE LABELS ICU_LoS 'Total sammanhängande vårdtid på IVA, dygn.'.
   COMPUTE ICU_LoS = datediff(UtskrTid,InskrTid,'hours').
   COMPUTE ICU_LoS = ICU_LoS/24.
```

```
   EXECUTE.
NUMERIC Dead_ICU_n(f1).
   IF Avliden_IVA = 'Ja' DeadICU_n = 1.
   IF sysmis(DeadICU_n) DeadICU_n = 0.
IF DeadICU_n = 1 DeathDate_ICU = UtskrivDatum.
   FORMATS DeathDate_ICU(date11).
NUMERIC Dead_n(f2).
   IF Avliden = 'Ja' Dead_n = 1.
   IF sysmis(Dead_n) Dead_n = 0.
IF Dead_n = 1 DeathDate = AvregDatum.
   FORMATS DeathDate(date11).

   ADD FILES FILE*
   /KEEP ID InskrTid UtskrTid InskrivDatum UtskrivDatum DeadICU_n DeathDate_ICU Dead_n DeathDate
SjukhusTyp Alder Kon Opererad SAPS3Score SAPS3_u_comorb
   SAPS3ScoreBoxIII Operationstyp APACHE_Score APACHE_AkutFysPoang Status IMV_tid CRRT_tid IMV CRRT
ICU_LoS ARDS.
      EXECUTE.

SAVE OUTFILE 'ARDS_temp.sav'.
*lägg till CCI och diagnoser.
DATASET CLOSE ARDS.
GET FILE 'ARDS_temp.sav'.
   DATASET NAME ARDS WINDOW=FRONT.

Get file 'Y:\Original_SPSS\ut_par_sv_8996_2017.sav'.
DATASET NAME Soc WINDOW=FRONT.
DATASET ACTIVATE Soc.
```

```
RENAME VARIABLES (lopnr LKF Kon = ID L Kon).
  FORMATS ID(f10).
    EXECUTE.
DELETE VARIABLES KON FODDAT ALDER to UTDATUMA UTDATUMA PVARD HDIA DIA_ANT SENUTV drg
SJUKHUSNAMN.
  EXECUTE.
STRING LKF(a10).
COMPUTE LKF = concat(rtrim(LK),' ',rtrim(L)).
  EXECUTE.
  DELETE VARIABLES LK L.
    EXECUTE.
SORT CASES ID(a) INDATUM(a) UTDATUM(a).
  NUMERIC CounterVTF(f3).
  IF $casenum = 1 or ID ne lag(ID) CounterVTF = 1.
  IF ID = lag(ID) CounterVTF = lag(CounterVTF) + 1.
    EXECUTE.

DATASET ACTIVATE Soc.
MATCH FILES /FILE=*
 /TABLE='ARDS'
 /BY ID.
EXECUTE.

DATASET CLOSE ARDS.
  DATASET ACTIVATE Soc.
  DATASET NAME ARDS.
  DATASET ACTIVATE ARDS.

SELECT IF InskrTid >0.
```

```
   EXECUTE.

DELETE VARIABLES CounterVTF.
   EXECUTE.
SORT CASES ID(a) INDATUM(a) UTDATUM(a).
   NUMERIC CounterVTF(f3).
   IF $casenum = 1 or ID ne lag(ID) CounterVTF = 1.
   IF ID = lag(ID) CounterVTF = lag(CounterVTF) + 1.
      EXECUTE.


SAVE outfile 'ARDS_soc.sav'.
*    GET FILE 'ARDS_soc.sav'.
*    DATASET NAME ARDS WINDOW=FRONT.
*    DATASET ACTIVATE ARDS.
*Skapa comorbiditetsvariabeln.

*Skapa comorbiditetsvariabeln.
DATASET NAME ARDS_CCI.

SELECT IF INDATUM < InskrivDatum.
   EXECUTE.

DELETE VARIABLES CounterVTF.
   EXECUTE.
SORT CASES ID(a) INDATUM(a) UTDATUM(a).
   NUMERIC CounterVTF(f3).
   IF $casenum = 1 or ID ne lag(ID) CounterVTF = 1.
   IF ID = lag(ID) CounterVTF = lag(CounterVTF) + 1.
```

```
    EXECUTE.

ADD FILES FILE*/KEEP ID CounterVTF Diagnos.
  EXECUTE.

SORT CASES BY ID CounterVTF.
CASESTOVARS
 /ID=ID
 /INDEX=CounterVTF
 /GROUPBY=VARIABLE.

*Slå ihop diagnosvariablerna.
STRING v(A2100).
VECTOR v=DIAGNOS.1 TO DIAGNOS.172.
LOOP cnt=1 TO 172.
- COMPUTE v=CONCAT(RTRIM(v)," ",v(cnt)," ") .
END LOOP.
- COMPUTE v=CONCAT(RTRIM(v),v(172)) .
  EXECUTE.
VARIABLE WIDTH  v (15).
RENAME VARIABLES v = Comorbidity_IVA.
VARIABLE LABELS Comorbidity_IVA 'Comorbiditet till och med vtf före IVA'.

*Räkna tecken i Comorbidity_IVA så att Kolla att diagnoserna får plats (2090).
compute count_Comorbidity_IVA=char.length(Comorbidity_IVA).
  EXECUTE.
DESCRIPTIVES VARIABLES=count_Comorbidity_IVA
 /STATISTICS=MEAN MIN MAX.
```

```
*Ta bort överblivna variabler.

ADD FILES FILE*/KEEP ID Comorbidity_IVA.
  EXECUTE.

RENAME VARIABLES (Comorbidity_IVA = ICD).

*Create CCI according to Quan.
NUMERIC CCI_1(f2).
NUMERIC CCI_2(f2).
NUMERIC CCI_3(f2).
NUMERIC CCI_4(f2).
NUMERIC CCI_5(f2).
NUMERIC CCI_6(f2).
NUMERIC CCI_7(f2).
NUMERIC CCI_8(f2).
NUMERIC CCI_9(f2).
NUMERIC CCI_10(f2).
NUMERIC CCI_11(f2).
NUMERIC CCI_12(f2).
NUMERIC CCI_13(f2).
NUMERIC CCI_14(f2).
NUMERIC CCI_15(f2).
NUMERIC CCI_16(f2).
NUMERIC CCI_17(f2).
Numeric CCI(f8).
  VARIABLE LABELS CCI 'CCI according to Quan'.

*The code will give CCI.
```

```
*CCI 1.
COMPUTE CCI_1 =
   (CHAR.INDEX(UPCASE(ICD),'I21') > 0) OR
   (CHAR.INDEX(UPCASE(ICD),'I22') > 0) OR
   (CHAR.INDEX(UPCASE(ICD),'I252') > 0).

      EXECUTE.
   VARIABLE LABELS CCI_1 'Myocardial Infarction'.
   VALUE LABELS CCI_1 0 'no myocardial infarction' 1 'myocardial infarction'.

*CCI 2.
COMPUTE CCI_2 =
    (CHAR.INDEX(UPCASE(ICD),'I43') > 0) OR
    (CHAR.INDEX(UPCASE(ICD),'I50') > 0) OR
    (CHAR.INDEX(UPCASE(ICD),'I099') > 0) OR
    (CHAR.INDEX(UPCASE(ICD),'I110') > 0) OR
    (CHAR.INDEX(UPCASE(ICD),'I130') > 0) OR
    (CHAR.INDEX(UPCASE(ICD),'I132') > 0) OR
    (CHAR.INDEX(UPCASE(ICD),'I255') > 0) OR
    (CHAR.INDEX(UPCASE(ICD),'I420') > 0) OR
    (CHAR.INDEX(UPCASE(ICD),'I425') > 0) OR
    (CHAR.INDEX(UPCASE(ICD),'I426') > 0) OR
    (CHAR.INDEX(UPCASE(ICD),'I427') > 0) OR
    (CHAR.INDEX(UPCASE(ICD),'I428') > 0) OR
    (CHAR.INDEX(UPCASE(ICD),'I429') > 0) OR
    (CHAR.INDEX(UPCASE(ICD),'P290') > 0).

      EXECUTE.
   VARIABLE LABELS CCI_2 'Congestive Heart Failure'.
```

```
VALUE LABELS CCI_2 0 'no Congestive Heart Failure' 1 'Congestive Heart Failure'.


*CCI 3.
COMPUTE CCI_3 =
  (CHAR.INDEX(UPCASE(ICD),'I70') > 0) OR
  (CHAR.INDEX(UPCASE(ICD),'I71') > 0) OR
  (CHAR.INDEX(UPCASE(ICD),'I731') > 0) OR
  (CHAR.INDEX(UPCASE(ICD),'I738') > 0) OR
  (CHAR.INDEX(UPCASE(ICD),'I739') > 0) OR
  (CHAR.INDEX(UPCASE(ICD),'I771') > 0) OR
  (CHAR.INDEX(UPCASE(ICD),'I790') > 0) OR
  (CHAR.INDEX(UPCASE(ICD),'I792') > 0) OR
  (CHAR.INDEX(UPCASE(ICD),'K551') > 0) OR
  (CHAR.INDEX(UPCASE(ICD),'K558') > 0) OR
  (CHAR.INDEX(UPCASE(ICD),'K559') > 0) OR
  (CHAR.INDEX(UPCASE(ICD),'Z958') > 0) OR
  (CHAR.INDEX(UPCASE(ICD),'Z959') > 0).

    EXECUTE.
  VARIABLE LABELS CCI_3 'Peripheral Vascular Disease'.
  VALUE LABELS CCI_3 0 'no Peripheral Vascular Disease' 1 'Peripheral Vascular Disease'.


*CCI 4.
COMPUTE CCI_4 =
  (CHAR.INDEX(UPCASE(ICD),'G45') > 0) OR
  (CHAR.INDEX(UPCASE(ICD),'G46') > 0) OR
  (CHAR.INDEX(UPCASE(ICD),'H340') > 0) OR
  (CHAR.INDEX(UPCASE(ICD),'I6') > 0).
```

```
    EXECUTE.
  VARIABLE LABELS CCI_4 'Cerebrovascular Disease'.
  VALUE LABELS CCI_4 0 'no Cerebrovascular Disease' 1 'Cerebrovascular Disease'.


*CCI 5.
COMPUTE CCI_5 =
  (CHAR.INDEX(UPCASE(ICD),'F00') > 0) OR
  (CHAR.INDEX(UPCASE(ICD),'F01') > 0) OR
  (CHAR.INDEX(UPCASE(ICD),'F02') > 0) OR
  (CHAR.INDEX(UPCASE(ICD),'F03') > 0) OR
  (CHAR.INDEX(UPCASE(ICD),'F051') > 0) OR
  (CHAR.INDEX(UPCASE(ICD),'G30') > 0) OR
  (CHAR.INDEX(UPCASE(ICD),'G311') > 0).

    EXECUTE.
VARIABLE LABELS CCI_5 'Dementia'.
VALUE LABELS CCI_5 0 'no Dementia' 1 'Dementia'.

*CCI 6.
COMPUTE CCI_6 =
  (CHAR.INDEX(UPCASE(ICD),'I278') > 0) OR
  (CHAR.INDEX(UPCASE(ICD),'I279') > 0) OR
  (CHAR.INDEX(UPCASE(ICD),'IJ40') > 0) OR
  (CHAR.INDEX(UPCASE(ICD),'J41') > 0) OR
  (CHAR.INDEX(UPCASE(ICD),'J42') > 0) OR
  (CHAR.INDEX(UPCASE(ICD),'J43') > 0) OR
  (CHAR.INDEX(UPCASE(ICD),'J44') > 0) OR
  (CHAR.INDEX(UPCASE(ICD),'J45') > 0) OR
```

```
    (CHAR.INDEX(UPCASE(ICD),'J46') > 0) OR
    (CHAR.INDEX(UPCASE(ICD),'J47') > 0) OR
    (CHAR.INDEX(UPCASE(ICD),'J60') > 0) OR
    (CHAR.INDEX(UPCASE(ICD),'J61') > 0) OR
    (CHAR.INDEX(UPCASE(ICD),'J62') > 0) OR
    (CHAR.INDEX(UPCASE(ICD),'J63') > 0) OR
    (CHAR.INDEX(UPCASE(ICD),'J64') > 0) OR
    (CHAR.INDEX(UPCASE(ICD),'J65') > 0) OR
    (CHAR.INDEX(UPCASE(ICD),'J66') > 0) OR
    (CHAR.INDEX(UPCASE(ICD),'J67') > 0) OR
    (CHAR.INDEX(UPCASE(ICD),'J684') > 0) OR
    (CHAR.INDEX(UPCASE(ICD),'J701') > 0) OR
    (CHAR.INDEX(UPCASE(ICD),'J703') > 0).

      EXECUTE.
VARIABLE LABELS CCI_6 'Chronic Pulmonary Disease'.
VALUE LABELS CCI_6 0 'no Chronic Pulmonary Disease' 1 'Chronic Pulmonary Disease'.

*CCI 7.
COMPUTE CCI_7 =
    (CHAR.INDEX(UPCASE(ICD),'M05') > 0) OR
    (CHAR.INDEX(UPCASE(ICD),'M06') > 0) OR
    (CHAR.INDEX(UPCASE(ICD),'M315') > 0) OR
    (CHAR.INDEX(UPCASE(ICD),'M32') > 0) OR
    (CHAR.INDEX(UPCASE(ICD),'M33') > 0) OR
    (CHAR.INDEX(UPCASE(ICD),'M34') > 0) OR
    (CHAR.INDEX(UPCASE(ICD),'M351') > 0) OR
    (CHAR.INDEX(UPCASE(ICD),'M353') > 0) OR
    (CHAR.INDEX(UPCASE(ICD),'M360') > 0) OR
```

```
   (CHAR.INDEX(UPCASE(ICD),'J47') > 0).


      EXECUTE.
VARIABLE LABELS CCI_7 'Connective Tissue Disease-Rheumatic Disease'.
VALUE LABELS CCI_7 0 'no Connective Tissue Disease-Rheumatic Disease' 1 'Connective Tissue Disease-Rheumatic Disease'.


*CCI 8.
COMPUTE CCI_8 =
   (CHAR.INDEX(UPCASE(ICD),'K25') > 0) OR
   (CHAR.INDEX(UPCASE(ICD),'K26') > 0) OR
   (CHAR.INDEX(UPCASE(ICD),'K27') > 0) OR
   (CHAR.INDEX(UPCASE(ICD),'K28') > 0).


      EXECUTE.
VARIABLE LABELS CCI_8 'Peptic Ulcer Disease'.
VALUE LABELS CCI_8 0 'no Peptic Ulcer Disease' 1 'Peptic Ulcer Disease'.


*CCI 9.
COMPUTE CCI_9 = (CHAR.INDEX(UPCASE(ICD),'B18') > 0) OR
   (CHAR.INDEX(UPCASE(ICD),'K700') > 0) OR
   (CHAR.INDEX(UPCASE(ICD),'K701') > 0) OR
   (CHAR.INDEX(UPCASE(ICD),'K702') > 0) OR
   (CHAR.INDEX(UPCASE(ICD),'K703') > 0) OR
   (CHAR.INDEX(UPCASE(ICD),'K709') > 0) OR
   (CHAR.INDEX(UPCASE(ICD),'K713') > 0) OR
   (CHAR.INDEX(UPCASE(ICD),'K714') > 0) OR
   (CHAR.INDEX(UPCASE(ICD),'K715') > 0) OR
   (CHAR.INDEX(UPCASE(ICD),'K717') > 0) OR
   (CHAR.INDEX(UPCASE(ICD),'K73') > 0) OR
```

```
    (CHAR.INDEX(UPCASE(ICD),'K74') > 0) OR
    (CHAR.INDEX(UPCASE(ICD),'K760') > 0) OR
    (CHAR.INDEX(UPCASE(ICD),'K762') > 0) OR
    (CHAR.INDEX(UPCASE(ICD),'K763') > 0) OR
    (CHAR.INDEX(UPCASE(ICD),'K764') > 0) OR
    (CHAR.INDEX(UPCASE(ICD),'K768') > 0) OR
    (CHAR.INDEX(UPCASE(ICD),'K768') > 0) OR
    (CHAR.INDEX(UPCASE(ICD),'Z944') > 0).

        EXECUTE.
VARIABLE LABELS CCI_9 'Mild Liver Disease'.
VALUE LABELS CCI_9 0 'no Mild Liver Disease' 1 'Mild Liver Disease'.

*CCI 10.
COMPUTE CCI_10 =
    (CHAR.INDEX(UPCASE(ICD),'E100') > 0) OR
    (CHAR.INDEX(UPCASE(ICD),'E101') > 0) OR
    (CHAR.INDEX(UPCASE(ICD),'E106') > 0) OR
    (CHAR.INDEX(UPCASE(ICD),'E108') > 0) OR
    (CHAR.INDEX(UPCASE(ICD),'E109') > 0) OR
    (CHAR.INDEX(UPCASE(ICD),'E110') > 0) OR
    (CHAR.INDEX(UPCASE(ICD),'E111') > 0) OR
    (CHAR.INDEX(UPCASE(ICD),'E116') > 0) OR
    (CHAR.INDEX(UPCASE(ICD),'E118') > 0) OR
    (CHAR.INDEX(UPCASE(ICD),'E119') > 0) OR
    (CHAR.INDEX(UPCASE(ICD),'E120') > 0) OR
    (CHAR.INDEX(UPCASE(ICD),'E121') > 0) OR
    (CHAR.INDEX(UPCASE(ICD),'E126') > 0) OR
    (CHAR.INDEX(UPCASE(ICD),'E128') > 0) OR
```

```
   (CHAR.INDEX(UPCASE(ICD),'E129') > 0) OR
   (CHAR.INDEX(UPCASE(ICD),'E130') > 0) OR
   (CHAR.INDEX(UPCASE(ICD),'E131') > 0) OR
   (CHAR.INDEX(UPCASE(ICD),'E136') > 0) OR
   (CHAR.INDEX(UPCASE(ICD),'E138') > 0) OR
   (CHAR.INDEX(UPCASE(ICD),'E139') > 0) OR
   (CHAR.INDEX(UPCASE(ICD),'E140') > 0) OR
   (CHAR.INDEX(UPCASE(ICD),'E141') > 0) OR
   (CHAR.INDEX(UPCASE(ICD),'E146') > 0) OR
   (CHAR.INDEX(UPCASE(ICD),'E148') > 0) OR
   (CHAR.INDEX(UPCASE(ICD),'E149') > 0).

      EXECUTE.
VARIABLE LABELS CCI_10 'Diabetes without complications'.
VALUE LABELS CCI_10 0 'no Diabetes without complications' 1 'Diabetes without complications'.

*CCI 11.
COMPUTE CCI_11 =
   (CHAR.INDEX(UPCASE(ICD),'E102') > 0) OR
   (CHAR.INDEX(UPCASE(ICD),'E103') > 0) OR
   (CHAR.INDEX(UPCASE(ICD),'E104') > 0) OR
   (CHAR.INDEX(UPCASE(ICD),'E105') > 0) OR
   (CHAR.INDEX(UPCASE(ICD),'E107') > 0) OR
   (CHAR.INDEX(UPCASE(ICD),'E112') > 0) OR
   (CHAR.INDEX(UPCASE(ICD),'E113') > 0) OR
   (CHAR.INDEX(UPCASE(ICD),'E114') > 0) OR
   (CHAR.INDEX(UPCASE(ICD),'E115') > 0) OR
   (CHAR.INDEX(UPCASE(ICD),'E117') > 0) OR
   (CHAR.INDEX(UPCASE(ICD),'E122') > 0) OR
```

```
    (CHAR.INDEX(UPCASE(ICD),'E123') > 0) OR
    (CHAR.INDEX(UPCASE(ICD),'E124') > 0) OR
    (CHAR.INDEX(UPCASE(ICD),'E125') > 0) OR
    (CHAR.INDEX(UPCASE(ICD),'E127') > 0) OR
    (CHAR.INDEX(UPCASE(ICD),'E132') > 0) OR
    (CHAR.INDEX(UPCASE(ICD),'E133') > 0) OR
    (CHAR.INDEX(UPCASE(ICD),'E134') > 0) OR
    (CHAR.INDEX(UPCASE(ICD),'E135') > 0) OR
    (CHAR.INDEX(UPCASE(ICD),'E137') > 0) OR
    (CHAR.INDEX(UPCASE(ICD),'E142') > 0) OR
    (CHAR.INDEX(UPCASE(ICD),'E143') > 0) OR
    (CHAR.INDEX(UPCASE(ICD),'E144') > 0) OR
    (CHAR.INDEX(UPCASE(ICD),'E145') > 0) OR
    (CHAR.INDEX(UPCASE(ICD),'E147') > 0).

      EXECUTE.
VARIABLE LABELS CCI_11 'Diabetes with chronic complication'.
VALUE LABELS CCI_11 0 'no Diabetes with chronic complication' 1 'Diabetes with chronic complication'.

*CCI 12.
COMPUTE CCI_12 =
    (CHAR.INDEX(UPCASE(ICD),'G041') > 0) OR
    (CHAR.INDEX(UPCASE(ICD),'G114') > 0) OR
    (CHAR.INDEX(UPCASE(ICD),'G801') > 0) OR
    (CHAR.INDEX(UPCASE(ICD),'G802') > 0) OR
    (CHAR.INDEX(UPCASE(ICD),'G81') > 0) OR
    (CHAR.INDEX(UPCASE(ICD),'G82') > 0) OR
    (CHAR.INDEX(UPCASE(ICD),'G830') > 0) OR
    (CHAR.INDEX(UPCASE(ICD),'G831') > 0) OR
```

```
   (CHAR.INDEX(UPCASE(ICD),'G832') > 0) OR
   (CHAR.INDEX(UPCASE(ICD),'G833') > 0) OR
   (CHAR.INDEX(UPCASE(ICD),'G834') > 0) OR
   (CHAR.INDEX(UPCASE(ICD),'G839') > 0).

      EXECUTE.
VARIABLE LABELS CCI_12 'Hemiplegia or paraplegia'.
VALUE LABELS CCI_12 0 'no Hemiplegia or paraplegia' 1 'Hemiplegia or paraplegia'.

*CCI 13.
COMPUTE CCI_13 =
   (CHAR.INDEX(UPCASE(ICD),'I120') > 0) OR
   (CHAR.INDEX(UPCASE(ICD),'I131') > 0) OR
   (CHAR.INDEX(UPCASE(ICD),'N032') > 0) OR
   (CHAR.INDEX(UPCASE(ICD),'N033') > 0) OR
   (CHAR.INDEX(UPCASE(ICD),'N034') > 0) OR
   (CHAR.INDEX(UPCASE(ICD),'N035') > 0) OR
   (CHAR.INDEX(UPCASE(ICD),'N036') > 0) OR
   (CHAR.INDEX(UPCASE(ICD),'N037') > 0) OR
   (CHAR.INDEX(UPCASE(ICD),'N052') > 0) OR
   (CHAR.INDEX(UPCASE(ICD),'N053') > 0) OR
   (CHAR.INDEX(UPCASE(ICD),'N054') > 0) OR
   (CHAR.INDEX(UPCASE(ICD),'N055') > 0) OR
   (CHAR.INDEX(UPCASE(ICD),'N056') > 0) OR
   (CHAR.INDEX(UPCASE(ICD),'N057') > 0) OR
   (CHAR.INDEX(UPCASE(ICD),'N18') > 0) OR
   (CHAR.INDEX(UPCASE(ICD),'N19') > 0) OR
   (CHAR.INDEX(UPCASE(ICD),'N250') > 0) OR
   (CHAR.INDEX(UPCASE(ICD),'Z490') > 0) OR
```

```
   (CHAR.INDEX(UPCASE(ICD),'Z491') > 0) OR
   (CHAR.INDEX(UPCASE(ICD),'Z492') > 0) OR
   (CHAR.INDEX(UPCASE(ICD),'Z940') > 0) OR
   (CHAR.INDEX(UPCASE(ICD),'Z992') > 0).

     EXECUTE.
VARIABLE LABELS CCI_13 'Renal disease'.
VALUE LABELS CCI_13 0 'no Renal disease' 1 'Renal disease'.

*CCI 14.
COMPUTE CCI_14 =
  (CHAR.INDEX(UPCASE(ICD),'C0') > 0) OR
  (CHAR.INDEX(UPCASE(ICD),'C30') > 0) OR
  (CHAR.INDEX(UPCASE(ICD),'C31') > 0) OR
  (CHAR.INDEX(UPCASE(ICD),'C32') > 0) OR
  (CHAR.INDEX(UPCASE(ICD),'C33') > 0) OR
  (CHAR.INDEX(UPCASE(ICD),'C34') > 0) OR
  (CHAR.INDEX(UPCASE(ICD),'C37') > 0) OR
  (CHAR.INDEX(UPCASE(ICD),'C38') > 0) OR
  (CHAR.INDEX(UPCASE(ICD),'C39') > 0) OR
  (CHAR.INDEX(UPCASE(ICD),'C40') > 0) OR
  (CHAR.INDEX(UPCASE(ICD),'C41') > 0) OR
  (CHAR.INDEX(UPCASE(ICD),'C43') > 0) OR
  (CHAR.INDEX(UPCASE(ICD),'C45') > 0) OR
  (CHAR.INDEX(UPCASE(ICD),'C46') > 0) OR
  (CHAR.INDEX(UPCASE(ICD),'C47') > 0) OR
  (CHAR.INDEX(UPCASE(ICD),'C48') > 0) OR
  (CHAR.INDEX(UPCASE(ICD),'C49') > 0) OR
  (CHAR.INDEX(UPCASE(ICD),'C50') > 0) OR
```

```
(CHAR.INDEX(UPCASE(ICD),'C51') > 0) OR
(CHAR.INDEX(UPCASE(ICD),'C52') > 0) OR
(CHAR.INDEX(UPCASE(ICD),'C53') > 0) OR
(CHAR.INDEX(UPCASE(ICD),'C54') > 0) OR
(CHAR.INDEX(UPCASE(ICD),'C55') > 0) OR
(CHAR.INDEX(UPCASE(ICD),'C56') > 0) OR
(CHAR.INDEX(UPCASE(ICD),'C57') > 0) OR
(CHAR.INDEX(UPCASE(ICD),'C58') > 0) OR
(CHAR.INDEX(UPCASE(ICD),'C6') > 0) OR
(CHAR.INDEX(UPCASE(ICD),'C70') > 0) OR
(CHAR.INDEX(UPCASE(ICD),'C71') > 0) OR
(CHAR.INDEX(UPCASE(ICD),'C72') > 0) OR
(CHAR.INDEX(UPCASE(ICD),'C73') > 0) OR
(CHAR.INDEX(UPCASE(ICD),'C74') > 0) OR
(CHAR.INDEX(UPCASE(ICD),'C75') > 0) OR
(CHAR.INDEX(UPCASE(ICD),'C76') > 0) OR
(CHAR.INDEX(UPCASE(ICD),'C81') > 0) OR
(CHAR.INDEX(UPCASE(ICD),'C82') > 0) OR
(CHAR.INDEX(UPCASE(ICD),'C83') > 0) OR
(CHAR.INDEX(UPCASE(ICD),'C84') > 0) OR
(CHAR.INDEX(UPCASE(ICD),'C85') > 0) OR
(CHAR.INDEX(UPCASE(ICD),'C88') > 0) OR
(CHAR.INDEX(UPCASE(ICD),'C90') > 0) OR
(CHAR.INDEX(UPCASE(ICD),'C91') > 0) OR
(CHAR.INDEX(UPCASE(ICD),'C92') > 0) OR
(CHAR.INDEX(UPCASE(ICD),'C93') > 0) OR
(CHAR.INDEX(UPCASE(ICD),'C94') > 0) OR
(CHAR.INDEX(UPCASE(ICD),'C95') > 0) OR
(CHAR.INDEX(UPCASE(ICD),'C96') > 0) OR
```

```
(CHAR.INDEX(UPCASE(ICD),'C97') > 0).

    EXECUTE.
 VARIABLE LABELS CCI_14 'Any malignancy, including lymphoma and leukemia, except malignant neoplasm of skin'.
 VALUE LABELS CCI_14 0 'no Any malignancy, including lymphoma and leukemia, except malignant neoplasm of skin' 1 'Any malignancy, including lymphoma and leukemia, except malignant neoplasm of skin'.

*CCI 15.
COMPUTE CCI_15 =
 (CHAR.INDEX(UPCASE(ICD),'I850') > 0) OR
 (CHAR.INDEX(UPCASE(ICD),'I859') > 0) OR
 (CHAR.INDEX(UPCASE(ICD),'I864') > 0) OR
 (CHAR.INDEX(UPCASE(ICD),'I982') > 0) OR
 (CHAR.INDEX(UPCASE(ICD),'K704') > 0) OR
 (CHAR.INDEX(UPCASE(ICD),'K711') > 0) OR
 (CHAR.INDEX(UPCASE(ICD),'K721') > 0) OR
 (CHAR.INDEX(UPCASE(ICD),'K729') > 0) OR
 (CHAR.INDEX(UPCASE(ICD),'K765') > 0) OR
 (CHAR.INDEX(UPCASE(ICD),'K766') > 0) OR
 (CHAR.INDEX(UPCASE(ICD),'K767') > 0).

    EXECUTE.
 VARIABLE LABELS CCI_15 'Moderate or severe liver disease'.
 VALUE LABELS CCI_15 0 'no Moderate or severe liver disease' 1 'Moderate or severe liver disease'.

*CCI 16.
COMPUTE CCI_16 =
 (CHAR.INDEX(UPCASE(ICD),'C77') > 0) OR
 (CHAR.INDEX(UPCASE(ICD),'C78') > 0) OR
```

```
   (CHAR.INDEX(UPCASE(ICD),'C79') > 0) OR

   (CHAR.INDEX(UPCASE(ICD),'C80')  > 0).


      EXECUTE.

   VARIABLE LABELS CCI_16 'Metastatic solid tumor'.

   VALUE LABELS CCI_16 0 'no Metastatic solid tumor' 1 'Metastatic solid tumor'.


*CCI 17.

COMPUTE CCI_17 =

   (CHAR.INDEX(UPCASE(ICD),'B20') > 0) OR

   (CHAR.INDEX(UPCASE(ICD),'B21') > 0) OR

   (CHAR.INDEX(UPCASE(ICD),'B22') > 0) OR

   (CHAR.INDEX(UPCASE(ICD),'B24') > 0).


      EXECUTE.

VARIABLE LABELS CCI_17 'AIDS/HIV'.

VALUE LABELS CCI_17 0 'no AIDS/HIV' 1 'AIDS/HIV'.


RECODE CCI_1 to CCI_17 (0=0) (Else = 1).

   EXECUTE.

*Add up CCI.

COMPUTE CCI = 0.

If CCI_1 > 0 CCI = CCI + 0.

If CCI_2 > 0 CCI = CCI + 2.

If CCI_3 > 0 CCI = CCI + 0.

If CCI_4 > 0 CCI = CCI + 0.

If CCI_5 > 0 CCI = CCI + 2.

If CCI_6 > 0 CCI = CCI + 1.

If CCI_7 > 0 CCI = CCI + 1.
```

```
If CCI_8 > 0 CCI = CCI + 0.

If CCI_9 > 0 CCI = CCI + 2.

If CCI_10 > 0 CCI = CCI + 0.

If CCI_11 > 0 CCI = CCI + 1.

If CCI_12 > 0 CCI = CCI + 2.

If CCI_13 > 0 CCI = CCI + 1.

If CCI_14 > 0 CCI = CCI + 2.

If CCI_15 > 0 CCI = CCI + 4.

If CCI_16 > 0 CCI = CCI + 6.

If CCI_17 > 0 CCI = CCI + 4.


  EXECUTE.


*Subtract mild liver and tumor disease from those with severe disease.

IF CCI_9 > 0 and CCI_15 > 0 CCI = CCI - 2.

IF CCI_14 > 0 and CCI_16 > 0 CCI = CCI - 2.

  EXECUTE.


  FREQUENCIES CCI_1 to CCI.


SAVE OUTFILE 'ARDS_CCI.sav'.

ADD FILES FILE*/KEEP ID CCI.

  EXECUTE.


GET FILE 'ARDS_soc.sav'.

DATASET NAME ARDS WINDOW=FRONT.

DATASET ACTIVATE ARDS.
```

```
*Hitta komorbiditeterna.
NUMERIC Tid_Diagn_Inkl(f6).
      VARIABLE LABELS Tid_Diagn_Inkl 'Tid från diagnos till inklusion'.
COMPUTE Tid_Diagn_Inkl = datediff(InskrivDatum,UTDATUM,'days').
   EXECUTE.

NUMERIC Tid_Atg_Inkl(f6).
      VARIABLE LABELS Tid_Atg_Inkl 'Tid från åtgärd till inklusion'.
COMPUTE Tid_Atg_Inkl = datediff(InskrivDatum,UTDATUM,'days').
   EXECUTE.

*Ta bort åtgärder och diagnoser som inte varit före IVA med Sepsis.
SELECT IF Tid_Diagn_Inkl > 0.
   EXECUTE.



*Hitta Cytoadministreringar.
NUMERIC CytoAdm(f2).
NUMERIC CytoAdm2(f2).
   VARIABLE LABELS CytoAdm 'Cytoadministreringar -åtgärd- sista sex månaderna'.
   VARIABLE LABELS CytoAdm2 'Cytoadministreringar -åtgärd- sista 12 månaderna'.
   VALUE LABELS CytoAdm 0 'No' 1 'Yes'.
   COMPUTE AA = (CHAR.INDEX(UPCASE(Op),'DT107') > 0).
   COMPUTE BB = (CHAR.INDEX(UPCASE(Op),'DT108') > 0).
   COMPUTE CC = (CHAR.INDEX(UPCASE(Op),'DT112') > 0).
   COMPUTE DD = (CHAR.INDEX(UPCASE(Op),'DT116') > 0).
   COMPUTE EE = (CHAR.INDEX(UPCASE(Op),'DT135') > 0).
   IF AA > 0 or  BB > 0 or CC > 0 or DD > 0  or EE > 0 CytoAdm = 1.
```

```
    If CytoAdm = 1 CytoAdm2 = 1.
    If Tid_Atg_Inkl > 365 CytoAdm2 = 0.
    IF sysmis(CytoAdm2) CytoAdm2= 0.
  If Tid_Atg_Inkl > 182 CytoAdm = 0.
  IF sysmis(CytoAdm) CytoAdm= 0.
    EXECUTE.
  DELETE VARIABLES AA BB CC DD EE.
    EXECUTE.

*Hitta Strålbehandlingar.
NUMERIC Radiak(f2).
NUMERIC Radiak2(f2).
  VARIABLE LABELS Radiak 'Strålbehandlingar -åtgärd- sista sex månaderna'.
  VARIABLE LABELS Radiak2 'Strålbehandlingar -åtgärd- sista 12 månaderna'.
  VALUE LABELS Radiak 0 'No' 1 'Yes'.
  COMPUTE AA = (CHAR.INDEX(UPCASE(Op),'DV069') > 0).
  COMPUTE BB = (CHAR.INDEX(UPCASE(Op),'DV070') > 0).
  COMPUTE CC = (CHAR.INDEX(UPCASE(Op),'DV071') > 0).
  COMPUTE DD = (CHAR.INDEX(UPCASE(Op),'DV072') > 0).
  IF AA > 0 or  BB > 0 or CC > 0 or DD > 0 Radiak = 1.
  IF Radiak = 1 Radiak2 = 1.
    If Tid_Atg_Inkl > 365 Radiak2 = 0.
    IF sysmis(Radiak2) Radiak2= 0.
  If Tid_Atg_Inkl > 182 Radiak = 0.
  IF sysmis(Radiak) Radiak= 0.
    EXECUTE.
  DELETE VARIABLES AA BB CC DD.
    EXECUTE.
```

```
*Hitta Hemodialys.

NUMERIC HD_Cron(f2).

   VARIABLE LABELS HD_Cron 'Intermittent hemodialys -åtgärd- sista sex månaderna'.

   VALUE LABELS HD_Cron 0 'No' 1 'Yes'.

   COMPUTE AA = (CHAR.INDEX(UPCASE(Op),'DR016') > 0).

   IF AA > 0 HD_Cron = 1.

   If Tid_Atg_Inkl > 182 HD_Cron = 0.

   IF sysmis(HD_Cron) HD_Cron= 0.

      EXECUTE.

   DELETE VARIABLES AA.

      EXECUTE.


*Hitta
Diagnos////////////////////////////////////////////////////////////////////////////////////////////////////////
//////////////////////. Obs 5 årr!!! fixa datasetet!!!!!.

*OBS för diabetes II, övervikt och HT söks efter lm 5 år tillbaka!!!! Samma för åtärder för  njursvikt - HD PD´och övervikt.

*Hitta Ischemisk hjärtsjukdom.

NUMERIC IHD(f2).

   VARIABLE LABELS IHD 'Ischemisk hjärtsjukdom'.

   VALUE LABELS IHD 0 'No' 1 'Yes'.

   COMPUTE AA = (CHAR.INDEX(UPCASE(Diagnos),'I20') > 0).

   COMPUTE BB = (CHAR.INDEX(UPCASE(Diagnos),'I21') > 0).

   COMPUTE CC = (CHAR.INDEX(UPCASE(Diagnos),'I22') > 0).

   COMPUTE DD = (CHAR.INDEX(UPCASE(Diagnos),'I23') > 0).

   COMPUTE EE = (CHAR.INDEX(UPCASE(Diagnos),'I24') > 0).

   COMPUTE FF = (CHAR.INDEX(UPCASE(Diagnos),'I25') > 0).

   IF AA > 0 or  BB > 0 or CC > 0 or DD > 0 or EE > 0 or FF > 0 IHD = 1.

   IF sysmis(IHD) IHD= 0.

      EXECUTE.
```

```
   DELETE VARIABLES AA BB CC DD EE FF.
      EXECUTE.

*Hitta annan hjärtsjukdom.
NUMERIC OtherHD(f2).
   VARIABLE LABELS OtherHD 'Annan hjärtsjukdom - icke IHD'.
   VALUE LABELS OtherHD 0 'No' 1 'Yes'.
   COMPUTE AA = (CHAR.INDEX(UPCASE(Diagnos),'I3') > 0).
   COMPUTE BB = (CHAR.INDEX(UPCASE(Diagnos),'I4') > 0).
   COMPUTE CC = (CHAR.INDEX(UPCASE(Diagnos),'I5') > 0).
   COMPUTE DD = (CHAR.INDEX(UPCASE(Diagnos),'I01') > 0).
   COMPUTE EE = (CHAR.INDEX(UPCASE(Diagnos),'I05') > 0).
   COMPUTE FF = (CHAR.INDEX(UPCASE(Diagnos),'I06') > 0).
   COMPUTE GG = (CHAR.INDEX(UPCASE(Diagnos),'I07') > 0).
   COMPUTE HH = (CHAR.INDEX(UPCASE(Diagnos),'I08') > 0).
   COMPUTE II = (CHAR.INDEX(UPCASE(Diagnos),'I09') > 0).
   COMPUTE JJ = (CHAR.INDEX(UPCASE(Diagnos),'I11') > 0).
   COMPUTE KK = (CHAR.INDEX(UPCASE(Diagnos),'I13') > 0).
   COMPUTE LL = (CHAR.INDEX(UPCASE(Diagnos),'I27') > 0).
   IF AA > 0 or  BB > 0 or CC > 0 or DD > 0 or EE > 0 or FF > 0 or GG > 0 or HH > 0 or II > 0 or JJ > 0 or KK > 0 or LL
> 0 OtherHD = 1.
   IF sysmis(OtherHD) OtherHD= 0.
      EXECUTE.
   DELETE VARIABLES AA BB CC DD EE FF GG HH II JJ KK LL.
      EXECUTE.

*Hitta hypertoni.
NUMERIC Hypertens(f2).
   VARIABLE LABELS Hypertens 'Hypertoni'.
   VALUE LABELS Hypertens 0 'No' 1 'Yes'.
```

```
   COMPUTE AA = (CHAR.INDEX(UPCASE(Diagnos),'I1') > 0).
   IF AA > 0 Hypertens = 1.
   IF sysmis(Hypertens) Hypertens = 0.
      EXECUTE.
   DELETE VARIABLES AA.
      EXECUTE.

*Hitta Diabetes typ I.
NUMERIC Diabetes1(f2).
   VARIABLE LABELS Diabetes1 'Diabetes typ 1'.
   VALUE LABELS Diabetes1 0 'No' 1 'Yes'.
   COMPUTE AA = (CHAR.INDEX(UPCASE(Diagnos),'E10') > 0).
   IF AA > 0 Diabetes1 = 1.
   IF sysmis(Diabetes1) Diabetes1 = 0.
      EXECUTE.
   DELETE VARIABLES AA.
      EXECUTE.

*Hitta Diabetes typ II.
NUMERIC Diabetes2(f2).
   VARIABLE LABELS Diabetes2 'Diabetes typ 2'.
   VALUE LABELS Diabetes2 0 'No' 1 'Yes'.
   COMPUTE AA = (CHAR.INDEX(UPCASE(Diagnos),'E11') > 0).
      EXECUTE.
   IF AA > 0 Diabetes2 = 1.
   IF sysmis(Diabetes2) Diabetes2 = 0.
   EXECUTE.
   DELETE VARIABLES AA.
      EXECUTE.
```

```
*Hittta stroke.

NUMERIC Stroke(f2).

   VARIABLE LABELS Stroke 'Någon Cerebrovaskulär sjukdom'.

   VALUE LABELS Stroke 0 'No' 1 'Yes'.

   COMPUTE AA = (CHAR.INDEX(UPCASE(Diagnos),'I6') > 0).

   IF AA > 0 Stroke = 1.

   IF sysmis(Stroke) Stroke = 0.

      EXECUTE.

   DELETE VARIABLES AA.

      EXECUTE.


*Hitta kronisk njursvikt.

NUMERIC Njursvikt(f2).

   VARIABLE LABELS Njursvikt 'Njursvikt'.

   VALUE LABELS Njursvikt 0 'No' 1 'Yes'.

   COMPUTE N181 = (CHAR.INDEX(UPCASE(Diagnos),'N181') > 0).

   COMPUTE N182 = (CHAR.INDEX(UPCASE(Diagnos),'N182') > 0).

   COMPUTE N183 = (CHAR.INDEX(UPCASE(Diagnos),'N183') > 0).

   COMPUTE N184 = (CHAR.INDEX(UPCASE(Diagnos),'N184') > 0).

   COMPUTE N185 = (CHAR.INDEX(UPCASE(Diagnos),'N185') > 0).

   COMPUTE N189 = (CHAR.INDEX(UPCASE(Diagnos),'N189') > 0).

   *Hitta HD, PD senaste 5 åren.

      COMPUTE DR016 = (CHAR.INDEX(UPCASE(Op),'DR016') > 0).

      COMPUTE DR024 = (CHAR.INDEX(UPCASE(Op),'DR024') > 0).

      IF N181 > 0 or  N182 > 0 or  N183 > 0 or  N184 > 0 or  N185 > 0 or  N189 > 0 or  DR016 > 0 or DR024 > 0
Njursvikt = 1.

   IF sysmis(Njursvikt) Njursvikt = 0.

   RECODE N181 to DR024 (SYSMIS = 0).

      EXECUTE.
```

```
*Hitta KOL, inklusive emfysem och kronisk bronkit.
NUMERIC KOL(f2).
   VARIABLE LABELS KOL 'KOL, emfysem, kronisk bronkit'.
   VALUE LABELS KOL 0 'No' 1 'Yes'.
   COMPUTE AA = (CHAR.INDEX(UPCASE(Diagnos),'J41') > 0).
   COMPUTE BB = (CHAR.INDEX(UPCASE(Diagnos),'J42') > 0).
   COMPUTE CC = (CHAR.INDEX(UPCASE(Diagnos),'J43') > 0).
   COMPUTE DD = (CHAR.INDEX(UPCASE(Diagnos),'J44') > 0).
   IF AA > 0 or  BB > 0 or CC > 0 or DD > 0 KOL = 1.
   IF sysmis(KOL) KOL= 0.
      EXECUTE.
   DELETE VARIABLES AA BB CC DD.
      EXECUTE.

*Hitta Astma.
NUMERIC Astma(f2).
   VARIABLE LABELS Astma 'Astma'.
   VALUE LABELS Astma 0 'No' 1 'Yes'.
   COMPUTE AA = (CHAR.INDEX(UPCASE(Diagnos),'J45') > 0).
   COMPUTE BB = (CHAR.INDEX(UPCASE(Diagnos),'J46') > 0).
   IF AA > 0 or  BB > 0 Astma = 1.
   IF sysmis(Astma) Astma = 0.
      EXECUTE.
   DELETE VARIABLES AA BB.
      EXECUTE.

*Hitta fetma inklusive överviktskirurgi.
NUMERIC Fetma(f2).
```

```
VARIABLE LABELS Fetma 'Fetma'.
VALUE LABELS Fetma 0 'No' 1 'Yes'.
COMPUTE AA = (CHAR.INDEX(UPCASE(Diagnos),'E66') > 0).
*Hitta Överviktskirurgi.
  COMPUTE BB = (CHAR.INDEX(UPCASE(Op),'JDF') > 0).
  COMPUTE CC = (CHAR.INDEX(UPCASE(Op),'JFD03') > 0).
  IF AA > 0 or  BB > 0 or CC > 0 Fetma = 1.
IF sysmis(Fetma) Fetma = 0.
  EXECUTE.
DELETE VARIABLES AA BB CC.
  EXECUTE.


*Hitta Immunsupprimerande sjukdom eller efter åtgärd.
NUMERIC Immunsupprimerad(f2).
VARIABLE LABELS Immunsupprimerad 'Immunsupprimerad'.
VALUE LABELS Immunsupprimerad 0 'No' 1 'Yes'.
COMPUTE AA = (CHAR.INDEX(UPCASE(Diagnos),'D71') > 0).
COMPUTE BB = (CHAR.INDEX(UPCASE(Diagnos),'D80') > 0).
COMPUTE CC = (CHAR.INDEX(UPCASE(Diagnos),'D81') > 0).
COMPUTE DD = (CHAR.INDEX(UPCASE(Diagnos),'D82') > 0).
COMPUTE EE = (CHAR.INDEX(UPCASE(Diagnos),'D83') > 0).
COMPUTE FF = (CHAR.INDEX(UPCASE(Diagnos),'D84') > 0).
COMPUTE GG = (CHAR.INDEX(UPCASE(Diagnos),'D89') > 0).
IF AA > 0 or  BB > 0 or CC > 0 or DD > 0 or EE > 0 or FF > 0 or GG > 0  Immunsupprimerad = 1.
*Hitta immunsupprimerande åtgärder lm senaste 6 månaderna.
IF CytoAdm = 1 or Radiak = 1 Immunsupprimerad = 1.
IF sysmis(Immunsupprimerad)  Immunsupprimerad = 0.
  EXECUTE.
DELETE VARIABLES AA BB CC DD EE FF GG.
```

```
      EXECUTE.


*Hitta aktiv solid tumör senaste året dvs diagnossatt Senaste 12 mån..
NUMERIC Cancer_dia(f2).
NUMERIC CancerDiagn(f2).
   VARIABLE LABELS Cancer_dia 'Cancer'.
   VALUE LABELS Cancer_dia 0 'No' 1 'Yes'.
   COMPUTE AA = (CHAR.INDEX(UPCASE(Diagnos),'C0') > 0).
   COMPUTE BB = (CHAR.INDEX(UPCASE(Diagnos),'C1') > 0).
   COMPUTE CC = (CHAR.INDEX(UPCASE(Diagnos),'C2') > 0).
   COMPUTE DD = (CHAR.INDEX(UPCASE(Diagnos),'C3') > 0).
   COMPUTE EE = (CHAR.INDEX(UPCASE(Diagnos),'C4') > 0).
   COMPUTE FF = (CHAR.INDEX(UPCASE(Diagnos),'C5') > 0).
   COMPUTE GG = (CHAR.INDEX(UPCASE(Diagnos),'C6') > 0).
   COMPUTE HH = (CHAR.INDEX(UPCASE(Diagnos),'C7') > 0).
   COMPUTE II = (CHAR.INDEX(UPCASE(Diagnos),'C80') > 0).
   COMPUTE JJ = (CHAR.INDEX(UPCASE(Diagnos),'C97') > 0).
    IF AA > 0 or  BB > 0 or CC > 0 or DD > 0 or EE > 0 or FF > 0 or GG > 0 or HH > 0 or II > 0 or JJ > 0 CancerDiagn =
1.
   IF Tid_Diagn_Inkl > 365 CancerDiagn = 0.
   If CancerDiagn = 1 Cancer_dia = 1.
   IF sysmis(Cancer_dia) Cancer_dia = 0.
      EXECUTE.
   DELETE VARIABLES AA to JJ CancerDiagn.
      EXECUTE.


*Hitta aktiv blodcancer senaste året dvs diagnossatt Senaste 12 mån..
NUMERIC BlodCancer(f2).
NUMERIC BlodCancerDiagn(f2).
   VARIABLE LABELS BlodCancer 'Hematologisk malignitet'.
```

```
   VALUE LABELS BlodCancer 0 'No' 1 'Yes'.
   COMPUTE AA = (CHAR.INDEX(UPCASE(Diagnos),'C81') > 0).
   COMPUTE BB = (CHAR.INDEX(UPCASE(Diagnos),'C82') > 0).
   COMPUTE CC = (CHAR.INDEX(UPCASE(Diagnos),'C83') > 0).
   COMPUTE DD = (CHAR.INDEX(UPCASE(Diagnos),'C84') > 0).
   COMPUTE EE = (CHAR.INDEX(UPCASE(Diagnos),'C85') > 0).
   COMPUTE FF = (CHAR.INDEX(UPCASE(Diagnos),'C86') > 0).
   COMPUTE GG = (CHAR.INDEX(UPCASE(Diagnos),'C88') > 0).
   COMPUTE HH = (CHAR.INDEX(UPCASE(Diagnos),'C90') > 0).
   COMPUTE II = (CHAR.INDEX(UPCASE(Diagnos),'C91') > 0).
   COMPUTE JJ = (CHAR.INDEX(UPCASE(Diagnos),'C92') > 0).
   COMPUTE KK = (CHAR.INDEX(UPCASE(Diagnos),'C93') > 0).
   COMPUTE LL = (CHAR.INDEX(UPCASE(Diagnos),'C94') > 0).
   COMPUTE MM = (CHAR.INDEX(UPCASE(Diagnos),'C95') > 0).
   COMPUTE NN = (CHAR.INDEX(UPCASE(Diagnos),'C96') > 0).
    IF AA > 0 or  BB > 0 or CC > 0 or DD > 0 or EE > 0 or FF > 0 or GG > 0 or HH > 0 or II > 0 or JJ > 0 or KK > 0 or LL
> 0 or MM > 0 or NN > 0 BlodCancerDiagn = 1.
   IF Tid_Diagn_Inkl > 365 BlodCancerDiagn = 0.
   If BlodCancerDiagn = 1 BlodCancer = 1.
   IF sysmis(BlodCancer) BlodCancer= 0.
      EXECUTE.
   DELETE VARIABLES AA to NN BlodCancerDiagn.
      EXECUTE.


*Hitta inflammatorisk sjukdom.
NUMERIC Inflam(f2).
   VARIABLE LABELS Inflam 'Systeminflammatorisk sjukdom'.
   VALUE LABELS Inflam 0 'No' 1 'Yes'.
   COMPUTE AA = (CHAR.INDEX(UPCASE(Diagnos),'M3') > 0).
   COMPUTE BB = (CHAR.INDEX(UPCASE(Diagnos),'M05') > 0).
```

```
   COMPUTE CC = (CHAR.INDEX(UPCASE(Diagnos),'M06') > 0).
   COMPUTE DD = (CHAR.INDEX(UPCASE(Diagnos),'M07') > 0).
   COMPUTE EE = (CHAR.INDEX(UPCASE(Diagnos),'M08') > 0).
   COMPUTE FF = (CHAR.INDEX(UPCASE(Diagnos),'M09') > 0).
   COMPUTE GG = (CHAR.INDEX(UPCASE(Diagnos),'M10') > 0).
   COMPUTE HH = (CHAR.INDEX(UPCASE(Diagnos),'M11') > 0).
   COMPUTE II = (CHAR.INDEX(UPCASE(Diagnos),'M12') > 0).
   COMPUTE JJ = (CHAR.INDEX(UPCASE(Diagnos),'M13') > 0).
   COMPUTE KK = (CHAR.INDEX(UPCASE(Diagnos),'M14') > 0).
   IF AA > 0 or  BB > 0 or CC > 0 or DD > 0 or EE > 0 or FF > 0 or GG > 0 or HH > 0 or II > 0 or JJ > 0 or KK > 0 Inflam
= 1.
   IF sysmis(Inflam) Inflam= 0.
      EXECUTE.
   DELETE VARIABLES AA BB CC DD EE FF GG HH II JJ KK.
      EXECUTE.
*Hitta transplanterade.
NUMERIC Transplanterad(f2).
   VARIABLE LABELS Transplanterad 'Transplanterad'.
   VALUE LABELS Transplanterad 0 'No' 1 'Yes'.
   COMPUTE AA = (CHAR.INDEX(UPCASE(Diagnos),'Z940') > 0).
   COMPUTE BB = (CHAR.INDEX(UPCASE(Diagnos),'Z941') > 0).
   COMPUTE CC = (CHAR.INDEX(UPCASE(Diagnos),'Z942') > 0).
   COMPUTE DD = (CHAR.INDEX(UPCASE(Diagnos),'Z943') > 0).
   COMPUTE EE = (CHAR.INDEX(UPCASE(Diagnos),'Z944') > 0).

   IF AA > 0 or  BB > 0 or CC > 0 or DD > 0 or EE > 0 Transplanterad = 1.
   IF sysmis(Transplanterad)  Transplanterad = 0.
      EXECUTE.
   DELETE VARIABLES AA BB CC DD EE.
      EXECUTE.
```

```
DELETE VARIABLES CounterVTF.
   EXECUTE.
SORT CASES ID(a) INDATUM(a) UTDATUM(a).
   NUMERIC CounterVTF(f3).
   IF $casenum = 1 or ID ne lag(ID) CounterVTF = 1.
   IF ID = lag(ID) CounterVTF = lag(CounterVTF) + 1.
      EXECUTE.
SORT CASES ID(a) CounterVTF(d).
EXECUTE.
*Skapa en rad per individ.
If ID = lag(ID) CytoAdm = CytoAdm + lag(CytoAdm).
IF CytoAdm > 0 CytoAdm = 1.
If ID = lag(ID) CytoAdm2 = CytoAdm2 + lag(CytoAdm2).
IF CytoAdm2 > 0 CytoAdm2 = 1.
If ID = lag(ID) Radiak = Radiak + lag(Radiak).
IF Radiak > 0 Radiak = 1.
If ID = lag(ID) Radiak2 = Radiak2 + lag(Radiak2).
IF Radiak2 > 0 Radiak2 = 1.
If ID = lag(ID) HD_Cron = HD_Cron + lag(HD_Cron).
IF HD_Cron > 0 HD_Cron = 1.
If ID = lag(ID) IHD = IHD + lag(IHD).
IF IHD > 0 IHD = 1.
If ID = lag(ID) OtherHD = OtherHD + lag(OtherHD).
IF OtherHD > 0 OtherHD = 1.
If ID = lag(ID) Hypertens = Hypertens + lag(Hypertens).
IF Hypertens > 0 Hypertens = 1.
If ID = lag(ID) Diabetes1 = Diabetes1 + lag(Diabetes1).
IF Diabetes1 > 0 Diabetes1 = 1.
```

If ID = lag(ID) Diabetes2 = Diabetes2 + lag(Diabetes2).

IF Diabetes2 > 0 Diabetes2 = 1.

If ID = lag(ID) Stroke = Stroke + lag(Stroke).

IF Stroke > 0 Stroke = 1.

If ID = lag(ID) Njursvikt = Njursvikt + lag(Njursvikt).

IF Njursvikt > 0 Njursvikt = 1.

If ID = lag(ID) N181 = N181 + lag(N181).

IF N181 > 0 N181 = 1.

If ID = lag(ID) N182 = N182 + lag(N182).

IF N182 > 0 N182 = 1.

If ID = lag(ID) N183 = N183 + lag(N183).

IF N183 > 0 N183 = 1.

If ID = lag(ID) N184 = N184 + lag(N184).

IF N184 > 0 N184 = 1.

If ID = lag(ID) N185 = N185 + lag(N185).

IF N185 > 0 N185 = 1.

If ID = lag(ID) N189 = N189 + lag(N189).

IF N189 > 0 N189 = 1.

If ID = lag(ID) DR016 = DR016 + lag(DR016).

IF DR016 > 0 DR016 = 1.

If ID = lag(ID) DR024 = DR024 + lag(DR024).

IF DR024 > 0 DR024 = 1.

If ID = lag(ID) KOL = KOL + lag(KOL).

IF KOL > 0 KOL = 1.

If ID = lag(ID) Astma = Astma + lag(Astma).

IF Astma > 0 Astma = 1.

If ID = lag(ID) Fetma = Fetma + lag(Fetma).

IF Fetma > 0 Fetma = 1.

If ID = lag(ID) Immunsupprimerad = Immunsupprimerad + lag(Immunsupprimerad).

```
IF Immunsupprimerad > 0 Immunsupprimerad = 1.

If ID = lag(ID) Cancer_dia = Cancer_dia + lag(Cancer_dia).

IF Cancer_dia > 0 Cancer_dia = 1.

If ID = lag(ID) BlodCancer = BlodCancer + lag(BlodCancer).

IF BlodCancer > 0 BlodCancer = 1.

If ID = lag(ID) Inflam = Inflam + lag(Inflam).

IF Inflam > 0 Inflam = 1.

If ID = lag(ID) Transplanterad = Transplanterad + lag(Transplanterad).

IF Transplanterad > 0 Transplanterad = 1.



*Spara ett vtf per individ.

  EXECUTE.

SELECT IF CounterVTF = 1.

  EXECUTE.


ADD FILES FILE*

  /KEEP ID CytoAdm to Transplanterad.

    EXECUTE.


SAVE OUTFILE 'ARDS_Comorb.sav'.

  DATASET NAME ARDS_Comorb.

GET FILE 'ARDS_temp.sav'.

  DATASET NAME SIR WINDOW=FRONT.


DATASET ACTIVATE SIR.

MATCH FILES /FILE=*

 /FILE='ARDS_Comorb'

 /BY ID.
```

```
EXECUTE.


DATASET ACTIVATE SIR.
MATCH FILES /FILE=*
  /TABLE='ARDS_CCI'
  /BY ID.
EXECUTE.
    DATASET CLOSE ARDS_CCI.
    DATASET CLOSE ARDS_Comorb.
    DATASET ACTIVATE SIR.


RECODE CytoAdm to CCI (SYSMIS = 0) (ELSE = COPY).
    EXECUTE.
SORT CASES ID(a).


NUMERIC Sepsis(f2)
NUMERIC COVID(f2).
NUMERIC COVID2(f2).
        VALUE LABELS ARDS 0 'No ARDS' 1 'ARDS'.
        VALUE LABELS Sepsis 0 'No Sepsis' 1 'Sepsis'.
        VALUE LABELS Covid 0 'No Covid' 1 'Covid'.
        VARIABLE LABELS COVID2 'Covid också'.
    COMPUTE ARDS = 1.
    COMPUTE Sepsis = 0.
    COMPUTE COVID = 0.
    COMPUTE COVID2 = 0.
        EXECUTE.
SAVE OUTFILE 'TEMP12.sav'.
SORT CASES ID.
```

```
Get file 'Y:\Original_SPSS\ut_par_sv_8996_2017.sav'.

DATASET NAME Soc WINDOW=FRONT.

DATASET ACTIVATE Soc.


RENAME VARIABLES (lopnr LKF Kon = ID L Kon).

   FORMATS ID(f10).

      EXECUTE.


STRING LKF(a10).

COMPUTE LKF = concat(rtrim(LK),' ',rtrim(L)).

   EXECUTE.

   DELETE VARIABLES LK L.

      EXECUTE.

SORT CASES ID(a) INDATUM(a) UTDATUM(a).

   NUMERIC CounterVTF(f3).

   IF $casenum = 1 or ID ne lag(ID) CounterVTF = 1.

   IF ID = lag(ID) CounterVTF = lag(CounterVTF) + 1.

      EXECUTE.

SELECT IF CounterVTF = 1.

EXECUTE.


ADD FILES FILE*

   /KEEP ID SENUTV.

      EXECUTE.

DATASET ACTIVATE SIR.

MATCH FILES /FILE=*

  /FILE='Soc'

  /BY ID.
```

```
EXECUTE.

SELECT IF ARDS = 1.
EXECUTE.

   DATASET NAME ARDS.
   DATASET CLOSE Soc.
   DATASET ACTIVATE ARDS.

SELECT IF InskrTid >0.
   EXECUTE.
COMPUTE ID_ARDS = ID.
   EXECUTE.
COMPUTE ID = $casenum + 300000.
   EXECUTE.

ADD FILES FILE*
/KEEP ID Alder Kon InskrivDatum UtskrivDatum SjukhusTyp Opererad SAPS3Score SAPS3_u_comorb SAPS3ScoreBoxIII
Operationstyp APACHE_Score APACHE_AkutFysPoang IMV_tid CRRT_tid  IMV CRRT ICU_LoS
CytoAdm CytoAdm2 Radiak Radiak2 HD_Cron IHD OtherHD Hypertens Diabetes1 Diabetes2 Stroke Njursvikt N181 N182 N183 N184 N185
N189 DR016 DR024 KOL Astma Fetma Immunsupprimerad Cancer_dia BlodCancer Inflam Transplanterad
CCI COVID COVID2 Sepsis ARDS DeadICU_n DeathDate_ICU Dead_n DeathDate SENUTV ID_ARDS.
   EXECUTE.

*Ensa dataseten.
DATASET ACTIVATE ARDS.

RENAME VARIABLES (InskrivDatum Alder = InskrDat Age).
```

```
   EXECUTE.
NUMERIC Sex(f1).
   VARIABLE LABELS Sex 'Sex at ICU admission'.
   VALUE LABELS Sex 1 'Man' 2 'Kvinna'.
   IF Kon = 'M' Sex = 1.
   IF Kon = 'K' Sex = 2.
FORMATS Age(f3).
   VARIABLE LABELS Age 'Age at ICU admission'.
IF Opererad = 'Ja-elektivt' Opererad = 'Ja-elek'.
IF Opererad = 'Ja-akut' Opererad = 'Ja-akut'.
ALTER TYPE Opererad(a7).
   EXECUTE.

DESCRIPTIVES ID.
SELECT IF Age > 17.
DESCRIPTIVES ID.
FREQUENCIES COVID COVID2 Sepsis ARDS.
SAVE OUTFILE 'ARDS_komplett.sav'.

DATASET CLOSE all.
* Encoding: UTF-8.
CD 'Z:/'.
DATASET CLOSE all.
GET FILE 'Covid_komplett.sav'.
      DATASET NAME Covid WINDOW=FRONT.
         FREQUENCIES Sepsis2 ARDS2.
GET FILE 'Sepsis_komplett.sav'.
   DATASET NAME Sepsis WINDOW=FRONT.
GET FILE 'ARDS_komplett.sav'.
```

```
   DATASET NAME ARDS WINDOW=FRONT.
*Merge Sepsis ARDS COVID.
DATASET ACTIVATE Sepsis.
ADD FILES /FILE=*
  /FILE='ARDS'.
EXECUTE.
   DATASET NAME SepsisARDS.
   DATASET CLOSE ARDS.
   DATASET ACTIVATE SepsisARDS.
*Find overlapping individuals Sepsis - ARDS.
RECODE ID_Sepsis ID_ARDS (SYSMIS = 0) (ELSE = COPY).
COMPUTE ID_TEMP = ID_Sepsis + ID_ARDS.
   EXECUTE.
SORT CASES ID_TEMP(a).
   IF $casenum = 1 or ID_TEMP ne lag(ID_TEMP) Count = 1.
   IF ID_TEMP = lag(ID_TEMP) Count = lag(Count) + 1.
      EXECUTE.
   IF Count = 2 Overlap = 1.
      EXECUTE.
SORT CASES ID_TEMP(a) Count(d).
   IF ID_TEMP = lag(ID_TEMP) and lag(Count) = 2 Overlap = 1.
      EXECUTE.
   VARIABLE LABELS Overlap 'Individual is both in ARDS and Sepsis cohort'.
FREQUENCIES Overlap.


RECODE ARDS SEPSIS Sepsis_grad COVID COVID2 Overlap CCI CytoAdm to Transplanterad (SYSMIS = 0) (ELSE =
COPY).
   IF SAPS3Score> 0 and Apache_Score = 0 Apache_Score = $sysmis.
   IF SAPS3Score> 0 and APACHE_AkutFysPoang = 0 APACHE_AkutFysPoang = $sysmis.
   EXECUTE.
```

```
*Kontrollera. Count should only have 1. Dead... should have no missing values.
SORT CASES ID(a).
NUMERIC Count2(f2).
   IF $casenum = 1 or ID ne lag(ID) Count2 = 1.
   IF sysmis(Count2) Count2 = lag(Count2) + 1.
      EXECUTE.
FREQUENCIES Count2.



   ADD FILES FILE*
   /KEEP ID Age Sex SENUTV InskrDat SjukhusTyp Opererad SAPS3Score SAPS3_u_comorb SAPS3ScoreBoxIII
APACHE_Score
   APACHE_AkutFysPoang IMV CRRT IMV_tid CRRT_tid ICU_LoS IHD OtherHD Hypertens
   Diabetes1 Diabetes2 Stroke Njursvikt KOL Astma Fetma Immunsupprimerad Cancer_dia BlodCancer
   Inflam Transplanterad CCI COVID COVID2 Sepsis Sepsis_grad ARDS DeadICU_n DeathDate_ICU Dead_n
DeathDate Overlap.
      EXECUTE.
RECODE SjukhusTyp ('LÃ¤nssjukhus' = 'Länssjukhus') ('LÃ¤nsdelssjukhus' = 'Länsdelssjukhus').

RENAME VARIABLES ( SAPS3ScoreBoxIII IMV_tid CRRT_tid = SAPS3_Box3 IMV_time CRRT_time).

DATASET ACTIVATE Covid.
   ADD FILES FILE*
   /KEEP ID Age Sex SENUTV InskrDat ICU_LoS SjukhusTyp Opererad SAPS3Score SAPS3_u_comorb SAPS3_Box3
PF IMV CRRT IMV_time CRRT_time
   DeadICU_n DeathDate_ICU Dead_n DeathDate CCI IHD OtherHD Hypertens Diabetes1
   Diabetes2 Stroke Njursvikt KOL Astma Fetma Immunsupprimerad Cancer_dia BlodCancer Inflam
   Transplanterad COVID ARDS Sepsis COVID2.

ALTER TYPE SjukhusTyp(a18).
```

```
DATASET ACTIVATE SepsisArds.
ADD FILES /FILE=*
 /FILE='Covid'.
EXECUTE.
  DATASET NAME Kohort.
  DATASET CLOSE Covid.
  DATASET ACTIVATE Kohort.

SORT CASES ID(a).
FREQUENCIES Covid ARDS Sepsis.
DESCRIPTIVES ID.

RECODE ARDS SEPSIS Sepsis_grad COVID COVID2 Overlap CCI IMV CRRT IHD to Transplanterad (SYSMIS = 0)
(ELSE = COPY).
  EXECUTE.
*Time_at_risk.
NUMERIC TimeAtRisk60(f4).
  VARIABLE LABELS TimeAtRisk60 'TimeAtRisk for 60 day mortality. Days'.
NUMERIC Dead60_n(f2).
  IF Dead_n = 1 TimeAtRisk60 = datediff(DeathDate,InskrDat,'days').
  IF TimeAtRisk60 < 61 Dead60_n = 1.
  IF TimeAtRisk60  > 60  TimeAtRisk60 = 60.
  IF sysmis(TimeAtRisk60) TimeAtRisk60 = 60.
  COMPUTE TimeAtRisk_ICU = ICU_LoS.
  VARIABLE LABELS TimeAtRisk_ICU 'Time at risk for ICU-mortality'.
  VARIABLE LABELS Dead60_n 'Dead at 60 days'.
RECODE Dead60_n (SYSMIS = 0) (ELSE = COPY).
  EXECUTE.
FREQUENCIES Dead60_n Dead_n DeadICU_n.
```

```
DESCRIPTIVES TimeAtRisk_ICU TimeAtRisk60.

GRAPH

  /HISTOGRAM=TimeAtRisk_ICU.

GRAPH

  /HISTOGRAM=TimeAtRisk60.



*Skapa sjukdomsvariabel.

NUMERIC CSA(f2).

        VARIABLE LABELS CSA 'COVID, Sepsis or ARDS'.

        VALUE LABELS CSA 1 'COVID' 2 'Sepsis' 3 'ARDS'.

    IF COVID = 1 CSA = 1.

    IF Sepsis = 1 CSA = 2.

    IF ARDS = 1 CSA = 3.

FREQUENCIES CSA.



    SORT CASES CSA(a).

        SPLIT FILE by CSA.

FREQUENCIES Dead60_n DeadICU_n.

DESCRIPTIVES SAPS3Score CCI ICU_LoS.

     SPLIT FILE off.

    SORT CASES ID(a).



*Utfallsvariabler.

NUMERIC CovSeps(f2).

    IF COVID = 1 CovSeps = 1.

    IF Sepsis = 1 CovSeps = 0.
```

```
NUMERIC CovArds(f2).
   IF COVID = 1 CovArds = 1.
   IF ARDS = 1 CovArds = 0.
      EXECUTE.

*Tidsvariabel.
NUMERIC Time(f6).
   COMPUTE Start = date.dmy(01,01,2011).
   COMPUTE Time = Datediff(InskrDat,start,'month').
      EXECUTE.

FREQUENCIES CovSeps CovArds.
DESCRIPTIVES Time.
   DELETE VARIABLES Start.
      EXECUTE.
SAVE OUTFILE 'C_S_A_Tab1.sav'.

*Fil till R.

*Fixa CCI (få värden över 10...).
NUMERIC CCI_fix(f2).
   COMPUTE CCI_fix = CCI.
   RECODE CCI_fix (10 thru 13 = 10) (ELSE = COPY).
FREQUENCIES CCI_fix.
*Skapa variabler med Covid + sepsis utan ards(sepsis) och covid + ards utan sepsis(Ards).
NUMERIC CovSeps2(f2).
   If Sepsis = 1 CovSeps2 = 0.
   If Overlap = 1 CovSeps2 = $SYSMIS.
   IF Covid = 1 CovSeps2 = 1.
```

```
EXECUTE.
NUMERIC CovArds2(f2).
   If ARDS = 1 CovArds2 = 0.
   If Overlap = 1 CovArds2 = $SYSMIS.
   IF COVID = 1 CovArds2 = 1.
EXECUTE.

ADD FILES File*
   /KEEP ID CovSeps CovArds CovSeps2 CovArds2 COVID Overlap Sepsis ARDS CCI_fix
   Time TimeAtRisk_ICU DeadICU_n SAPS3_u_comorb SAPS3_Box3 APACHE_AkutFysPoang TimeAtRisk60
Dead60_n Age Sex IHD OtherHD Hypertens
   Diabetes1 Diabetes2 Stroke Njursvikt KOL Astma Fetma Immunsupprimerad Cancer_dia BlodCancer Inflam
   Transplanterad CSA SjukhusTyp.
   EXECUTE.

SAVE OUTFILE 'C_S_A.sav'.

SORT CASES APACHE_AkutFysPoang(d).

SAVE TRANSLATE OUTFILE='Z:\C_S_A.csv'
  /TYPE=CSV
  /ENCODING='UTF8'
  /MAP
  /REPLACE
  /FIELDNAMES
  /CELLS=LABELS.

DATASET CLOSE all.
cd 'Z:/'.
GET FILE 'C_S_A_Tab1.sav'.
```

```
   DATASET NAME Tabell1 WINDOW=FRONT.
   DATASET ACTIVATE Tabell1.

NUMERIC Keep(f1).
IF CovSeps =1 Keep = 1.
IF CovSeps = 0 Keep = 1.

SELECT IF Keep =1.
   EXECUTE.
NUMERIC Op(f2).
   IF Opererad =  "Ja-akut" or Opererad = "Ja-elekt" Op = 1.
   IF Opererad = "Nej" Op = 0.
   EXECUTE.

SORT CASES by CovSeps.
   SPLIT FILE by CovSeps.
*Tabell1.
FREQUENCIES SjukhusTyp Sex Op.
FREQUENCIES VARIABLES=Age SAPS3Score CCI ICU_LoS
  /NTILES=4
   /FORMAT=NOTABLE
  /STATISTICS=STDDEV MEAN
  /ORDER=ANALYSIS.

*Tabell3.
SELECT IF Dead60_n = 1.
   EXECUTE.
FREQUENCIES SjukhusTyp Sex Op.
```

```
FREQUENCIES VARIABLES=Age SAPS3Score CCI ICU_LoS
  /NTILES=4
    /FORMAT=NOTABLE
  /STATISTICS=STDDEV MEAN
  /ORDER=ANALYSIS.

SPLIT FILE off.
DATASET CLOSE Tabell1.

GET FILE 'C_S_A_Tab1.sav'.
    DATASET NAME Tabell1 WINDOW=FRONT.
    DATASET ACTIVATE Tabell1.

NUMERIC Keep(f1).
IF CovARDS =1 Keep = 1.
IF CovARDS = 0 Keep = 1.

SELECT IF Keep =1.
    EXECUTE.
NUMERIC Op(f2).
    IF Opererad =  "Ja-akut" or Opererad = "Ja-elekt" Op = 1.
    IF Opererad = "Nej" Op = 0.
    EXECUTE.

SORT CASES by CovARDS.
    SPLIT FILE by CovARDS.
*Tabell1.
FREQUENCIES SjukhusTyp Sex Op.
FREQUENCIES VARIABLES=Age SAPS3Score CCI ICU_LoS
```

```
  /NTILES=4
    /FORMAT=NOTABLE
  /STATISTICS=STDDEV MEAN
  /ORDER=ANALYSIS.



*Tabell3.
SELECT IF Dead60_n = 1.
    EXECUTE.
FREQUENCIES SjukhusTyp Sex Op.
FREQUENCIES VARIABLES=Age SAPS3Score CCI ICU_LoS
  /NTILES=4
    /FORMAT=NOTABLE
  /STATISTICS=STDDEV MEAN
  /ORDER=ANALYSIS.
SPLIT FILE off.
DATASET CLOSE Tabell1.

*//////////////////////////////////////////////////////////////////////////////////////////////////////////////
//////////////.
*P-värden.....
DATASET CLOSE Tabell1.

GET FILE 'C_S_A_Tab1.sav'.
    DATASET NAME Tabell1 WINDOW=FRONT.
    DATASET ACTIVATE Tabell1.

NUMERIC Op(f2).
    IF Opererad =  "Ja-akut" or Opererad = "Ja-elekt" Op = 1.
    IF Opererad = "Nej" Op = 0.
```

```
   EXECUTE.

SORT CASES ID(a).

*Sepsis.
*M-W.
NPAR TESTS
  /M-W= Age SAPS3Score CCI ICU_LoS BY CovSeps(1 0)
  /STATISTICS=DESCRIPTIVES QUARTILES
  /MISSING ANALYSIS.

*Sepsis.
*Chi2.
CROSSTABS
  /TABLES=CovSeps BY SjukhusTyp Sex Op IMV Dead60_n
  /FORMAT=AVALUE TABLES
  /STATISTICS=CHISQ
  /CELLS=COUNT ROW
  /COUNT ROUND CELL
  /METHOD=EXACT TIMER(2).

*ARDS.
*M-W.
NPAR TESTS
  /M-W= Age SAPS3Score CCI ICU_LoS BY CovARDS(1 0)
  /STATISTICS=DESCRIPTIVES QUARTILES
  /MISSING ANALYSIS.

*ARDS.
```

```
*Chi2..
CROSSTABS
  /TABLES=CovARDS BY SjukhusTyp Sex Op IMV Dead60_n
  /FORMAT=AVALUE TABLES
  /STATISTICS=CHISQ
  /CELLS=COUNT ROW
  /COUNT ROUND CELL
  /METHOD=EXACT TIMER(2).

*Tabell2.
*Sepsis.
CROSSTABS
  /TABLES=CovSeps BY IHD OtherHD Hypertens Diabetes1 Diabetes2
    Stroke Njursvikt KOL Astma Fetma Immunsupprimerad Cancer_dia
    BlodCancer Inflam Transplanterad
  /FORMAT=AVALUE TABLES
  /STATISTICS=CHISQ
  /CELLS=COUNT ROW
  /COUNT ROUND CELL
  /METHOD=EXACT TIMER(2).

*Tabell2.
*ARDS.
CROSSTABS
  /TABLES=CovARDS BY IHD OtherHD Hypertens Diabetes1 Diabetes2
    Stroke Njursvikt KOL Astma Fetma Immunsupprimerad Cancer_dia
    BlodCancer Inflam Transplanterad
  /FORMAT=AVALUE TABLES
  /STATISTICS=CHISQ
```

```
  /CELLS=COUNT ROW
  /COUNT ROUND CELL
  /METHOD=EXACT TIMER(2).


*Tabell3.
SELECT IF Dead60_n = 1.
   EXECUTE.


SORT CASES ID(a).

*Tabell3.
*Sepsis.
*M-W.
NPAR TESTS
  /M-W= Age SAPS3Score CCI ICU_LoS BY CovSeps(1 0)
  /STATISTICS=DESCRIPTIVES QUARTILES
  /MISSING ANALYSIS.

*Sepsis.
*Chi2.
CROSSTABS
  /TABLES=CovSeps BY SjukhusTyp Sex Op
  /FORMAT=AVALUE TABLES
  /STATISTICS=CHISQ
  /CELLS=COUNT ROW
  /COUNT ROUND CELL
```

```
  /METHOD=EXACT TIMER(2).


*Tabell3.
*ARDS.
*M-W.
NPAR TESTS
  /M-W= Age SAPS3Score CCI ICU_LoS BY CovARDS(1 0)
  /STATISTICS=DESCRIPTIVES QUARTILES
  /MISSING ANALYSIS.


*ARDS.
*Chi2..
CROSSTABS
  /TABLES=CovARDS BY SjukhusTyp Sex Op
  /FORMAT=AVALUE TABLES
  /STATISTICS=CHISQ
  /CELLS=COUNT ROW
  /COUNT ROUND CELL
  /METHOD=EXACT TIMER(2).


*Tabell3 Comorb.
*Sepsis.
CROSSTABS
  /TABLES=CovSeps BY IHD OtherHD Hypertens Diabetes1 Diabetes2
    Stroke Njursvikt KOL Astma Fetma Immunsupprimerad Cancer_dia
    BlodCancer Inflam Transplanterad
  /FORMAT=AVALUE TABLES
  /STATISTICS=CHISQ
  /CELLS=COUNT ROW
```

```
  /COUNT ROUND CELL
  /METHOD=EXACT TIMER(2).


*Tabell3 Comorb.
*Tabell3.
*ARDS.
CROSSTABS
  /TABLES=CovARDS BY IHD OtherHD Hypertens Diabetes1 Diabetes2
    Stroke Njursvikt KOL Astma Fetma Immunsupprimerad Cancer_dia
    BlodCancer Inflam Transplanterad
  /FORMAT=AVALUE TABLES
  /STATISTICS=CHISQ
  /CELLS=COUNT ROW
  /COUNT ROUND CELL
  /METHOD=EXACT TIMER(2).
```
